# Electrochemical Iodine‐Mediated Oxidation of Enamino‐Esters to 2*H*‐Azirine‐2‐Carboxylates Supported by *Design of Experiments*

**DOI:** 10.1002/chem.202001465

**Published:** 2020-07-14

**Authors:** Emre Babaoglu, Gerhard Hilt

**Affiliations:** ^1^ Institut für Chemie Carl von Ossietzky Universität Oldenburg Carl-von-Ossietzky Strasse 9–11 26129 Oldenburg Germany; ^2^ Fachbereich Chemie Philipps-Universität Marburg Hans-Meerwein-Strasse 4 35043 Marburg Germany

**Keywords:** azirines, design of experiments, electrochemical oxidation, iodine, oxazole

## Abstract

An electrochemical iodine‐mediated transformation of enamino‐esters for the synthesis of 2*H*‐azirine‐2‐carboxylates is presented. In addition, a thermic conversion of azirines to 4‐carboxy‐oxazoles in quantitative yield without purification was described. Both classes 2*H*‐azirines‐2‐carboxylates and the 4‐carboxy‐oxazoles are substructures in natural products and therefore are of considerable interest for synthetic and pharmaceutical chemists. The optimization was not performed in a conventional manner with a one‐factor‐at‐a‐time process but with a *Design of Experiments* (*DoE*) approach. Beside a broad substrate scope the reaction was also employed to a robustness screen, a sensitivity assessment, and complemented with mechanistic considerations from cyclic voltammetry experiments.

## Graphical abstract

**United we stand**—**divided we electrolyze**! The electrochemical oxidation of iodide anions to lutidine‐stabilized iodonium ions (lutidine⋅I^+^) in organic solvents allowed the mediated cyclisation of enamino carboxylates in moderate to good yields. The reaction conditions were optimized utilizing the *Design of Experiment* approach. The electrochemical reaction conditions were tested in a sensitivity assessment and a robustness screen was performed. Finally, the thermal treatment of the azirine products led to several oxazoles in quantitative yields.

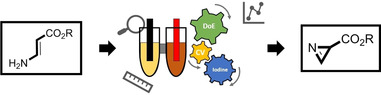

Azirines are rather unusual strained unsaturated three‐membered rings with a nitrogen atom where two types of isomers are possible: 1*H*‐azirines, containing a carbon‐carbon double bond that are not stable and isomerize to 2*H*‐azirines containing an imine subunit. One special class of azirines are the 2*H*‐azirine‐2‐carboxylates (marked in blue, Figure 1), which occur in natural products such as *dysidazirine*, and *antazirine* from the *Dysidea fragilis* sponge,^1^ as well as *azirinomycin* from *Streptomyces aureus*.^2^

**Figure 1:**
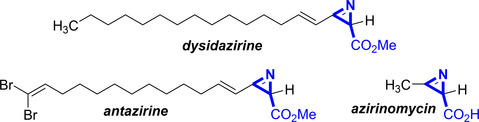
Natural products with 2*H*‐azirine‐2‐carboxylate moieties.

The common synthesis of azirines can be achieved in several ways like the usage of azides to form nitrenes under thermal conditions,^3^ addition of nitrenes to alkynes or addition of carbenes on nitriles,^4^ ring contraction of isoxazoles,^5^ elimination reactions from oxime derivatives,^6^ or by strong oxidants like PIDA or PIFA from enamines.^7^, ^8^, ^9^, ^10^ Unfortunately, the use of mild oxidants, such as iodine^11^, ^12^, ^13^ for the oxidation of enamines is rare and in all cases over stoichiometric amounts of oxidants are needed. Hazardous reagents like azides, strong bases, strong oxidants, or high temperatures as well as a disadvantageous atom economy are great drawbacks of these reactions; therefore, a simple conversion with a mild oxidant in catalytic amounts under mild conditions would overcome most of the drawbacks of the described synthetic methods (Scheme 1). In initial experiments based on previous work, we discovered that under electrochemical conditions an enamino ester, such as **1 a**, was converted into the azirine **2 a** in moderate yield (19 %) when iodine was present in the anodic cell compartment of a divided electrolysis under galvanostatic conditions. Intrigued by this finding, we decided to investigate this electrochemical cyclization reaction towards such a strained unsaturated heterocycle in more detail.

**Scheme 1:**
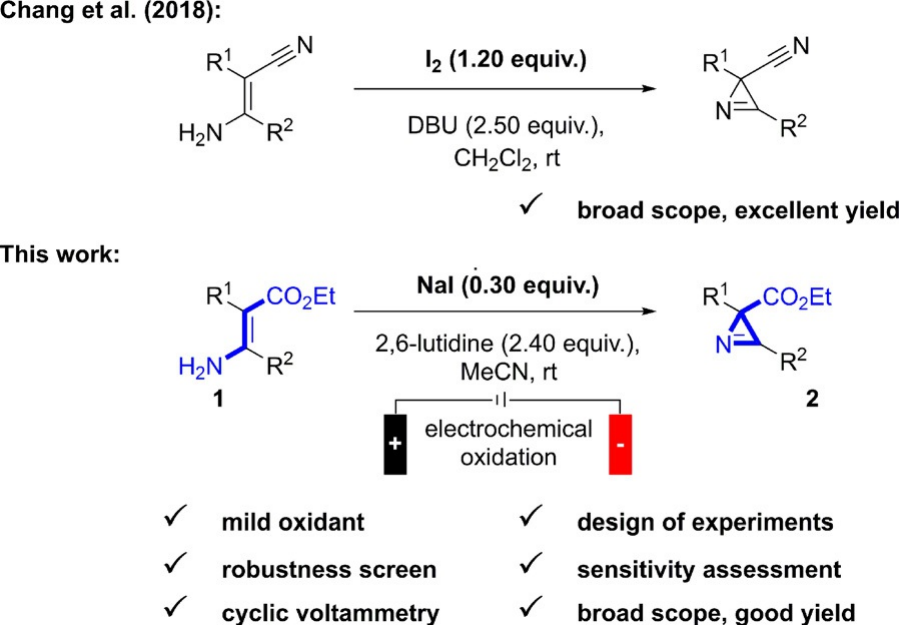
Electrochemical cyclization of enamino ester **1** to the azirine **2**.

Nowadays, electrochemical processes are favorable compared to chemical oxidants and reductants, because less waste is accumulated beside the usage of supporting electrolyte and hazardous reagents can be avoided; in addition to economic reasons.^14^ The main challenge of electrochemical methods are the additional parameters (e.g. electrode material, cell design, supporting electrolyte),^15^ which need to be optimized on top of the reaction conditions of an ordinary organic reaction (e.g. temperature, concentration, reaction time). The additional parameters aggravate the optimization, that is why we decided to utilize the *design of experiments* (*DoE*) approach^16^, ^17^ where a large number of parameters can be optimized at once very effectively and with higher statistical significance by running a small set of experiments. The aim of this optimization procedure is to perform a small set of experiments, where all parameters are changed at the same time in a systematic order. The interaction of each parameter among themselves and the influence on the yield are described mathematically by using *DoE*. The outcome of this analysis is statistically proven and the influence of each parameter can be quantified (Figure 2). Cross interactions, outliers due to experimental errors, and the optimal reaction conditions can easily be found. In earlier work we already demonstrated that *DoE* in combination with electrochemical transformations can be a valuable tool to improve the reaction performance significantly.^18^, ^19^ For the screening process, we choose **1 b** (ethyl (*E*)‐2‐(amino(4‐fluorophenyl)methylene)‐3‐oxobutanoate; compare Scheme 2) as the test substrate in order to determine yields by ^19^F NMR spectroscopy directly from the electrolyte because the product **2 b** decomposes under the thermal conditions of gas chromatographic analysis. Initially in the *DoE* optimization, we started to investigate categorical parameters in a rough extensive screening. It seems that the optimization of categorical parameters very often relied on accidental discoveries of trial and error experiments because they have a non‐linear correlation. For systematic optimization of categorical parameters in an one‐factor‐at‐a‐time process, it is necessary to cover the whole reaction space by testing all possible combinations, which would lead to extensive experimental efforts. The selection of the categorical parameters and the limits of the numerical parameters were made especially with respect to mild and less hazardous reaction conditions avoiding large excesses of additives or electric current to be applied. The following categorical parameters were investigated and for the solvents (MeOH, DMF, **MeCN**), the iodide source (Bu_4_NI, **NaI**, KI, PhI), the base (pyridine, DBU, KOPiv, **2,6‐lutidine**), the supporting electrolyte (NBu_4_BF_4_, LiClO_4_, **NEt_4_OTs**), the anode material (**graphite**, platinum, glassy carbon), and the cathode material (graphite, **platinum**, glassy carbon) the bold formatted parameters were identified to be optimal in terms of the yield. The screening of the categorical parameters required 65 experiments by using *DoE* instead of 1296 experiments (4⋅4⋅3⋅3⋅3⋅3) for coverage of the complete reaction space (see the Supporting Information).

**Figure 2:**
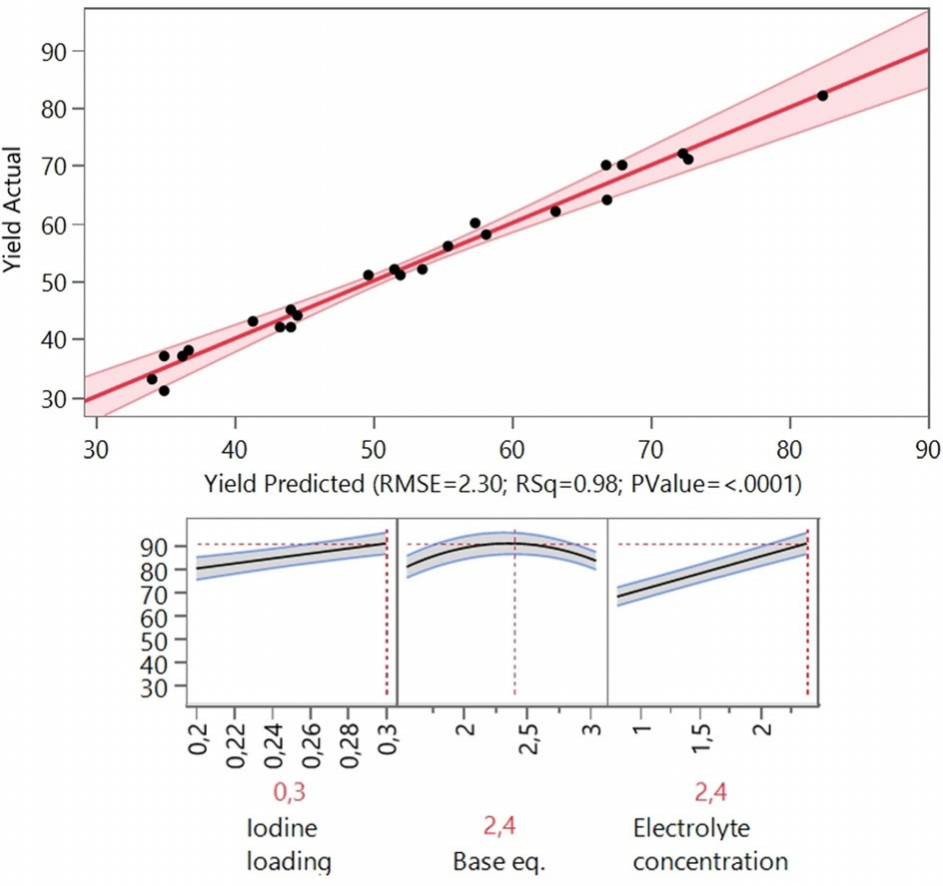
Reaction optimization using *Design of Experiments*. Predicted yields are plotted versus measured yields (in total 25 experiments: 21 for the optimization, 4 for replication of different experiments). All reactions were carried out on a 0.5 mmol scale using substrate **2 b**. Yields were determined by ^19^F NMR spectroscopy using 2‐nitro‐fluorobenzene as the internal standard.

**Scheme 2:**
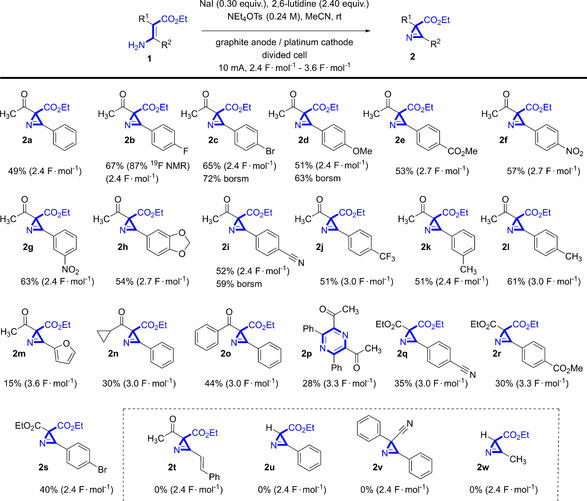
Substrate scope of the electrochemical iodine‐mediated oxidation of enamines to azirines. ^19^F NMR yields (2‐nitro‐fluorobenzene as internal standard) and the applied currents are given in parenthesis. Some yields are given additionally as based on recovered starting material (borsm) yields.

With these best categorical parameters, we started with a d‐optimal optimization design which has been extended for the coverage of cross interactions. We needed 25 experiments for the numerical parameters containing mediator loading (0.20–0.30 equiv., optimal: 0.30 equiv.), 2,6‐lutidine equivalents (1.00–3.00, optimal: 2.40), electrolyte concentration (0.08–0.24 mol L^−1^, optimal: 0.24 mol L^−1^), temperature (25–55 °C, optimal: 25 °C), applied charge (1.8–2.4 F mol^−1^, optimal: 2.4 F mol^−1^) and charge density (6–10 mA, optimal: 10 mA). The predicted yield by using *DoE* is correlated with the actual yield (Figure 2) showing a very good representation of the influence between the numerical parameters and the yield. Some of the numerical parameters are at the limits of the prior defined range. We didn't want to extend the range to keep the reaction conditions mild, the experimental set up easy, and avoid large excess of additives. The resulting model identified 10 relevant interactions (see The Supporting Information) where a quadratic term of the base equivalents had the greatest significance besides the electrolyte concentration and a cross interaction between temperature and electric current was also relevant (all *p*‐values<0.01); thus, resulting in a high R^2^ value of 0.98. After the initial optimization by the *DoE* approach, the assessment of systematic and/or random errors^20^ was investigated to increase the reproducibility of the electrochemical methodology, which has hitherto only been performed in photochemical reactions.^21^, ^22^ Therefore, the reaction was tested with the sensitivity assessment and examined beside typical parameters like concentration or moisture content, also parameters unique for electrochemical conversions, such as the distance between the electrodes and the surface area of the electrodes (Figure 3). Fortunately, the electrochemical reaction proved to be very insensitive to systematic or random errors so that the reaction should be reproducible also for other researchers even if small deviations of the reaction conditions are applied.

**Figure 3:**
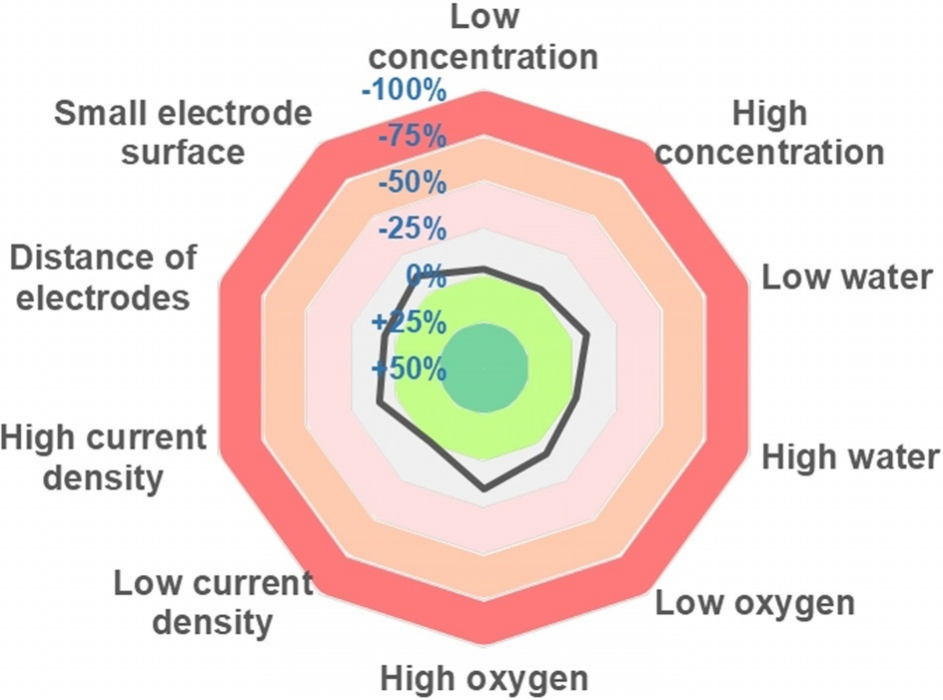
Sensitivity assessment. See the Supporting Information for more details.

After completion of the optimization and sensitivity assessment, we examined the scope and limitations of the electrochemical cyclization reaction towards azirines (Scheme 2). Unfortunately, the isolated yields differed from those determined by ^19^F NMR (67 % compared to 87 %). Therefore, we performed an intensive analysis of all individual work‐up steps and evaluated how much product was lost in every work‐up step (see SI). Since the azirine moiety is very labile the highest isolated yield was obtained when extraction with aqueous saturated NH_4_Cl solution as well as flash chromatography on neutral silica gel was performed. However, independent from the work‐up procedure, loss of a fraction of the product could not be avoided.

The isolated yields of the azirines of type **2**, obtained from the electrochemical reaction, are quite acceptable and range for a considerable large number of products between 40–67 %. The scope of the reaction with respect to the substituent R^2^ ranges from electron‐deficient to electron‐rich arene moieties which are both tolerated with similar results. The reaction is limited to aromatic substituents as R^2^ which is in line with the previously reported chemical reaction conditions, where a new hypervalent iodine (III/V) oxidant was used to prepare azirines from enaminoesters.^23^ The position of the substituent on the arene ring in R^2^; either in *meta* or *para* position (substrates **2 f**/**2 g** and **2 k**/**2 l**) seems to have a negligible effect upon the yield of the reaction. However, a substrate of type **1** with a substituent in *ortho* position was not accessible. The scope of the substituent R^1^ ranged from simple methyl β‐keto esters (**2 a**–**2 m**) over cyclopropyl (**2 n**) and phenyl (**2 o**) substituted carbonyl compounds and malonates (**2 q**–**2 s**). As expected, electron‐poor substrates gave better yields: accordingly, the nitrile **2 i**, products **2 f**/**2 g** with nitro substituents and ester **2 e** could be isolated in good yields. We expected that the yield of product **2 d** and **2 h** would be lower, since electrochemical direct iodination could occur,^24^ but in both cases such side products were not observed. Further functional groups like halides were investigated (fluoride, bromide, CF_3_) and the corresponding products were isolated in moderate to good yields. Interestingly, the substrate **1 p** (4‐amino‐4‐phenylbut‐3‐en‐2‐one) led to a different product and the analytical data revealed that the dimer (**2 p**) was formed in 28 % yield. The reaction was not successful when an additional double bond in conjugation to the enamine was present (**2 t**). Likewise, the reaction was not applicable when a trisubstituted enamine was utilized (compounds **2 u** and **2 w**, R^1^=H). Unfortunately, compound **2 v** does not react under electrochemical conditions, while a conventional method with iodine was successful.^11^ We reasoned that the oxidation potential of the enamine or the corresponding azirine must be lower than iodine, therefore we tried an ex‐cell approach where we electrolyzed 1.20 equiv of sodium iodide under equal conditions without enamine for 2.4 F mol^−1^ and then added the enamine to the solution without applying further charge; surprisingly, still no formation of the azirine was observed.

Since many substrates of type **1** are difficult to synthesize^25^, ^26^ and it is challenging to screen a large amount of functional groups very effectively, we applied a robustness screen^27^, ^28^ upon the electrochemical transformation of enamino esters to azirines (Table 1, see The Supporting Information for more examples). The reaction proceeds in good yields in most cases even though additives are present. Some additives are stable under the conditions (entries 1, 6 and 13), while primary amines (entry 7), sulfolane (entry 8), or amides (entry 12) were not tolerated. *N*‐Methyl indole (entry 11) inhibit the reaction, because iodination of the indole occurs as shown via GC‐MS analysis (similar outcome for *N*‐benzyl pyrrole, see SI for complete table). Surprisingly alkenes and alkynes (entries 4 and 9) were tolerated in moderate yields, although iodination of the unsaturated units could occur but was not observed. However, the presence of an additional conjugated double bond as in substrate **2 t** was not tolerated under the electrochemical reaction conditions. A reason for the relatively high functional group tolerance could be that the reactive iodine species is generated continuously in low concentration in situ, which could be the decisive advantage of electrochemical methods over conventional synthesis in this case.

**Table 1:** Functional‐group tolerance test utilizing screening substrate **1 b**.^[a]^

	Additive	Yield [%] **1 b** ^[b]^	Yield [%] **2 b** ^[b]^	Yield [%] additive^[c]^
	**no additive**	**0**	**87**	**–**
**1**	2‐chlorochinoline	3	**69**	**>95**
**2**	benzaldehyde	2	**73**	**49**
**3**	phenole	78	**1**	**39**
**4**	1‐dodecyne	10	**70**	**56**
**5**	acetanilide	0	**53**	**32**
**6**	1‐chlorooctane	2	**69**	**>95**
**7**	dodecylamine	15	**72**	**3**
**8**	sulfolane	3	**70**	**0**
**9**	1‐octene	17	**57**	**55**
**10**	benzothiazole	0	**67**	**58**
**11**	*N‐*methyl indole	89	**1**	**0**
**12**	Boc‐piperidone	2	**72**	**0**
**13**	benzonitrile	2	**73**	**67**
**14**	1,2‐epoxyoctane	10	**76**	**61**

[a] All reactions were carried out on a 0.5 mmol scale. [b] Yields were determined by ^19^F NMR spectroscopy using 2‐nitro‐fluorobenzene as the internal standard. [c] Yields were determined by GC‐FID analysis using *n*‐dodecane as the internal standard. More examples are given in the Supporting Information.

For mechanistic considerations cyclic voltammetry experiments were performed. Yu and Chang^11^ postulated a nucleophilic attack from the enamine on iodine (I_2_) to transfer I^+^ onto the substrate and liberation of iodide anions (I^−^). Our initial hypothesis was that iodide was oxidized electrochemically to molecular iodine and then undergoes the desired reaction. However, direct oxidation of iodide occurs only in aqueous media and it is described that oxidation of iodide in organic solvents can be more complex.^29^ To verify that I_2_ was generated electrochemically under these reaction conditions, we performed the reaction in a flask under the same reaction conditions except replacing NaI by 1.2 equivalents of I_2_ and without applying electrical current to the reaction; the product was formed only in trace amounts. Even if the amount of I_2_ was increased up to 3.6 equivalents, only 25 % of product **2 a** was formed and 60 % of the starting material remaining unchanged. This result and the absence of any reaction with enamine nitrile **1 v** lead to the hypothesis that another oxidized iodine species might be involved. It is likely that iodide anions were oxidized electrochemically to form triiodide (I_3_^−^) in the first step (*E*_p_=0.75 V vs. Ag/AgCl) and another oxidation to molecular iodine occurred in a second step (*E_p_*=1.05 V vs. Ag/AgCl; Scheme 3).^30^, ^31^, ^32^

**Scheme 3:**
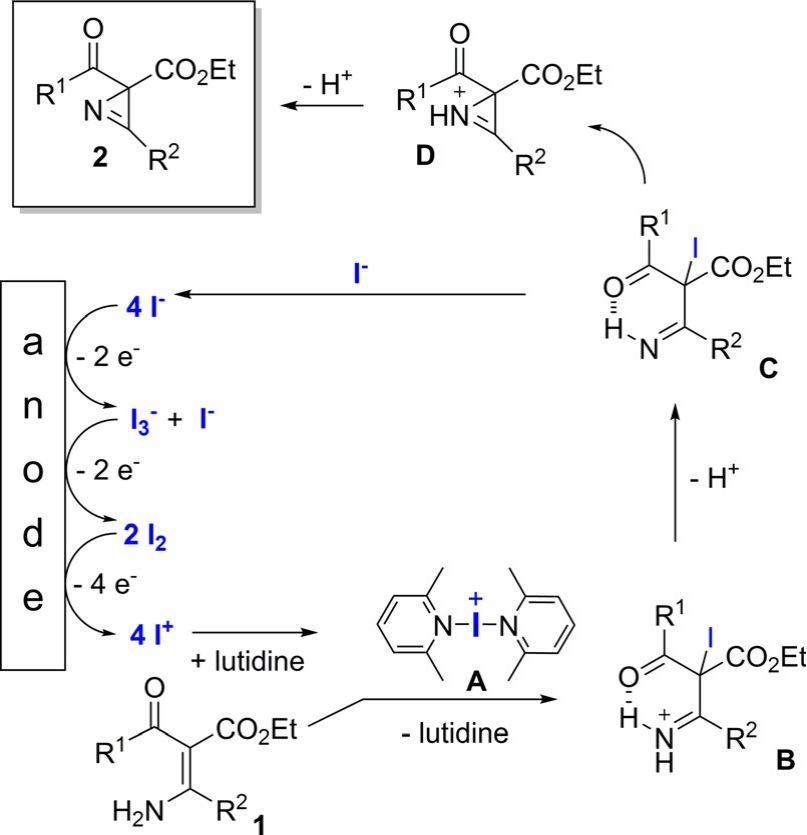
Postulated reaction mechanism.

To verify the formation of I^+^ we replaced the enamine carboxylate with 4‐tolyl trimethylsilane under equal conditions.^19^ The appearance of 4‐iodotoluene in GC‐MS analysis indicates the formation of I^+^ ions in the electrochemical reaction which underwent an *ipso*‐substitution reaction by iododesilylation. We assumed that under electrochemical conditions another oxidation of I_2_ to I^+^ or presumably a lutidine stabilized adduct (**A**) takes place. In the absence of 2,6‐lutidine the reaction of starting material **1 a** to **2 a** does not take place which could indicate that a complex of an iodine species and lutidine (or a lutidine‐acetonitrile complex) is involved to stabilize I^+^ in the reaction mixture (intermediate **A**, Scheme 3) or is necessary to deprotonate the intermediates **B** and **D**. In the absence of iodide, direct oxidation of enamine **1 a** does not lead to the desired product **2 a** despite full consumption of the enamine is observed. Oxidation of enamine **1 a** takes place at higher potentials (Figure 4, red curve) but under galvanostatic conditions NaI will be oxidized first. The cyclic voltammetry also showed that neither 2,6‐lutidine nor the azirine **2 a** or the supporting electrolyte were oxidized.

**Figure 4:**
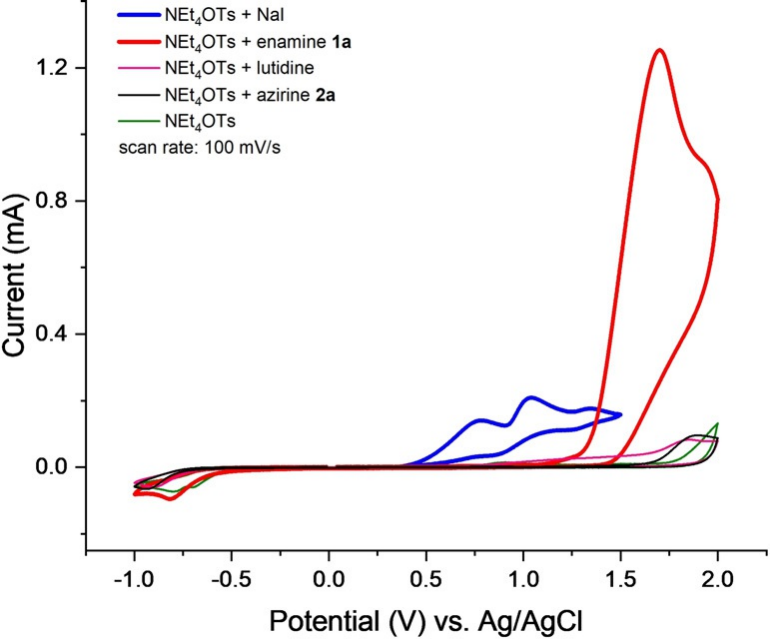
Cyclic voltammograms of NaI, enamine **1 a**, 2,6‐lutidine, azirine **2 a** and supporting electrolyte (NEt_4_OTs).

Analog complexes of pyridine ligands with I^+^ have been described and characterized by ^13^C NMR.^33^ The enamine carboxylate **1** then reacts with the formal I^+^‐reagent **A** to form intermediate **B** which is stabilized by a hydrogen bond with the adjacent carbonyl group. The stabilization of the intermediates **B** and **C** could rationalize why enamino keto esters react more readily than enamino diesters and enamino nitriles, while substrates such as **2 u** and **2 w** do not react. After deprotonation by 2,6‐lutidine towards **C**, the ring closure occurs to form intermediate **D** under liberation of iodide anions and another deprotonation generates the desired product **2** with a total number of 2 electrons consumed in this oxidation.

When we started the investigation of the electrochemical cyclization reaction of enamino esters, also another isomer with the identical molecular mass was detected by GC‐MS analysis while such an isomer was not detectable by ^19^F NMR analysis. Accordingly, a thermal rearrangement in the GC oven led to the formation of the isomer. Therefore, thermal treatment of the azirines of type **2** in a sealed tube at elevated temperatures led to the isomer detected by GC‐MS analysis. The product **3** could be isolated in all investigated examples (Scheme 4) in quantitative yield without any tedious work‐up or purification step; only removal of the solvent—like similar literature known rearrangements of azirines to 4‐carboxy‐oxazoles.^34^, ^35^ 4‐Carboxy‐oxazoles are a special class of oxazoles, which appear in natural products like *virginiamycin M_1_* or *bistratamide C*.^36^ Notably, starting material **2 d** with an electron‐poor aryl substituent reacted slow compared to the other azirines.

**Scheme 4:**
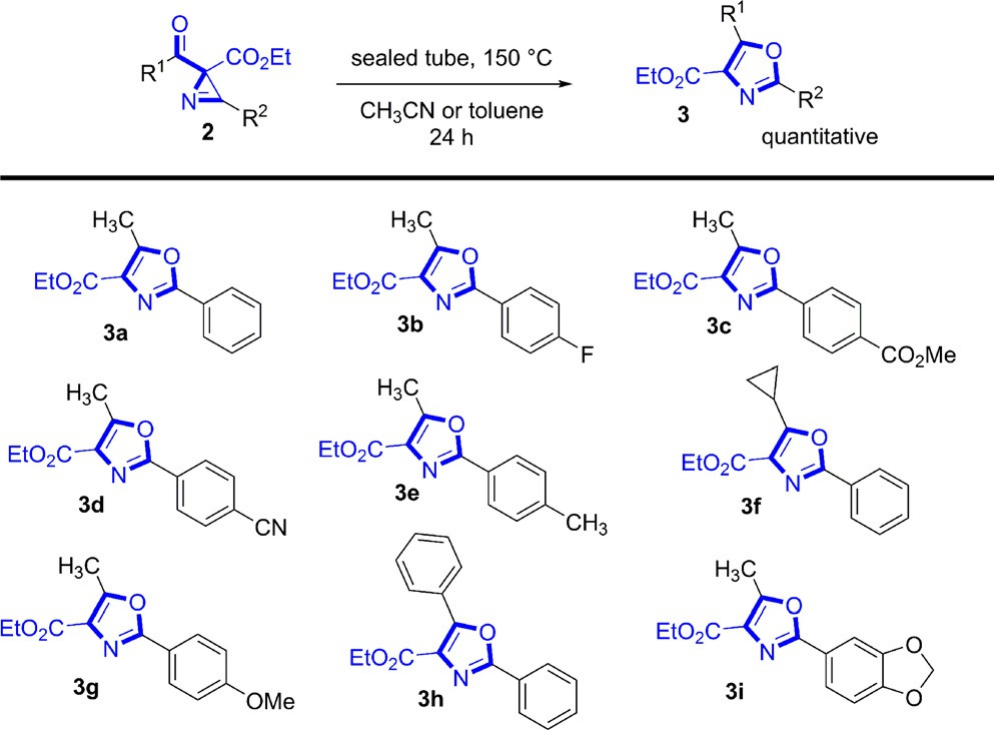
Thermal rearrangement of azirines to 4‐carboxy‐oxazoles. In case of substrate **2 d** 120 h reaction time.

In conclusion, we realized a new electrochemical iodine‐mediated synthesis of 2*H*‐azirine‐2‐carboxylates and their follow up reaction to 4‐carboxy‐oxazoles, which are both interesting substructures of natural products. To reduce the number of experiments and at the same time increase statistical significance of the optimization, we performed the reaction optimization with the *Design of Experiments* approach. We demonstrated the applicability of this method by a broad substrate scope and a wide robustness screen; above all, we performed a sensitivity assessment in an electrochemical reaction. Also, cyclic voltammetry experiments were conducted to get mechanistic insights of the reaction. Further investigations, targeting the electrochemical synthesis of the above‐mentioned natural products are under current investigation.

## Conflict of interest

The authors declare no conflict of interest.

## Supporting information

As a service to our authors and readers, this journal provides supporting information supplied by the authors. Such materials are peer reviewed and may be re‐organized for online delivery, but are not copy‐edited or typeset. Technical support issues arising from supporting information (other than missing files) should be addressed to the authors.

Supplementary
