# Supplementary material for: Electrochemical Iodine‐Mediated Oxidation of Enamino‐Esters to 2H‐Azirine‐2‐Carboxylates Supported by Design of Experiments
Source: Chemistry. 2020 Jul 14;26(41):8879–84. doi: 10.1002/chem.202001465 (PMC7497194; doi:10.1002/chem.202001465)
Supplement: Supplementary file 1 — Supplementary [file CHEM-26-8879-s001.pdf]

Chemistry–A European Journal

Supporting Information

Electrochemical Iodine-Mediated Oxidation of Enamino-Esters to 2*H*-Azirine-2-Carboxylates Supported by *Design of Experiments*

Emre Babaoglu[a, b] and Gerhard Hilt*[a]

Table of Content

Experimental procedures 2
Determining the influence of supporting electrolyte hydrolysis 4
Determining the influence of work up procedure 5
General procedure for the electrochemical synthesis of 2*H*-azirines-2-carboxylates 8
General procedure for the synthesis of 4-carboxy-oxazoles 18
General procedure for the synthesis of enamines 23
Reaction optimization by *Design of Experiments* 31
Sensitivity assessment 44
Robustness screen 47
Cyclic voltammetry 49
NMR spectra 60
References 87

Experimental procedures

All experiments were performed under standard Schlenk techniques using heat gun dried glassware under argon atmosphere. Yields were determined gravimetrically or in case of analytical yields under conventional methods per GC-FID or ^{19}F NMR with internal standards. GC-FID spectra were measured on a *Shimadzu GC-2010 Plus* spectrometer with an *Optima 5 MS Macherey Nagel* column (15 m length, diameter 0.25 mm, 0.25 μm film thickness), temperature profile: 50 °C, hold 3 min, increment 40 °C·min^{-1}, final temperature 280 °C, hold 4 min. Yields were determined using *n*-dodecane as internal standard. For preparation of the GC samples 0.1 mL of the reaction solution was filtered through a short plug of silica gel into a vial, rinsing with MTBE thoroughly.

GC-MS spectra were recorded utilizing a *Shimadzu QP2020* spectrometer. Ionisation was accomplished by electron ionisation (EI) at an energy of 70 eV. Reagents were purchased from commercial sources and were used without further purification. Non absolute solvents for flash chromatography or extraction were purchased in technical grade and distilled prior to use.

NMR were recorded on *Bruker Avance 300, Bruker Avance 500, and Bruker Avance III 500* spectrometers. Chemical shifts were reported in parts per million (ppm), multiplicity was reported as br s = broad singlet, s = singlet, d = doublet, t = triplet, q = quartet, m = multiplet, coupling constants were reported in Hz. ^{1}H, ^{13}C were recorded in $CDCl_3$ as reference (δ = 7.26 ppm for ^{1}H NMR, δ = 77.2 ppm for ^{13}C NMR). ^{19}F NMR for determination of analytical yields were recorded in the respective reaction solvent with *2*-nitro-fluorobenzene (δ = -119.3 ppm in MeCN) as internal standard using *Eppendorf™* pipettes or by using stock solutions with a fused glass capillary containing D_2O in the NMR tube for locking. ^{19}F NMR were measured in the range of -40 ppm to -190 ppm with middle of spectrum set as -115 ppm. Spectra were manually phase corrected and a multipoint baseline correction was applied.

Flash chromatography (FC) was carried out on silica gel 60 (40-64 µm, 230-400 mesh ASTM) purchased from either *Merck* or *Macherey-Nagel* with solvent in technical grade, which were distilled prior to use.

Design of Experiments was performed using *JMP 13* software package by SAS (version *13.2.1*. SAS Institute Inc., Cary, NC, 2016). Electrochemical reactions were performed under non-inert conditions using HPLC solvents in a H-type electrolysis cell (Figure **1**) using *AIM-TTI Instruments MX100T* as power supply.

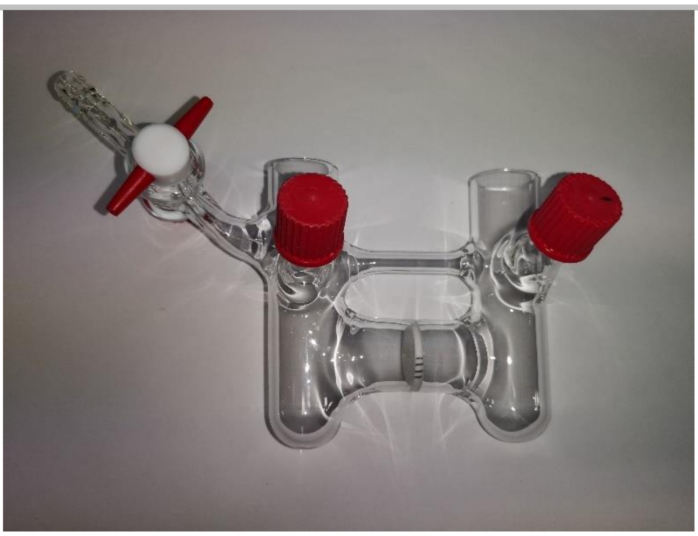

Figure 1: H-Type cell applied for the electrochemical reactions.

Electrode dimensions are 1.7 x 3.0 cm (Figure **2**). The distance between the electrodes is 6.5 cm.

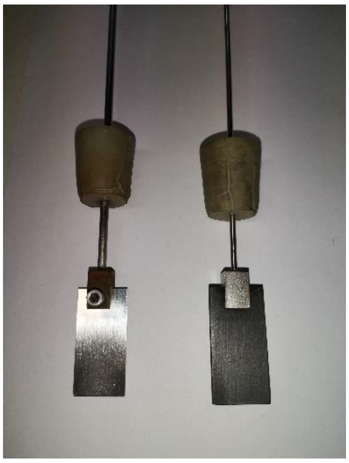

Figure 2: Platinum and graphite electrodes with holder.

IR spectra were recorded on a Bruker Tensor 27 spectrometer equipped with a GoldenGate diamond ATR unit or on a Shimadzu IRSpirit QATR-S.

Determining the influence of supporting electrolyte hydrolysis

The supporting electrolyte is hygroscopic, which already shows up when weighting. To see the influence of partially hydrolysis of the supporting electrolyte we applied the hydrolysis products to the reaction. Triethylamine and *p*-toluene sulfonic acid (monohydrate) as hydrolysis products were given into the anodic and cathodic compartments and then the reaction was performed as always.

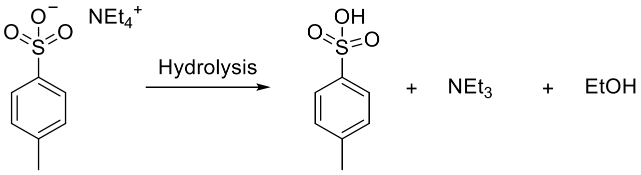

In a H-type cell, both compartments were charged with NEt_4OTs (723 mg, 4.80 eq., 2.40 mmol) and a hydrolysis product (0.50 eq., 0.25 mmol, each compartment) in acetonitrile (each compartment 10 mL). Sodium iodide (22.4 mg, 0.30 eq., 0.15 mmol), 2,6-lutidine (130 mg, 2.40 eq., 1.20 mmol) and enamine **1b** (126 mg, 1.00 eq., 0.50 mmol) were added to the anode compartment. The cathode compartment was equipped with platinum electrode, the anode compartment was equipped with graphite electrode. The reaction was electrolyzed under constant current (10 $mA \cdot cm^{-2}$) until 2.4 $F \cdot mol^{-1}$ was applied. Yields were determined by ^{19}F NMR using *2*-nitro-fluorobenzene (52.6 μL, 1.00 eq., 0.50 mmol) as internal standard.

Table 1: Determining the influence of adding 0.50 eq. of the supporting electrolytes hydrolysis product to the reaction of enamine **1b.**

	**Hydrolysis product**	**Yield starting material**	**Yield product**
Ref.	–	**0%**	**87%**
**1**	Triethylamine	51%	**31%**
**2**	*p*-Toluene sulfonic acid (monohydrate)	0%	**65%**

Obviously, triethylamine inhibited the reaction, because significant amounts of the starting material were not converted. Also, the addition of *p*-toluene sulfonic acid (monohydrate) reduced the yield. Accordingly, the supporting electrolyte was stored under argon atmosphere to prevent hydrolysis.

Determining the influence of work up procedure

In a H-type cell, both compartments were charged with NEt_4OTs (1.45 g, 4.80 eq., 4.80 mmol) in acetonitrile (each compartment 20 mL). Sodium iodide (44.8 mg, 0.30 eq., 0.30 mmol), 2,6-lutidine (260 mg, 2.40 eq., 2.40 mmol) and enamine **1b** (252 mg, 1.00 eq., 1.00 mmol) were added to the anode compartment, equipped with a platinum cathode and a graphite anode. The reaction was electrolyzed under constant current (10 $mA\cdot cm^{-2}$) until 2.4 $F\cdot mol^{-1}$ was applied.
The anode compartment was poured into a volumetric flask and filled up to 50 mL with acetonitrile before aliquots (5 mL) were taken for a reference measurement and for each work up step.

For filter column:

The crude product (5 mL) was filtered through a short plug of silica gel or basic alumina (eluent: ethyl acetate 75 mL).

For extraction:

The crude product (5 mL) was poured into 20 mL of the respective aqueous solution (NaOH 3 $mol\cdot L^{-1}$, water, sat. $Na_2S_2O_3$, sat. NH_4Cl, or HCl 3 $mol\cdot L^{-1}$) and extracted with ethyl acetate (3 × 20 mL).

After the workup, to each sample the internal standard *2*-nitro-fluorobenzene (52.6 μL, 1.00 eq., 0.50 mmol) was added and filled in a NMR tube with a D_2O capillary to determine the yield. The yields were given in absolute yields (compared to the integral of 1.00 eq. of 2-nitro-fluorobenzene in ^{19}F NMR) and relative to the reference (79% relative intensity).

We chose extraction with saturated NH_4Cl-solution (Table **2**) as optimal work up procedure, because less side products are formed. Entry 2 demonstrates that flash chromatography on silica gel causes loss of product as well. This analysis indicates that almost 20 % of the product is lost during the extraction and chromatography.

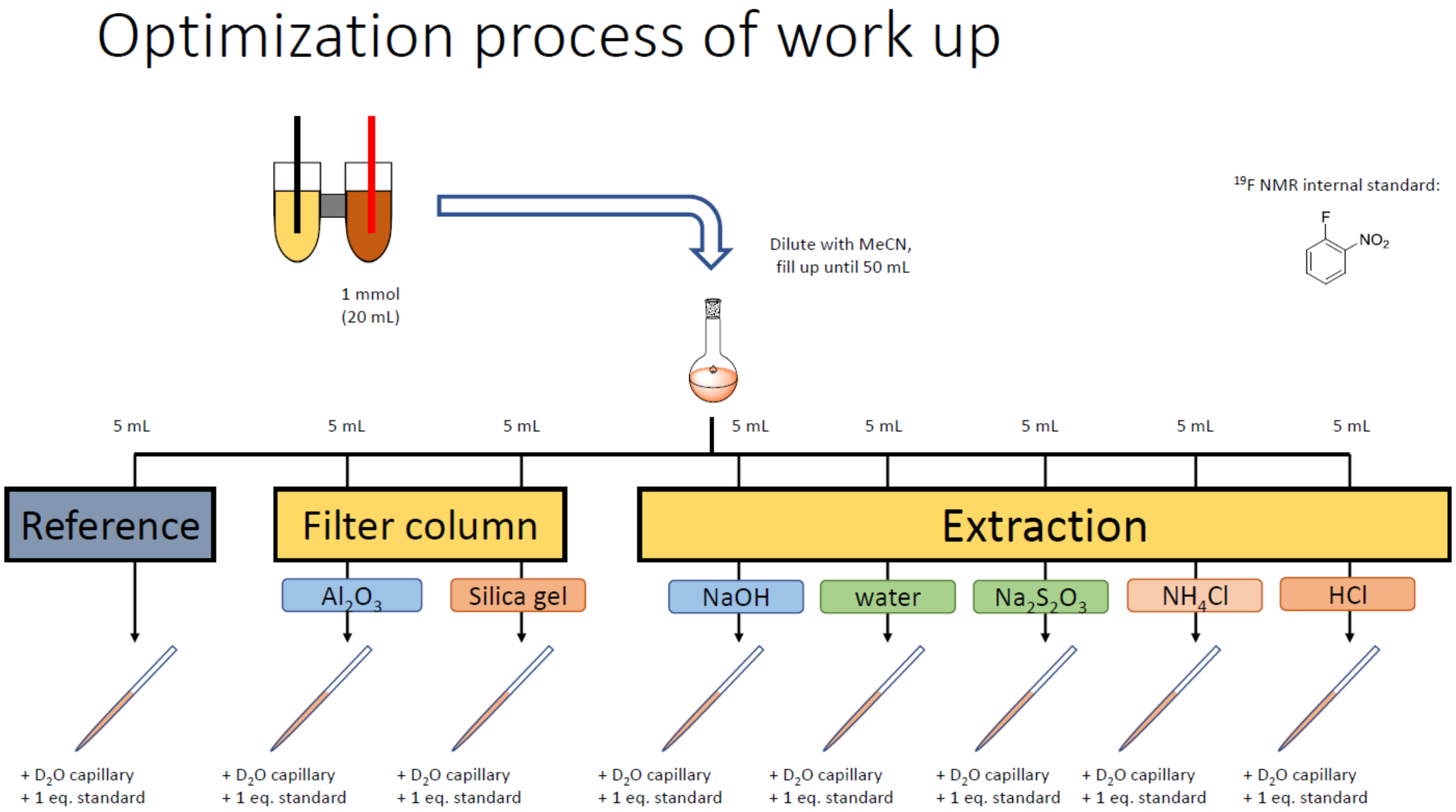

Figure 3: Work up process.

Table 2: Yields and relative yields (compared to the reference) for every work up step.

		Yield	Yield side product	Relative yield
	**Reference**	79%	8%	-
1	Al_2O_3 (basic)	70%	6%	89%
2	Silica gel	71%	5%	90%
3	NaOH (3 $mol{\cdot}L^{-1}$)	41%	8%	52%
4	Water	70%	0%	89%
5	$Na_2S_2O_3$ (saturated)	72%	4%	91%
6	**NH_4Cl (saturated)**	**72%**	**1%**	**91%**
7	HCl (3 $mol{\cdot}L^{-1}$)	62%	7%	78%

General procedure for the electrochemical synthesis of 2*H*-azirines-2-carboxylates

In a H-type cell, both cell compartments were equipped with NEt_4OTs (723 mg, 4.80 eq., 2.40 mmol) in acetonitrile (each compartment 10 mL). Sodium iodide (22.4 mg, 0.30 eq., 0.15 mmol), 2,6-lutidine (130 mg, 2.40 eq., 1.20 mmol) and the respective enamine (1.00 eq., 0.50 mmol) were added to the anode compartment. The cathode compartment was equipped with a platinum electrode, the anode compartment was equipped with a graphite electrode. The reaction was electrolyzed under constant current (10 $mA \cdot cm^{-2}$) at room temperature until complete conversion (TLC analysis) was detected. The anode compartment solution was diluted with saturated aqueous NH_4Cl solution and extracted with ethyl acetate (3 × 25 mL), dried over $MgSO_4$ and concentrated under reduced pressure. The crude product was purified by flash chromatography over silica gel.

Ethyl 2-acetyl-3-phenyl-2*H*-azirine-2-carboxylate (1a)

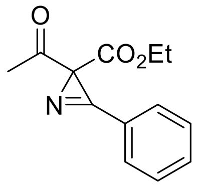

Ethyl 2-(amino(phenyl)methylene)-3-oxobutanoate (117 mg, 0.50 mmol, 1.00 eq.) was reacted according to the general procedure until 2.4 $F \cdot mol^{-1}$ was applied. The pure product was obtained as yellowish oil after flash chromatography over silica gel (*n*-pentane:ethyl acetate = 5:1).

Yield: 49% (57.0 mg, 0.25 mmol).

$\mathbf{R_f}$ = 0.38 (*n*-pentane:ethyl acetate = 5:1).

^{1}H NMR (300 MHz, $CDCl_3$): δ = 7.85 (dt, *J* = 8.2, 1.3 Hz, 2H), 7.68 – 7.62 (m, 1H), 7.56 (td, *J* = 7.3, 1.2 Hz, 2H), 4.20 (q, *J* = 7.1 Hz, 2H), 2.40 (s, 3H), 1.22 (t, *J* = 7.1 Hz, 3H) ppm.

^{13}C NMR (75 MHz, $CDCl_3$): δ = 201.6, 168.2, 154.8, 134.6, 131.0 (2C), 129.6 (2C), 120.4, 61.9, 45.1, 28.9, 14.1 ppm.

IR (ATR): $\tilde{\nu}$ = 603 (w), 687 (m), 762 (m), 1022 (m), 1047 (m), 1132 (m), 1217 (m), 1282 (m), 1360 (m), 1450 (m), 1473 (m), 1700 (m), 1724 (s), 2987 (w) cm^{-1}.

HRMS (EI): m/z calculated for $C_{13}H_{13}O_3N$ ($[M]^+$): 231.0890; found: 231.0884.

Ethyl 2-acetyl-3-(4-fluorophenyl)-2*H*-azirine-2-carboxylate (2b)

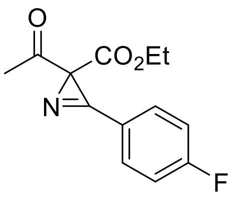

Ethyl 2-(amino(4-fluorophenyl)methylene)-3-oxobutanoate (126 mg, 0.50 mmol, 1.00 eq.) was reacted according to the general procedure until 2.4 F·mol^{-1} was applied. The pure product was obtained as colorless oil after flash chromatography over silica gel (*n*-pentane:ethyl acetate = 6:1).

Yield: 67% (84.0 mg, 0.34 mmol).

R$_f$ = 0.38 (*n*-pentane:ethyl acetate = 5:1).

^{1}H NMR (300 MHz, $CDCl_3$): *δ* = 7.98 – 7.65 (m, 2H), 7.44 – 7.03 (m, 2H), 4.16 (q, *J* = 7.1 Hz, 2H), 2.36 (s, 3H), 1.18 (t, *J* = 7.1 Hz, 3H) ppm.

^{13}C NMR (75 MHz, $CDCl_3$): *δ* = 201.7, 168.2, 164.7, 153.9, 133.6 (d, *J* = 9.7 Hz, 2C), 117.3 (d, *J* = 22.6 Hz, 2C), 116.8 (d, J = 3.2 Hz), 62.1, 45.2, 29.0, 14.2 ppm.

^{19}F NMR (470 MHz, $CDCl_3$) δ = -101.1 ppm.

IR (ATR): $\tilde{\nu}$ = 597 (m), 842 (s), 1047 (m), 1227 (m), 1282 (m), 1504 (s), 1599 (m), 1700 (s), 1724 (s), 2984 (w) cm^{-1}.

HRMS (EI): m/z calculated for $C_{13}H_{12}O_3NF$ ([M]$^+$): 249.0796; found: 249.0788.

Ethyl 2-acetyl-3-(4-bromophenyl)-2*H*-azirine-2-carboxylate (2c)

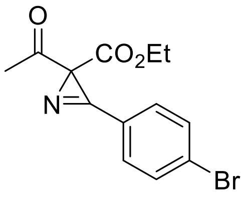

Ethyl 2-(amino(4-bromophenyl)methylene)-3-oxobutanoate (156 mg, 0.50 mmol, 1.00 eq.) was reacted according to the general procedure until 2.4 F·mol^{-1} was applied. The pure product was obtained as colorless oil after flash chromatography over silica gel (*n*-pentane:ethyl acetate = 5:1 → 3:1). Remaining educt could be re-isolated (19.0 mg, 0.06 mmol).

Yield: 65% (100 mg, 0.32 mmol), 72% borsm.

R$_f$ = 0.35 (*n*-pentane:ethyl acetate = 5:1).

^{1}H NMR (300 MHz, $CDCl_3$): *δ* = 7.71 (s, 4H), 4.19 (q, *J* = 7.1 Hz, 2H), 2.41 (s, 3H), 1.21 (t, *J* = 7.1 Hz, 3H) ppm.

^{13}C NMR (75 MHz, $CDCl_3$): *δ* = 201.5, 168.0, 154.4, 133.1 (2C), 132.1 (2C), 129.8, 119.3, 62.0, 45.1, 28.9, 14.1 ppm.

IR (ATR): $\tilde{\nu}$ = 562 (m), 832 (s), 1011 (s), 1053 (s), 1220 (s), 1287 (s), 1586 (m), 1694 (s), 1717 (s), 1788 (m), 1914 (w), 1980 (w), 1997 (w) cm^{-1}.

HRMS (EI): m/z calculated for $C_{13}H_{12}O_3NBr$ ([M]$^+$): 308.9995; found: 308.9992.

Ethyl 2-acetyl-3-(4-methoxyphenyl)-2*H*-azirine-2-carboxylate (2d)

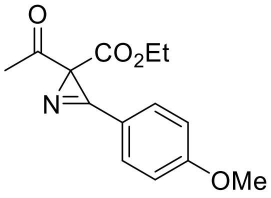

Ethyl 2-(amino(4-methoxyphenyl)methylene)-3-oxobutanoate (132 mg, 0.50 mmol, 1.00 eq.) was reacted according to the general procedure until 2.4 F·mol^{-1} was applied. The pure product was obtained as yellowish oil after flash chromatography over silica gel (*n*-pentane:ethyl acetate = 4:1 → 3:1). Remaining educt could be re-isolated (26.0 mg, 0.10 mmol).

Yield: 51% (66.0 mg, 0.25 mmol), 63% borsm.

$\mathbf{R_f}$ = 0.26 (*n*-pentane:ethyl acetate = 4:1).

^{1}H NMR (300 MHz, $CDCl_3$): δ = 7.80 (d, *J* = 8.8 Hz, 2H), 7.06 (d, *J* = 8.8 Hz, 2H), 4.20 (q, *J* = 7.1 Hz, 2H), 3.89 (s, 3H), 2.35 (s, 3H), 1.23 (t, *J* = 7.1 Hz, 3H) ppm.

^{13}C NMR (75 MHz, $CDCl_3$): *δ* = 202.1, 168.4, 164.7, 153.7, 133.3 (2C), 115.3 (2C), 112.4, 61.9, 55.8, 45.1, 28.7, 14.2 ppm.

IR (ATR): $\tilde{\nu}$ = 598 (m), 837 (s), 1020 (s), 1045 (m), 1170 (s), 1256 (s), 1507 (s), 1600 (s), 1697 (s), 1722 (s), 2939 (w), 2981 (w) cm^{-1}.

HRMS (EI): m/z calculated for $C_{14}H_{15}O_4N$ ([M]$^+$): 261.0996; found: 261.1000.

Ethyl 2-acetyl-3-(4-(methoxycarbonyl)phenyl)-2*H*-azirine-2-carboxylate (2e)

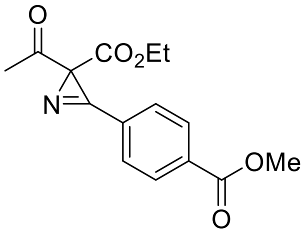

Ethyl 2-(amino(4-(methoxycarbonyl)phenyl)methylene)-3-oxobutanoate (146 mg, 0.50 mmol, 1.00 eq.) was reacted according to the general procedure until 2.7 F·mol^{-1} was applied. The pure product was obtained as colorless oil after flash chromatography over silica gel (*n*-pentane:ethyl acetate = 3:1).

Yield: 53% (76.0 mg, 0.26 mmol).

$\mathbf{R_f}$ = 0.31 (*n*-pentane:ethyl acetate = 3:1).

^{1}H NMR (300 MHz, $CDCl_3$): *δ* = 8.20 (d, *J* = 8.1 Hz, 2H), 7.92 (d, *J* = 8.1 Hz, 2H), 4.20 (q, *J* = 7.1 Hz, 2H), 3.94 (s, 3H), 2.44 (s, 3H), 1.21 (t, *J* = 7.1 Hz, 3H) ppm.

^{13}C NMR (75 MHz, $CDCl_3$): *δ* = 201.4, 167.9, 165.6, 154.7, 135.3, 130.8 (2C), 130.6 (2C), 124.2, 62.1, 52.8, 45.2, 29.1, 14.1 ppm.

IR (ATR): $\tilde{\nu}$ = 604 (m), 767 (m), 1054 (m), 1104 (m), 1221 (m), 1281 (s), 1691 (s), 1717 (s), 1791 (w), 2360 (w), 2953 (w), 2986 (w) cm^{-1}.

HRMS (EI): m/z calculated for $C_{15}H_{15}O_5N$ ([M]$^+$): 289.0945; found: 289.0945.

Ethyl 2-acetyl-3-(4-nitrophenyl)-2*H*-azirine-2-carboxylate (2f)

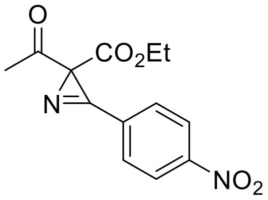

Ethyl 2-(amino(4-nitrophenyl)methylene)-3-oxobutanoate (139 mg, 0.50 mmol, 1.00 eq.) was reacted according to the general procedure until 2.7 F·mol^{-1} was applied. The pure product was obtained as brown oil after flash chromatography over silica gel (*n*-pentane:ethyl acetate = 5:1).

Yield: 57% (79.0 mg, 0.28 mmol).

R$_f$ = 0.28 (*n*-pentane:ethyl acetate = 5:1).

^{1}H NMR (300 MHz, $CDCl_3$): δ = 8.45 – 8.36 (m, 2H), 8.33 – 7.78 (m, 2H), 4.22 (q, *J* = 7.1 Hz, 2H), 2.51 (s, 3H), 1.23 (t, *J* = 7.1 Hz, 3H) ppm.

^{13}C NMR (75 MHz, $CDCl_3$): δ = 201.2, 167.6, 154.6, 151.1, 131.9 (2C), 126.2, 124.7 (2C), 62.4, 45.4, 29.2, 14.1 ppm.

IR (ATR): $\tilde{\nu}$ = 603 (m), 751 (m), 1059 (m), 1290 (s), 1346 (s), 1526 (s), 1690 (m), 1720 (s), 2360 (w), 2983 (w), 3072 (w), 3109 (w) cm^{-1}.

HRMS (EI): m/z calculated for $C_{13}H_{12}O_5N_2$ ($[M]^+$): 276.0741; found: 276.0730.

Ethyl 2-acetyl-3-(3-nitrophenyl)-2*H*-azirine-2-carboxylate (2g)

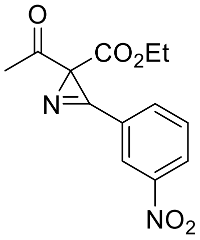

Ethyl 2-(amino(3-nitrophenyl)methylene)-3-oxobutanoate (139 mg, 0.50 mmol, 1.00 eq.) was reacted according to the general procedure until 2.4 F·mol^{-1} was applied. The pure product was obtained as colorless oil after flash chromatography over silica gel (*n*-pentane:ethyl acetate = 4:1 → 3:1).

Yield: 63% (87.0 mg, 0.31 mmol).

R$_f$ = 0.24 (*n*-pentane:ethyl acetate = 5:1).

^{1}H NMR (300 MHz, $CDCl_3$): δ = 8.64 (t, *J* = 1.9 Hz, 1H), 8.50 (ddd, *J* = 8.3, 2.3, 1.1 Hz, 1H), 8.21 (dt, *J* = 7.7, 1.3 Hz, 1H), 7.82 (t, *J* = 8.0 Hz, 1H), 4.23 (q, *J* = 7.3 Hz, 2H), 2.52 (s, 3H), 1.24 (t, *J* = 7.1 Hz, 3H) ppm.

^{13}C NMR (75 MHz, $CDCl_3$): δ = 201.3, 167.7, 154.4, 148.7, 136.1, 131.1, 128.7, 125.5, 122.5, 62.4, 45.4, 29.3, 14.1 ppm.

IR (ATR): $\tilde{\nu}$ = 601 (w), 676 (w), 733 (w), 1050 (w), 1287 (w), 1349 (w), 1532 (w), 1686 (w), 1723 (w), 1787 (w), 2337 (w), 2360 (w), 2983 (w), 3087 (w) cm^{-1}.

HRMS (EI): m/z calculated for $C_{13}H_{12}O_5N_2$ ($[M]^+$): 276.0741; found: 276.0743.

Ethyl 2-acetyl-3-(benzo[*d*][1,3]dioxol-5-yl)-2*H*-azirine-2-carboxylate (2h)

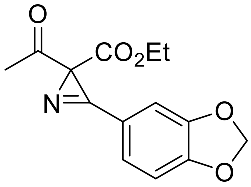

Ethyl 2-(amino(benzo[*d*][1,3]dioxol-5-yl)methylene)-3-oxobutanoate (139 mg, 0.50 mmol, 1.00 eq.) was reacted according to the general procedure until 2.7 F·mol^{-1} was applied. The pure product was obtained as yellowish oil after flash chromatography over silica gel (*n*-pentane:ethyl acetate = 3:1).

Yield: 54% (74.0 mg, 0.27 mmol).

$\mathbf{R_f}$ = 0.35 (*n*-pentane:ethyl acetate = 3:1).

^{1}**H NMR** (300 MHz, $CDCl_3$): δ = 7.37 – 7.17 (m, 2H), 6.90 (d, *J* = 8.0 Hz, 1H), 6.04 (s, 2H), 4.14 (q, *J* = 7.1 Hz, 2H), 2.30 (s, 3H), 1.17 (t, *J* = 7.1 Hz, 3H) ppm.

^{13}**C NMR** (75 MHz, $CDCl_3$): δ = 201.9, 168.2, 154.0, 153.2, 148.9, 128.0, 113.9, 109.7, 109.4, 102.5, 61.9, 45.3, 28.7, 14.2 ppm.

IR (ATR): $\tilde{\nu}$ = 592 (m), 727 (m), 817 (m), 863 (m), 1033 (s), 1219 (m), 1257 (s), 1446 (m), 1483 (s), 1700 (m), 1723 (s), 2909 (w), 2984 (w) cm^{-1}.

HRMS (EI): m/z calculated for $C_{14}H_{13}O_5N$ ($[M]^+$): 275.0788; found: 275.0796.

Ethyl 2-acetyl-3-(4-cyanophenyl)-2*H*-azirine-2-carboxylate (2i)

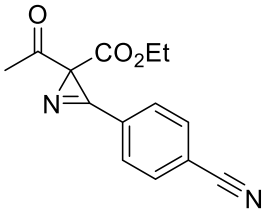

Ethyl 2-(amino(4-fluorophenyl)methylene)-3-oxobutanoate (129 mg, 0.50 mmol, 1.00 eq.) was reacted according to the general procedure until 2.4 F·mol^{-1} was applied. The pure product was obtained as colorless oil after flash chromatography over silica gel (*n*-pentane:ethyl acetate = 3:1). Remaining educt could be re-isolated (15.0 mg, 0.058 mmol).

Yield: 52% (67.0 mg, 0.262 mmol), 59% borsm.

$\mathbf{R_f}$ = 0.36 (*n*-pentane:ethyl acetate = 3:1).

^{1}**H NMR** (300 MHz, $CDCl_3$): δ = 7.97 (d, *J* = 8.4 Hz, 2H), 7.90 – 7.82 (m, 2H), 4.21 (q, *J* = 7.1 Hz, 2H), 2.48 (s, 3H), 1.23 (t, *J* = 7.1 Hz, 3H) ppm.

^{13}**C NMR** (75 MHz, $CDCl_3$): δ = 201.2, 167.7, 154.7, 133.2 (2C), 131.2 (2C), 124.6, 117.8, 117.4, 62.3, 45.3, 29.2, 14.1 ppm.

IR (ATR): $\tilde{\nu}$ = 570 (m), 847 (m), 1052 (m), 1300 (m), 1688 (m), 1717 (m), 2236 (w), 2360 (w), 2933 (w), 2979 (w), 3047 (w), 3091 (w) cm^{-1}.

HRMS (EI): m/z calculated for $C_{14}H_{12}O_3N_2$ ([M]+): 256.0842; found: 256.0849.

Ethyl 2-acetyl-3-(4-(trifluoromethyl)phenyl)-2*H*-azirine-2-carboxylate (2j)

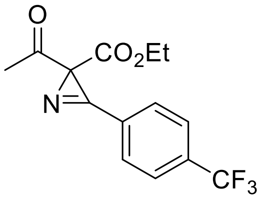

Ethyl 2-(amino(4-(trifluoromethyl)phenyl)methylene)-3-oxobutanoate (151 mg, 0.50 mmol, 1.00 eq.) was reacted according to the general procedure until 3.0 F·mol^{-1} was applied. The pure product was obtained as colorless oil after flash chromatography over silica gel (*n*-pentane:ethyl acetate = 5:1). Remaining educt could be re-isolated (6.00 mg, 0.02 mmol).

Yield: 51% (76.0 mg, 0.25 mmol), 53% borsm.

R$_f$ = 0.50 (*n*-pentane:ethyl acetate = 5:1).

^{1}H NMR (300 MHz, $CDCl_3$): δ = 7.99 (d, *J* = 8.1 Hz, 2H), 7.84 (d, *J* = 8.1 Hz, 2H), 4.22 (q, *J* = 7.1 Hz, 2H), 2.48 (s, 3H), 1.24 (t, *J* = 7.1 Hz, 3H) ppm.

^{13}C NMR (75 MHz, $CDCl_3$): δ = 201.3, 167.8, 154.6, 135.7 (q, *J* = 33.1 Hz, 1C), 131.2, 126.5 (q, *J* = 3.8 Hz, 1C), 123.9, 123.1 (q, *J* = 273.0 Hz, 1C), 62.1, 45.2, 29.0, 14.0 ppm.

^{19}F NMR (470 MHz, $CDCl_3$) δ = -171.7 ppm.

IR (ATR): $\tilde{\nu}$ = 601 (m), 847 (s), 1017 (s), 1063 (s), 1127 (s), 1170 (m), 1320 (s), 1361 (w), 1413 (m), 1702 (m), 1727 (m), 1784 (w), 2986 (w) cm^{-1}.

HRMS (EI): m/z calculated for $C_{14}H_{12}O_3NF_3$ ($[M]^+$): 299.0764; found: 299.0758.

Ethyl 2-acetyl-3-(*m*-tolyl)-2*H*-azirine-2-carboxylate (2k)

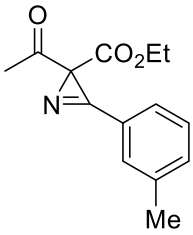

Ethyl 2-(amino(*m*-toyl)methylene)-3-oxobutanoate (117 mg, 0.47 mmol, 1.00 eq.) was reacted according to the general procedure until 2.4 F·mol^{-1} was applied. The pure product was obtained as colorless oil after flash chromatography over silica gel (*n*-pentane:ethyl acetate = 6:1).

Yield: 51% (59.0 mg, 0.241 mmol).

R$_f$ = 0.35 (*n*-pentane:ethyl acetate = 3:1).

^{1}H NMR (300 MHz, $CDCl_3$): δ = 7.65 – 7.54 (m, 2H), 7.44 – 7.34 (m, 2H), 4.15 (q, *J* = 7.2 Hz, 2H), 2.37 (s, 3H), 2.33 (s, 3H), 1.17 (t, *J* = 7.1 Hz, 3H) ppm.

^{13}C NMR (75 MHz, $CDCl_3$): δ = 201.8, 168.3, 154.8, 139.7, 135.5, 131.4, 129.5, 128.2, 120.2, 61.9, 45.1, 28.9, 21.3, 14.2 ppm.

IR (ATR): $\tilde{\nu}$ = 601 (m), 687 (m), 790 (m), 1047 (m), 1282 (m), 1359 (m), 1700 (s), 1724 (s), 2928 (w), 2983 (w) cm^{-1}.

HRMS (EI): m/z calculated for $C_{14}H_{15}O_3N$ ($[M]^+$): 245.1046; found: 245.1052.

Ethyl 2-acetyl-3-(*p*-tolyl)-2*H*-azirine-2-carboxylate (2l)

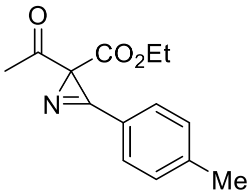

Ethyl 2-(amino(*p*-tolyl)methylene)-3-oxobutanoate (124 mg, 0.50 mmol, 1.00 eq.) was reacted according to the general procedure until 3.0 F·mol^{-1} was applied. The pure product was obtained as colorless oil after flash chromatography over silica gel (*n*-pentane:ethyl acetate = 5:1).

Yield: 61% (75 mg, 0.31 mmol).

$\mathbf{R_f}$ = 0.31 (*n*-pentane:ethyl acetate = 5:1).

^{1}**H NMR** (300 MHz, $CDCl_3$): δ = 7.78 – 7.66 (m, 2H), 7.42 – 7.30 (m, 2H), 4.19 (q, *J* = 7.1 Hz, 2H), 2.44 (s, 3H), 2.37 (s, 3H), 1.21 (t, *J* = 7.1 Hz, 3H) ppm.

^{13}**C NMR** (75MHz, $CDCl_3$): δ = 201.8, 168.3, 154.3, 146.0, 131.1 (2C), 130.4 (2C), 117.4, 61.9, 45.0, 28.8, 22.1, 14.1 ppm.

IR (ATR): $\tilde{\nu}$ = 517 (m), 566 (m), 597 (s), 826 (s), 1046 (s), 1284 (m), 1360 (m), 1606 (m), 1694 (s), 1720 (s), 1786 (m), 1993 (w).

HRMS (EI): m/z calculated for $C_{14}H_{15}O_3N$ ([M]$^+$): 245.1046; found: 245.1054.

Ethyl 2-acetyl-3-(furan-2-yl)-2*H*-azirine-2-carboxylate (2m)

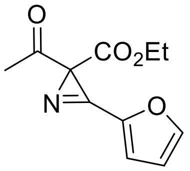

Ethyl 2-(amino(furan-2-yl)methylene)-3-oxobutanoate (112 mg, 0.50 mmol, 1.00 eq.) was reacted according to the general procedure until 3.6 F·mol^{-1} was applied. The pure product was obtained as brown oil after flash chromatography over silica gel (*n*-pentane:ethyl acetate = 3:1). Remaining educt could be re-isolated (10.0 mg, 0.045 mmol).

Yield: 15% (16 mg, 0.072 mmol), 16% borsm.

$\mathbf{R_f}$ = 0.30 (*n*-pentane:ethyl acetate = 3:1).

^{1}**H NMR** (300 MHz, $CDCl_3$): δ = 7.84 (dd, *J* = 1.8, 0.8 Hz, 1H), 7.32 (dd, *J* = 3.6, 0.7 Hz, 1H), 6.70 (dd, *J* = 3.7, 1.8 Hz, 1H), 4.22 (q, *J* = 7.1 Hz, 2H), 2.40 (s, 3H), 1.25 (t, *J* = 7.1 Hz, 3H) ppm.

^{13}**C NMR** (75 MHz, $CDCl_3$): δ = 201.3, 167.9, 150.0, 145.1, 137.4, 123.2, 113.4, 62.2, 44.4, 28.8, 14.2 ppm.

IR (ATR): $\tilde{\nu}$ = 591 (m), 764 (s), 1015 (m), 1047 (m), 1283 (m), 1462 (m), 1702 (s), 1726 (s), 1779 (w), 1984 (w), 3133 (w) cm^{-1}.

HRMS (EI): m/z calculated for $C_{11}H_{11}O_4N$ ([M]$^+$): 221.0683; found: 221.0678.

Ethyl 2-(cyclopropanecarbonyl)-3-phenyl-2*H*-azirine-2-carboxylate (2n)

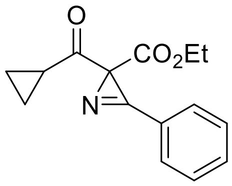

Ethyl 3-amino-2-(cyclopropanecarbonyl)-3-phenylacrylate (105 mg, 0.50 mmol, 1.00 eq.) was reacted according to the general procedure until 3.0 F·mol^{-1} was applied. The pure product was obtained as colorless oil after flash chromatography over silica gel (*n*-pentane:ethyl acetate = 5:1 → 3:1).

Yield: 30% (31.0 mg, 0.12 mmol).

R$_f$ = 0.30 (*n*-pentane:ethyl acetate = 5:1).

^{1}H NMR (300 MHz, $CDCl_3$): δ = 7.86 – 7.77 (m, 2H), 7.65 – 7.56 (m, 1H), 7.52 (dd, *J* = 8.1, 6.5 Hz, 2H), 4.17 (q, *J* = 7.1 Hz, 2H), 2.43 (tt, *J* = 8.1, 4.5 Hz, 1H), 1.18 (t, *J* = 7.1 Hz, 3H), 1.16 – 0.95 (m, 2H), 1.00 – 0.78 (m, 2H) ppm.

^{13}C NMR (75 MHz, $CDCl_3$): δ = 203.4, 168.4, 155.4, 134.4, 131.0 (2C), 129.5 (2C), 120.7, 61.9, 45.2, 18.7, 14.1, 12.7, 12.5 ppm.

IR (ATR): $\tilde{\nu}$ = 574 (m), 670 (m), 692 (s), 770 (s), 884 (m), 1037 (s), 1265 (s), 1383 (m), 1669 (m), 1724 (m), 1791 (m), 2360 (w), 2984 (w) cm^{-1}.

HRMS (EI): m/z calculated for $C_{15}H_{15}O_3N$ ([M]$^+$): 257.1046; found: 257.1053.

Ethyl 2-benzoyl-3-phenyl-2*H*-azirine-2-carboxylate (2o)

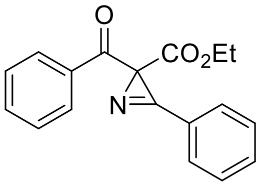

Ethyl 3-amino-2-benzoyl-3-phenylacrylate (148 mg, 0.50 mmol, 1.00 eq.) was reacted according to the general procedure until 3.0 F·mol^{-1} was applied. The pure product was obtained as colorless oil after flash chromatography over silica gel (*n*-pentane:ethyl acetate = 8:1 → 4:1 → 1:1).

Yield: 44% (64.0 mg, 0.22 mmol).

R$_f$ = 0.38 (*n*-pentane:ethyl acetate = 5:1).

^{1}H NMR (300 MHz, $CDCl_3$): δ = 8.10 – 8.03 (m, 2H), 7.99 – 7.85 (m, 2H), 7.63 – 7.35 (m, 6H), 4.10 (qd, *J* = 7.2, 2.8 Hz, 2H), 1.03 (t, *J* = 7.1 Hz, 3H) ppm.

^{13}C NMR (75 MHz, $CDCl_3$): δ = 194.3, 169.5, 157.9, 135.6, 134.5, 133.7, 131.1 (2C), 129.5 (2C), 129.3 (2C), 128.7 (2C), 121.2, 62.2, 43.4, 14.0 ppm.

IR (ATR): $\tilde{\nu}$ = 683 (m), 946 (m), 1094 (m), 1247 (s), 1449 (m), 1666 (m), 1736 (m), 1766 (w), 2360 (w), 2937 (w), 3060 (w) cm^{-1}.

HRMS (EI): m/z calculated for $C_{18}H_{15}O_3N$ ([M]+): 293.1046; found: 293.1037.

1-(3-Phenyl-2*H*-azirin-2-yl)ethan-1-one (2p)

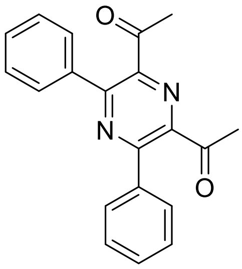

Ethyl 2-(amino(phenyl)methylene)-3-oxobutanoic acid (103 mg, 0.50 mmol, 1.00 eq.) was reacted according to the general procedure until 3.3 F·mol^{-1} was applied. The pure product was obtained as yellowish solid after flash chromatography over silica gel (*n*-pentane:ethyl acetate = 5:1).

Melting point: Decomposition above 198 °C.

Yield: 28% (28.0 mg, 0.14 mmol).

R$_f$ = 0.50 (*n*-pentane:ethyl acetate = 4:1).

^{1}H NMR (300 MHz, CDCl$_3$): δ = 7.82 – 7.60 (m, 4H), 7.60 – 7.38 (m, 6H), 2.78 (s, 6H) ppm.

^{13}C NMR (75 MHz, CDCl$_3$): δ = 200.1 (2C), 153.1 (2C), 144.1 (2C), 136.6 (2C), 130.4 (2C), 129.6 (4C), 128.7 (4C), 28.5 (2C) ppm.

IR (ATR): $\tilde{\nu}$ = 611 (m), 690 (s), 766 (m), 950 (m), 1351 (m), 1522 (m), 1699 (s), 2360 (w), 2923 (w), 3001 (w), 3030 (w), 3387 (w) cm^{-1}.

HRMS (ESI): m/z calculated for $C_{20}H_{17}O_2N_2$ ([M]+H$^+$): 317.1290; found: 317.1283.

Diethyl 3-phenyl-2*H*-azirine-2,2-dicarboxylate (2q)

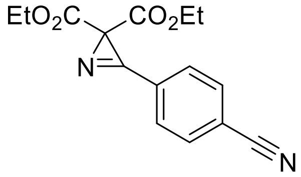

Diethyl 2-(amino(phenyl)methylene)malonate (144 mg, 0.50 mmol, 1.00 eq.) was reacted according to the general procedure until 3.0 F·mol^{-1} was applied. The pure product was obtained as yellowish oil after flash chromatography over silica gel (*n*-pentane:ethyl acetate = 5:1).

Yield: 35% (50.0 mg, 0.17 mmol).

R$_f$ = 0.38 (*n*-pentane:ethyl acetate = 4:1).

^{1}H NMR (300 MHz, CDCl$_3$): δ = 8.04 (d, *J* = 8.2 Hz, 2H), 7.89 (d, *J* = 8.2 Hz, 2H), 4.24 (q, *J* = 7.1 Hz, 4H), 1.26 (t, *J* = 7.1 Hz, 6H) ppm.

^{13}C NMR (75 MHz, CDCl$_3$): δ = 166.7 (2C), 155.8, 133.2 (2C), 131.3 (2C), 124.6, 117.9, 117.5, 62.5 (2C), 39.7, 14.1 (2C) ppm.

IR (ATR): $\tilde{\nu}$ = 573 (m), 731 (m), 846 (m), 1016 (m), 1066 (s), 1256 (m), 1289 (m), 1727 (s), 2233 (w), 2984 (w) cm^{-1}.

HRMS (EI): m/z calculated for $C_{15}H_{14}O_4N_2$ ([M]$^+$): 286.0948; found: 286.0949.

Diethyl 3-(4-(methoxycarbonyl)phenyl)-2*H*-azirine-2,2-dicarboxylate (2r)

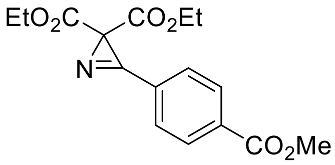

Diethyl 2-(amino(4-(methoxycarbonyl)phenyl)methylene)malonate (161 mg, 0.50 mmol, 1.00 eq.) was reacted according to the general procedure until 3.3 F·mol^{-1} was applied. The pure product was obtained as colorless oil after flash chromatography over silica gel (*n*-pentane:ethyl acetate = 4:1 → 3:1).

Yield: 30% (47.0 mg, 0.15 mmol).

$\mathbf{R_f}$ = 0.27 (*n*-pentane:ethyl acetate = 4:1).

^{1}H NMR (300 MHz, $CDCl_3$): δ = 8.18 (d, *J* = 8.3 Hz, 2H), 7.93 (d, *J* = 8.2 Hz, 2H), 4.18 (q, *J* = 7.1 Hz, 4H), 3.90 (s, 3H), 1.20 (t, *J* = 7.1 Hz, 6H) ppm.

^{13}C NMR (75 MHz, $CDCl_3$): δ = 167.0 (2C), 165.7, 155.7, 135.3, 130.9 (2C), 130.6 (2C), 124.3, 62.3 (2C), 52.8, 39.4, 14.1 (2C) ppm.

IR (ATR): $\tilde{\nu}$ = 691 (m), 770 (m), 862 (m), 1016 (m), 1066 (s), 1106 (m), 1274 (s), 1722 (s), 2956 (w), 2984 (w) cm^{-1}.

HRMS (EI): m/z calculated for $C_{16}H_{17}O_6N$ ($[M]^+$): 319.1050; found: 319.1054.

Diethyl 3-(4-bromophenyl)-2*H*-azirine-2,2-dicarboxylate (2s)

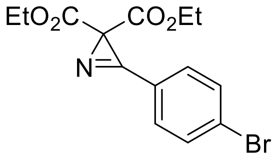

Diethyl 2-(amino(4-bromophenyl)methylene)malonate (150 mg, 0.44 mmol, 1.00 eq.) was reacted according to the general procedure until 2.4 F·mol^{-1} was applied. The pure product was obtained as red oil after flash chromatography over silica gel (*n*-pentane:ethyl acetate = 5:1).

Yield: 40% (60.0 mg, 0.18 mmol).

$\mathbf{R_f}$ = 0.35 (*n*-pentane:ethyl acetate = 5:1).

^{1}H NMR (300 MHz, $CDCl_3$): δ = 7.83 – 7.57 (m, 4H), 4.17 (q, *J* = 7.1 Hz, 4H), 1.19 (t, *J* = 7.1 Hz, 6H) ppm.

^{13}C NMR (75 MHz, $CDCl_3$): δ = 167.0 (2C), 155.2, 133.0 (2C), 132.1 (2C), 129.8, 119.4, 62.1 (2C), 39.1, 14.1 (2C) ppm.

IR (ATR): $\tilde{\nu}$ = 563 (m), 829 (m), 1010 (s), 1064 (s), 1252 (m), 1293 (m), 1369 (m), 1482 (m), 1587 (m), 1724 (s), 2939 (w), 2983 (w) cm^{-1}.

HRMS (EI): m/z calculated for $C_{14}H_{14}O_4NBr$ ($[M]^+$): 339.0101; found: 339.0096.

General procedure for the synthesis of 4-carboxy-oxazoles

Azirine compound was dissolved in a vessel tube and heated to 150 °C until complete conversion could be determined by GC-MS analysis. The pure product was obtained after evaporation of the solvent in quantitative yield.

Ethyl 5-methyl-2-phenyloxazole-4-carboxylate (3a)

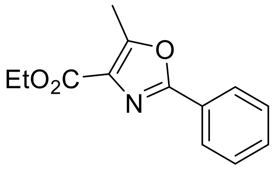

Azirine **2a** (40.0 mg, 0.17 mmol) was dissolved in MeCN and heated for 24 h. The product was obtained as colorless oil.

Yield: 100% (40.0 mg, 0.17 mmol).

^{1}H NMR (300 MHz, $CDCl_3$): δ = 8.06 (dd, J = 6.8, 3.0 Hz, 2H), 7.51 – 7.24 (m, 3H), 4.41 (q, J = 7.1 Hz, 2H), 2.69 (s, 3H), 1.41 (t, J = 7.1 Hz, 3H) ppm.

^{13}C NMR (75 MHz, $CDCl_3$): δ = 162.6, 159.7, 156.3, 130.8, 128.9, 128.8 (2C), 126.7, 126.7 (2C), 61.1, 14.5, 12.3 ppm.

The analytical data is in accordance with the literature.[1]

Ethyl 2-(4-fluorophenyl)-5-methyloxazole-4-carboxylate (3b)

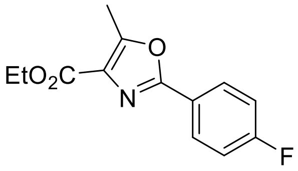

Azirine **2b** (73.0 mg, 0.29 mmol) was dissolved in MeCN and heated for 24 h. The product was obtained as yellowish oil.

Yield: 100% (73.0 mg, 0.29 mmol).

^{1}H NMR (300 MHz, $CDCl_3$): δ = 8.00 – 7.93 (m, 2H), 7.07 – 7.02 (m, 2H), 4.33 (q, J = 7.1 Hz, 2H), 2.60 (s, 3H), 1.33 (t, J = 7.1 Hz, 3H) ppm.

^{13}C NMR (75 MHz, $CDCl_3$): δ = 164.3 (d, J = 251.7 Hz), 163.3, 162.4, 158.9, 156.2, 128.8 (d, J = 8.7 Hz, 2C), 123.1 (d, J = 3.2 Hz), 116.0 (d, J = 22.2 Hz, 2C), 61.1, 14.5, 12.2 ppm.

The analytical data is in accordance with the literature.[2]

Ethyl 2-(4-(methoxycarbonyl)phenyl)-5-methyloxazole-4-carboxylate (3c)

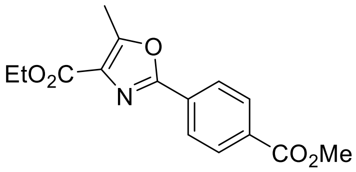

Azirine **2e** (65.0 mg, 0.23 mmol) was dissolved in toluene and heated for 24 h. The product was obtained as brown oil.

Yield: 100% (65.0 mg, 0.23 mmol).

^{1}H NMR (300 MHz, $CDCl_3$): *δ* = 8.11 (d, *J* = 1.8 Hz, 4H), 4.41 (q, *J* = 7.1 Hz, 2H), 3.92 (s, 3H), 2.70 (s, 3H), 1.41 (t, *J* = 7.1 Hz, 3H) ppm.

^{13}C NMR (75 MHz, $CDCl_3$): *δ* = 166.4, 162.3, 158.8, 157.0, 131.9, 130.4, 130.1 (2C), 129.3, 126.5 (2C), 61.3, 52.5, 14.5, 12.4 ppm.

IR (ATR): $\tilde{\nu}$ = 714 (s), 774 (s), 1015 (s), 1102 (s), 1187 (s), 1284 (s), 1591 (m), 1718 (s), 2363 (w), 2850 (w), 2970 (w) cm^{-1}.

HRMS (EI): m/z calculated for $C_{15}H_{15}O_5N$ ($[M]^+$): 289.0945; found: 289.0935.

Ethyl 2-(4-cyanophenyl)-5-methyloxazole-4-carboxylate (3d)

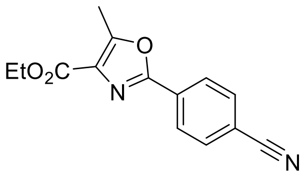

Azirine **2i** (59.0 mg, 0.23 mmol) was dissolved in toluene and heated for 120 h. The product was obtained as brown solid.

Yield: 100% (59.0 mg, 0.23 mmol).

Melting point: 165-168 °C.

^{1}H NMR (300 MHz, $CDCl_3$): *δ* = 8.10 (d, *J* = 8.3 Hz, 2H), 7.68 (d, *J* = 8.4 Hz, 2H), 4.36 (q, *J* = 7.2 Hz, 2H), 2.66 (s, 3H), 1.35 (t, *J* = 7.1 Hz, 3H) ppm.

^{13}C NMR (75 MHz, $CDCl_3$): *δ* = 162.1, 157.8, 157.3, 132.6 (2C), 130.4, 129.6, 127.0 (2C), 118.2, 114.1, 61.4, 14.4, 12.4 ppm.

IR (ATR): $\tilde{\nu}$ = 551 (m), 700 (m), 338 (m), 834 (m), 1011 (m), 1104 (m),1223 (w), 1376 (w), 1597 (w), 1724 (m), 2224 (w), 2853 (w), 2923 (w) cm^{-1}.

HRMS (EI): m/z calculated for $C_{14}H_{13}O_5N$ ($[M]^+$): 275.0788; found: 275.0787.

Ethyl 5-methyl-2-(*p*-tolyl)oxazole-4-carboxylate (3e)

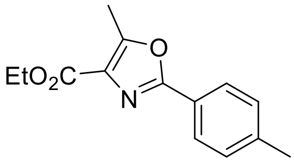

Azirine **2l** (53.0 mg, 0.22 mmol) was dissolved in toluene and heated for 24 h. The product was obtained as colorless oil.

Yield: 100% (53.0 mg, 0.22 mmol).

^{1}H NMR (300 MHz, $CDCl_3$): δ = 7.87 (d, J = 7.9 Hz, 2H), 7.17 (d, J = 7.9 Hz, 2H), 4.34 (q, J = 7.3 Hz, 2H), 2.61 (s, 3H), 2.31 (s, 3H), 1.33 (t, J = 6.9 Hz, 3H) ppm.

^{13}C NMR (75 MHz, $CDCl_3$): δ = 162.6, 159.9, 155.9, 141.1, 129.4 (2C), 128.6, 126.5 (2C), 123.9, 61.0, 21.5, 14.4, 12.2 ppm.

The analytical data is in accordance with the literature.[3]

Ethyl 5-cyclopropyl-2-phenyloxazole-4-carboxylate (3f)

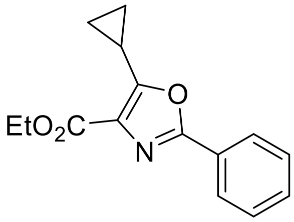

Azirine **2n** (29.1 mg, 0.11 mmol) was dissolved in toluene and heated for 24 h. The product was obtained as brown solid.

Yield: 100% (29.1 mg, 0.11 mmol).

Melting point: 66-69 °C.

^{1}H NMR (300 MHz, $CDCl_3$): δ = 8.25 – 7.86 (m, 2H), 7.44 – 7.39 (m, 3H), 4.44 (q, J = 7.2 Hz, 2H), 2.89 – 2.75 (m, 1H), 1.42 (t, J = 7.1 Hz, 3H), 1.19 (d, J = 4.4 Hz, 2H), 1.17 (s, 2H) ppm.

^{13}C NMR (75 MHz, $CDCl_3$): δ = 162.9, 161.0, 158.2, 130.7, 128.8, 128.3 (2C), 126.7, 126.6 (2C), 61.1, 14.6, 9.5 (2C), 8.2 ppm.

IR (ATR): $\tilde{\nu}$ = 690 (s), 710 (s), 772 (m), 785 (s), 1055 (s), 1170 (s), 1383 (s), 1586 (w), 1720 (s), 2983 (w) cm^{-1}.

HRMS (EI): m/z calculated for $C_{15}H_{15}O_3N$ ($[M]^+$): 257.1046; found: 257.1038.

Ethyl 2-(4-methoxyphenyl)-5-methyloxazole-4-carboxylate (3g)

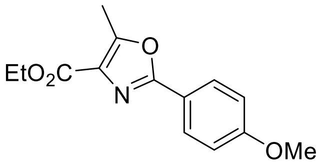

Azirine **2d** (56.0 mg, 0.21 mmol) was dissolved in toluene and heated for 24 h. The product was obtained as yellow solid.

Yield: 100% (56.0 mg, 0.21 mmol).

^{1}H NMR (300 MHz, $CDCl_3$): *δ* = 8.00 (d, *J* = 8.1 Hz, 2H), 6.95 (d, *J* = 8.1 Hz, 2H), 4.41 (q, *J* = 7.0 Hz, 2H), 3.85 (s, 3H), 2.68 (s, 3H), 1.41 (t, *J* = 7.0 Hz, 3H) ppm.

^{13}C NMR (75 MHz, $CDCl_3$): *δ* = 162.7, 161.7, 159.9, 155.8, 128.7, 128.4 (2C), 119.5, 114.2 (2C), 61.1, 55.5, 14.5, 12.3 ppm.

The analytical data is in accordance with the literature.[2]

Ethyl 2,5-diphenyloxazole-4-carboxylate (3h)

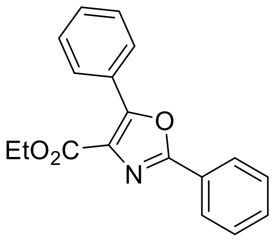

Azirine **2o** (57.0 mg, 0.19 mmol) was dissolved in toluene and heated for 24 h. The product was obtained as colorless solid.

Yield: 100% (57.0 mg, 0.19 mmol).

^{1}H NMR (300 MHz, $CDCl_3$): *δ* = 8.09 – 8.05 (m, 2H), 8.05 – 8.00 (m, 2H), 7.45 – 7.35 (m, 6H), 4.37 (q, *J* = 7.1 Hz, 2H), 1.34 (t, *J* = 7.1 Hz, 3H) ppm.

^{13}C NMR (75 MHz, $CDCl_3$): *δ* = 160.4, 158.0, 153.2, 129.2, 128.4, 127.0 (2C), 126.7 (2C), 126.5 (2C), 126.5, 125.3, 125.0 (2C), 124.6, 59.6, 12.5 ppm.

The analytical data is in accordance with the literature.[4]

Ethyl 2-(benzo[d][1,3]dioxol-5-yl)-5-methyloxazole-4-carboxylate (3i)

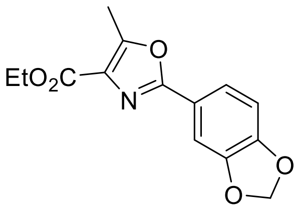

Azirine **2h** (77.0 mg, 0.28 mmol) was dissolved in toluene and heated for 24 h. The product was obtained as brown solid.

Yield: 100% (77.0 mg, 0.28 mmol).

Melting point: 147-150 °C.

^{1}H NMR (300 MHz, $CDCl_3$): *δ* = 7.57 (d, *J* = 8.2 Hz, 1H), 7.49 (s, 1H), 6.83 (d, *J* = 8.2 Hz, 1H), 5.99 (s, 2H), 4.38 (q, *J* = 7.1, 6.6 Hz, 2H), 2.64 (s, 3H), 1.38 (t, *J* = 7.1 Hz, 3H) ppm.

^{13}C NMR (75 MHz, $CDCl_3$): *δ* = 162.6, 159.5, 155.8, 149.9, 148.1, 128.7, 121.5, 120.8, 108.6, 106.9, 101.7, 61.1, 14.5, 12.3 ppm.

IR (ATR): $\tilde{\nu}$ = 728 (s), 783 (s), 795 (s), 924 (s), 1019 (s), 1092 (s), 1257 (s), 1484 (s), 1723 (s), 2907 (w), 2963 (w) cm^{-1}.

HRMS (EI): m/z calculated for $C_{14}H_{13}O_5N$ ([M]$^+$): 275.0788; found: 275.0787.

General procedure for the synthesis of enamines

Enamines were synthesized according to a modified procedure from Thomas *et al.* (**GP1** and **GP2**)[5] or Armstrong *et al.* (**GP3**)[6] All other enamines were used from the group's inventory, which were prepared according to previously published procedures.[7-8]

GP1: To a stirred solution of the benzonitrile in dry toluene (75 mL) ethyl acetoacetate was added followed by stannic chloride at room temperature under argon atmosphere and refluxed in an oven dried flask for 3 h. The mixture was cooled to room temperature and quenched carefully with saturated aqueous Na_2CO_3 solution (200 mL). The mixture was extracted with ethyl acetate (3 × 250 mL). The combined extracts were washed with water (2 × 100 mL) and dried over anhydrous Na_2SO_4. The residue obtained after evaporation of the solvent was purified by flash chromatography on silica gel.

GP2: To a stirred solution of benzonitrile in dry toluene (15 mL) ethyl acetoacetate was added followed by stannic chloride at room temperature under argon atmosphere in an oven dried vessel tube and heated to 110 °C for 3 h. The mixture was cooled to room temperature and quenched carefully with saturated aqueous Na_2CO_3 solution (50 mL). The mixture was extracted with ethyl acetate (3 × 50 mL). The combined extracts were washed with water (2 × 50 mL) and dried over anhydrous $MgSO_4$. The residue obtained after evaporation of the solvent was purified by flash chromatography on silica gel.

GP3: To a stirred solution of the β-keto ester (1.00 eq.) in methanol (50 mL) NH_4OAc (4.80 eq.) was added and heated to 50 °C for 24 h. The residue obtained after evaporation of the solvent was diluted with ethyl acetate (30 mL), washed with water (2 × 30 mL), brine (2 × 30 mL), and dried over anhydrous $MgSO_4$. The residue obtained after evaporation of the solvent was purified by flash chromatography on silica gel.

Ethyl 2-(amino(phenyl)methylene)-3-oxobutanoate (1a)

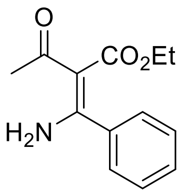

Benzonitrile (3.40 g, 33.0 mmol, 1.00 eq.), ethyl acetoacetate (4.17 mL, 33.0 mmol, 1.00 eq.) and stannic chloride (3.89 mL, 33.3 mmol, 1.01 eq.) were reacted according to the **GP1**. The pure product was obtained as white solid after flash chromatography over silica gel (*n*-pentane:ethyl acetate = 3:1 → 2:1 → 1:1).

Yield: 48% (3.65 g, 15.7 mmol).

^{1}H NMR (300 MHz, $CDCl_3$): δ = 10.92 (s, 1H), 7.80 – 6.94 (m, 5H), 5.54 (s, 1H), 3.67 (q, J = 7.2 Hz, 2H), 2.29 (s, 3H), 0.64 (t, J = 7.1 Hz, 3H) ppm.

^{13}C NMR (75 MHz, $CDCl_3$): δ = 197.5, 170.2, 167.2, 138.9, 130.4, 129.0 (2C), 127.0 (2C), 104.6, 60.4, 29.9, 13.8 ppm.

The analytical data is in accordance with the literature.[8]

Ethyl 2-(amino(4-fluorophenyl)methylene)-3-oxobutanoate (1b)

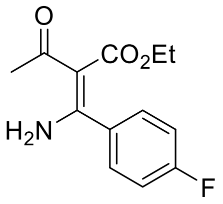

4-Fluorobenzonitrile (4.00 g, 33.0 mmol, 1.00 eq.), ethyl acetoacetate (4.17 mL, 33.0 mmol, 1.00 eq.) and stannic chloride (3.89 mL, 33.3 mmol, 1.01 eq.) were reacted according to the **GP1**. The pure product was obtained as white solid after flash chromatography over silica gel (*n*-pentane:ethyl acetate = 3:1 → 2:1 → 1:1).

Yield: 54% (4.47 g, 17.8 mmol).

^{1}H NMR (300 MHz, $CDCl_3$): δ = 10.96 (s, 1H), 7.43 – 7.31 (m, 2H), 7.10 (t, J = 8.6 Hz, 2H), 5.49 (s, 1H), 3.79 (q, J = 7.1 Hz, 2H), 2.35 (s, 3H), 0.80 (t, J = 7.2 Hz, 3H) ppm.

^{13}C NMR (75 MHz, $CDCl_3$): δ = 196.8, 169.6, 165.8, 163.6 (d, J = 250.3 Hz), 134.3 (d, J = 3.4 Hz, 2C), 128.8 (d, J = 8.5 Hz, 2C), 115.6 (d, J = 21.9 Hz), 104.1, 60.0, 29.3, 13.4 ppm.

^{19}F NMR (470 MHz, $CDCl_3$) δ = -110.2 ppm.

The analytical data is in accordance with the literature.[8]

Ethyl 2-(amino(4-bromophenyl)methylene)-3-oxobutanoate (1c)

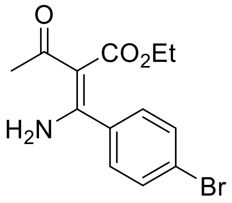

4-Bromobenzonitrile (1.09 g, 6.00 mmol, 1.00 eq.), ethyl acetoacetate (0.76 mL, 6.00 mmol, 1.00 eq.) and stannic chloride (0.71 mL, 33.3 mmol, 1.01 eq.) were reacted according to the **GP2**. The pure product was obtained as white solid after flash chromatography over silica gel (*n*-pentane:ethyl acetate = 3:1 → 2:1 → 1:1).

Yield: 59% (1.10 g, 3.52 mmol).

^{1}H NMR (300 MHz, $CDCl_3$): δ = 10.85 (s, 1H), 7.65 – 7.34 (m, 2H), 7.18 (d, *J* = 8.5 Hz, 2H), 5.36 (s, 1H), 3.76 (q, *J* = 7.1 Hz, 2H), 2.30 (s, 3H), 0.76 (t, *J* = 7.1 Hz, 3H) ppm.

^{13}C NMR (75 MHz, $CDCl_3$): δ = 198.0, 169.5, 165.4, 137.4, 132.0 (2C), 128.4 (2C), 124.4, 104.5, 60.3, 29.8, 13.6 ppm.

The analytical data is in accordance with the literature.[8]

Ethyl 2-(amino(4-methoxyphenyl)methylene)-3-oxobutanoate (1d)

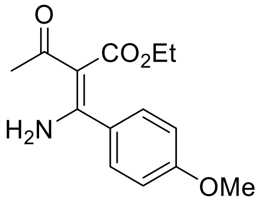

4-Methoxybenzonitrile (0.80 g, 6.00 mmol, 1.00 eq.), ethyl acetoacetate (0.76 mL, 6.00 mmol, 1.00 eq.) and stannic chloride (0.71 mL, 33.3 mmol, 1.01 eq.) were reacted according to the **GP2**. The pure product was obtained as white solid after flash chromatography over silica gel (*n*-pentane:ethyl acetate = 2:1 → 1:1).

Yield: 55% (865 mg, 3.29 mmol).

^{1}H NMR (300 MHz, $CDCl_3$): δ = 10.93 (s, 1H), 7.30 (d, *J* = 8.8 Hz, 2H), 6.89 (d, *J* = 8.7 Hz, 2H), 5.71 (s, 1H), 3.81 (d, *J* = 5.3 Hz, 3H), 3.85 – 3.74 (m, 2H), 2.32 (s, 3H), 0.80 (t, *J* = 7.2 Hz, 3H) ppm.

^{13}C NMR (75 MHz, $CDCl_3$): δ = 196.6, 170.3, 166.6, 161.2, 130.6, 128.3 (2C), 114.1 (2C), 104.1, 60.2, 55.5, 29.4, 13.7 ppm.

The analytical data is in accordance with the literature.[8]

Ethyl 2-(amino(4-nitrophenyl)methylene)-3-oxobutanoate (1f)

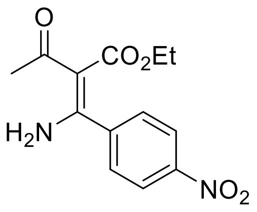

4-Nitrobenzonitrile (0.89 g, 6.00 mmol, 1.00 eq.), ethyl acetoacetate (0.76 mL, 6.00 mmol, 1.00 eq.) and stannic chloride (0.71 mL, 33.3 mmol, 1.01 eq.) were reacted according to the **GP2**. The pure product was obtained as white solid after flash chromatography over silica gel (*n*-pentane:ethyl acetate = 5:1 → 2:1 → 1:1).

Yield: 48% (803 mg, 2.89 mmol).

^{1}H NMR (300 MHz, $CDCl_3$): *δ* = 11.04 (s, 1H), 8.52 – 8.17 (m, 2H), 7.66 – 7.44 (m, 2H), 5.37 (s, 1H), 3.82 (q, *J* = 7.1 Hz, 2H), 2.41 (s, 3H), 0.82 (t, *J* = 7.2 Hz, 3H) ppm.

^{13}C NMR (75 MHz, $CDCl_3$): *δ* = 198.0, 168.7, 164.4, 148.5, 144.5, 128.0 (2C), 123.9 (2C), 104.3, 60.4, 30.1, 13.6 ppm.

The analytical data is in accordance with the literature.[9]

Ethyl 2-(amino(3-nitrophenyl)methylene)-3-oxobutanoate (1g)

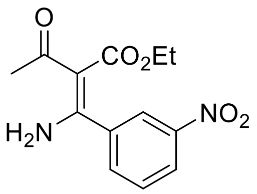

3-Nitrobenzonitrile (0.89 g, 6.00 mmol, 1.00 eq.), ethyl acetoacetate (0.76 mL, 6.00 mmol, 1.00 eq.) and stannic chloride (0.71 mL, 33.3 mmol, 1.01 eq.) were reacted according to the **GP2**. The pure product was obtained as white solid after flash chromatography over silica gel (*n*-pentane:ethyl acetate = 2:1 → 1:1).

Yield: 62% (1.03 g, 3.69 mmol).

^{1}H NMR (300 MHz, $CDCl_3$): *δ* = 11.02 (s, 1H), 8.45 – 8.15 (m, 2H), 7.77 – 7.51 (m, 2H), 5.55 (s, 1H), 3.82 (q, *J* = 7.1 Hz, 2H), 2.39 (s, 3H), 0.82 (t, *J* = 7.1 Hz, 3H) ppm.

^{13}C NMR (75 MHz, $CDCl_3$): *δ* = 197.9, 168.8, 164.0, 148.2, 139.8, 132.9, 129.9, 124.6, 122.1, 104.4, 60.3, 30.1, 13.7 ppm.

The analytical data is in accordance with the literature.[8]

Ethyl 2-(amino(4-fluorophenyl)methylene)-3-oxobutanoate (1h)

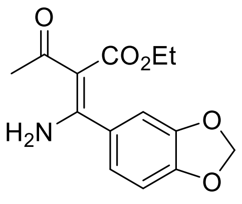

Benzo[*d*][1,3]dioxole-5-carbonitrile (0.88 g, 6.00 mmol, 1.00 eq.), ethyl acetoacetate (0.76 mL, 6.00 mmol, 1.00 eq.) and stannic chloride (0.71 mL, 33.3 mmol, 1.01 eq.) were reacted according to the **GP2**. The pure product was obtained as white solid after flash chromatography over silica gel (*n*-pentane:ethyl acetate = 5:1 → 2:1 → 1:1).

Yield: 63% (1.05 g, 3.80 mmol).

Melting point: 144-147 °C

$\mathbf{R_f}$ = 0.10 (*n*-pentane:ethyl acetate = 5:1).

^{1}**H NMR** (300 MHz, $CDCl_3$): δ = 10.90 (s, 1H), 7.05 – 6.55 (m, 3H), 5.99 (s, 2H), 5.58 (s, 1H), 3.85 (q, *J* = 7.0 Hz, 2H), 2.31 (s, 3H), 0.88 (t, *J* = 7.2 Hz, 3H) ppm.

^{13}**C NMR** (75 MHz, $CDCl_3$): δ = 196.8, 170.0, 166.1, 149.3, 148.0, 132.1, 121.0, 108.6, 107.4, 104.3, 101.7, 60.3, 29.5, 13.8 ppm.

IR (ATR): $\tilde{\nu}$ = 546 (w), 668 (w), 809 (w), 914 (m), 1030 (m), 1080 (m), 1114 (m), 1232 (m), 1276 (m), 1444 (m), 1592 (m), 1679 (m), 1740 (w), 2363 (w), 2982 (w), 3313 (w) cm^{-1}.

HRMS (EI): m/z calculated for $C_{14}H_{15}O_5N$ ($[M]^+$): 277.0945; found: 277.0947.

Ethyl 2-(amino(4-cyanophenyl)methylene)-3-oxobutanoate (1i)

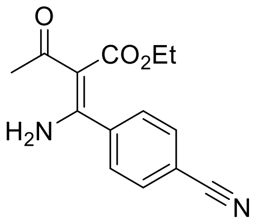

Terephthalonitrile (0.59 mL, 6.00 mmol, 1.00 eq.), ethyl acetoacetate (0.76 mL, 6.00 mmol, 1.00 eq.) and stannic chloride (0.71 mL, 33.3 mmol, 1.01 eq.) were reacted according to the **GP2**. The pure product was obtained as white solid after flash chromatography over silica gel (*n*-pentane:ethyl acetate = 2:1 → 1:1).

Yield: 19% (288 mg, 1.12 mmol).

Melting point: 162-165 °C.

$\mathbf{R_f}$ = 0.10 (*n*-pentane:ethyl acetate = 5:1).

^{1}**H NMR** (300 MHz, $CDCl_3$): δ = 11.01 (s, 1H), 7.72 (d, *J* = 7.6 Hz, 2H), 7.48 (d, *J* = 7.8 Hz, 2H), 5.43 (s, 1H), 3.79 (q, *J* = 7.0 Hz, 2H), 2.39 (s, 3H), 0.79 (t, *J* = 6.8 Hz, 3H) ppm.

^{13}**C NMR** (75 MHz, $CDCl_3$): δ = 198.0, 168.8, 164.5, 142.9, 132.6 (2C), 127.7 (2C), 118.1, 113.8, 104.5, 60.4, 30.1, 13.6 ppm.

IR (ATR): $\tilde{\nu}$ = 584 (m), 599 (w), 852 (w), 1044 (w), 1116 (m), 1267 (m), 1287 (m), 1363 (w), 1464 (m), 1600 (w), 1697 (w), 2229 (w), 2363 (w), 2970 (w), 3386 (w) cm^{-1}.

HRMS (EI): m/z calculated for $C_{14}H_{14}O_3N_2$ ($[M]^+$): 258.0999; found: 258.1004.

Ethyl 2-(amino(4-(trifluoromethyl)phenyl)methylene)-3-oxobutanoate (1j)

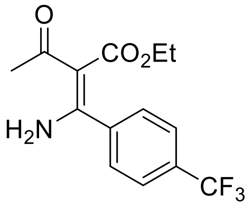

4-Trifluoromethylbenzonitrile (3.00 g, 17.4 mmol, 1.00 eq.), ethyl acetoacetate (2.28 g, 17.4 mmol, 1.00 eq.) and stannic chloride (1.14 mL, 17.5 mmol, 1.01 eq.) were reacted according to the **GP1**. The pure product was obtained as white solid after flash chromatography over silica gel (*n*-pentane:ethyl acetate = 3:1).

Yield: 24% (1.28 g, 4.28 mmol).

Melting point: 132-135 °C.

$\mathbf{R_f}$ = 0.35 (*n*-pentane:ethyl acetate = 3:1).

^{1}**H NMR** (300 MHz, $CDCl_3$): δ = 10.99 (s, 1H), 7.68 (d, J = 8.1 Hz, 2H), 7.49 (d, J = 8.0 Hz, 2H), 5.48 (s, 1H), 3.77 (q, J = 7.1 Hz, 2H), 2.38 (s, 3H), 0.73 (t, J = 7.1 Hz, 3H) ppm.

^{13}**C NMR** (75 MHz, $CDCl_3$): δ = 197.8, 169.1, 165.1, 142.1, 127.3, 132.2 (q, J = 32.9 Hz), 125.76 (q, J = 3.7 Hz), 123.8 (q, J = 272.4 Hz), 104.6, 60.3, 29.9, 13.4 ppm.

^{19}**F NMR** (470 MHz, $CDCl_3$) δ = -62.9 ppm.

IR (ATR): $\tilde{\nu}$ = 604 (s), 676 (s), 862 (s), 1052 (s), 1108 (s), 1266 (s), 1464 (s), 1704 (s), 2344 (w), 2970 (w), 3282 (s) cm^{-1}.

HRMS (EI): m/z calculated for $C_{14}H_{14}O_3NF_3$ ($[M]^+$): 301.0920; found: 301.0915.

Ethyl 2-(amino(*p*-tolyl)methylene)-3-oxobutanoate (1l)

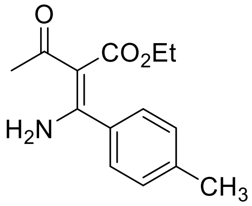

4-Methylbenzonitrile (0.72 g, 6.00 mmol, 1.00 eq.), ethyl acetoacetate (0.76 mL, 6.00 mmol, 1.00 eq.) and stannic chloride (0.71 mL, 33.3 mmol, 1.01 eq.) were reacted according to the **GP2**. The pure product was obtained as white solid after flash chromatography over silica gel (*n*-pentane:ethyl acetate = 3:1).

Yield: 42% (615 mg, 2.48 mmol).

Melting point: 97-100 °C.

$\mathbf{R_f}$ = 0.17 (*n*-pentane:ethyl acetate = 5:1).

^{1}**H NMR** (300 MHz, $CDCl_3$): δ = 10.99 (s, 1H), 7.70 – 6.61 (m, 4H), 5.55 (s, 1H), 3.80 (q, J = 7.3 Hz, 2H), 2.40 (s, 3H), 2.37 (s, 3H), 0.77 (t, J = 6.9 Hz, 3H) ppm.

^{13}C NMR (75 MHz, $CDCl_3$): δ = 197.0, 170.1, 167.0, 140.5, 135.7, 129.4 (2C), 126.7 (2C), 104.3, 60.2, 29.6, 21.5, 13.5 ppm.

IR (ATR): $\tilde{\nu}$ = 668 (w), 754 (w), 730 (w), 1057 (w), 1140 (w), 1264 (w), 1290 (w), 1357 (w), 1457 (w), 1602 (w), 1668 (w), 1739 (w), 2360 (w), 2983 (w), 3330 (w) cm^{-1}.

HRMS (EI): m/z calculated for $C_{14}H_{17}O_3N$ ($[M]^+$): 247.1203; found: 247.1207.

Ethyl 3-amino-3-phenylacrylate (1p)

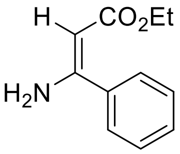

Ethyl 3-oxo-3-phenylpropanoate (4.81 g, 25.0 mmol, 1.00 eq.) and NH_4OAc (9.25 g, 120 mmol, 4.80 eq.) were reacted according to the **GP3**. The pure product was obtained as colorless oil after flash chromatography over silica gel (*n*-pentane:diethyl ether = 20:1).

Yield: 62% (2.94 g, 15.3 mmol).

^{1}H NMR (300 MHz, $CDCl_3$): δ = 9.87 (s, 1H), 7.58 – 7.48 (m, 2H), 7.47 – 7.37 (m, 3H), 5.18 (s, 1H), 4.96 (s, 1H), 4.17 (q, *J* = 7.1 Hz, 2H), 1.30 (t, *J* = 7.1 Hz, 3H) ppm.

^{13}C NMR (75 MHz, $CDCl_3$): δ = 170.5, 160.6, 137.8, 130.3, 128.9 (2C), 126.3 (2C), 84.7, 59.0, 14.7 ppm.

The analytical data is in accordance with the literature.[10]

Ethyl 2-acetyl-3-amino-5-phenylpenta-2,4-dienoate (1t)

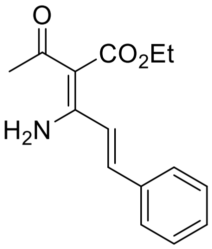

Cinnamonitrile (0.75 mL, 6.00 mmol, 1.00 eq.), ethyl acetoacetate (0.76 mL, 6.00 mmol, 1.00 eq.) and stannic chloride (0.71 mL, 33.3 mmol, 1.01 eq.) were reacted according to the **GP2**. The pure product was obtained as yellowish solid after flash chromatography over silica gel (*n*-pentane:ethyl acetate = 4:1).

Yield: 60% (0.92 mg, 3.56 mmol).

^{1}H NMR (300 MHz, $CDCl_3$): δ = 10.93 (s, 1H), 7.52 – 7.43 (m, 2H), 7.42 – 7.31 (m, 3H), 7.19 (d, *J* = 16.2 Hz, 1H), 7.05 (d, *J* = 16.2 Hz, 1H), 5.67 (s, 1H), 4.27 (q, *J* = 7.1 Hz, 2H), 2.36 (s, 3H), 1.31 (t, *J* = 7.1 Hz, 3H) ppm.

^{13}C NMR (75 MHz, $CDCl_3$): δ = 197.7, 169.4, 162.0, 135.4, 135.2, 129.6, 129.0 (2C), 127.5 (2C), 124.8, 104.0, 60.6, 30.5, 14.4 ppm.

The analytical data is in accordance with the literature.[8]

3-Amino-2,3-diphenylacrylonitrile (1v)

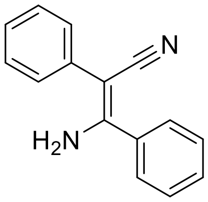

According to a procedure reported by Chang *et al.* benzyl cyanide (0.57 mL, 5.00 mmol, 1.00 eq.) and benzonitrile (0.51 mL, 5.00 mmol, 1.00 eq.) were treated with *t*-BuOK (1.40 g, 12.5 mmol, 2.50 eq.) in *t*-BuOH (10 mL) and stirred at room temperature for 3 h. The reaction was quenched with water (20 mL), extracted with ethyl acetate (3 × 20 mL), and dried over anhydrous $MgSO_4$. The pure product was obtained as yellow solid after flash chromatography over silica gel (*n*-pentane:ethyl acetate = 4:1 → 3:1).

Yield: 42% (466 mg, 2.12 mmol).

^{1}H NMR (300 MHz, $CDCl_3$): δ = 7.73 – 7.67 (m, 2H), 7.58 – 7.40 (m, 7H), 7.38 – 7.29 (m, 1H), 4.78 (s, 2H) ppm.

^{13}C NMR (75 MHz, $CDCl_3$): δ = 157.3, 136.0, 134.0, 130.6, 129.4 (2C), 128.8 (2C), 128.6 (2C), 128.1 (2C), 127.4, 122.5, 81.2 ppm.

The analytical data is in accordance with the literature.[11]

Reaction optimization by *Design of Experiments* for numerical parameters

D-optimal screening design was generated to cover all numerical parameters using the JMP 13 software package by SAS (version *13.2.1*. SAS Institute Inc., Cary, NC, 2016). All linear and quadratic terms were considered. An extension of the design was performed to cover the cross interactions between temperature × electric current, electrolyte concentration × temperature and iodine loading × temperature as well. The design contains of 21 reactions to cover all terms and 4 replications for the determination of the *lack of fit* value. The model was verified by k-fold cross-validation (k = 5). All reactions were carried out on a 0.50 mmol scale. Liquid starting materials were measured using *Eppendorf*^{TM} pipettes or by weighing in exactly.

In a H-type cell, both compartments were charged with NEt_4OTs in acetonitrile (each compartment 10 mL). Sodium iodide, 2,6-lutidine and enamine **1b** (126 mg, 1.00 eq., 0.50 mmol) were added to the anode compartment. The cathode compartment was equipped with platinum electrode, the anode compartment was equipped with graphite electrode. The reaction was electrolyzed under constant current (10 mA·cm^{-2}) until the desired current was passed through the solution. The yields were determined by ^{19}F NMR using *2*-nitro-fluorobenzene (52.6 μL, 1.00 eq., 0.50 mmol) as internal standard.

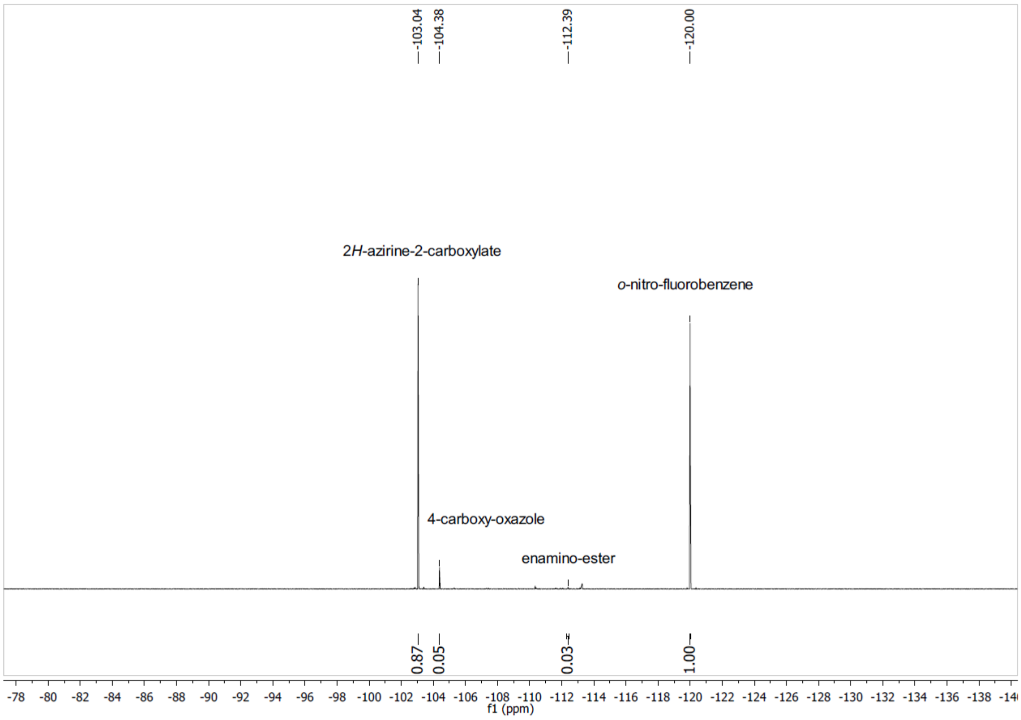

Figure 4: Typical ^{19}F NMR spectra of the reaction with 2-nitro-fluorobenzene as internal standard.

Table 3: Reaction optimization using *Design of Experiments*: optimization table for numerical parameters. All reactions were carried out on a 0.5 mmol scale using substrate **2b**. Yields were determined by ^{19}F NMR spectroscopy using 2-nitro-fluorobenzene as the internal standard. The predicted optimal reaction conditions were tested twice.

Nr.	*Iodine loading*	*Base eq.*	*c [mol·L^{-1}]*	*T [°C]*	*Charge [F·mol^{-1}]*	*I [mA]*	*Yield [%]*	*Educt [%]*
**1**	0.20	1.0	0.16	40	2.1	10	37	0
**2**	0.25	2.0	0.08	55	2.1	10	52	11
**3**	0.30	3.0	0.24	25	2.1	10	82	10
**4**	0.20	2.0	0.08	40	1.8	6	56	12
**5**	0.30	3.0	0.16	25	1.8	6	58	17
**6**	0.25	1.0	0.24	55	1.8	6	44	14
**7**	0.25	1.0	0.08	25	2.4	10	37	1
**8**	0.30	2.0	0.24	40	2.4	10	72	2
**9**	0.20	3.0	0.16	55	2.4	10	60	2
**10**	0.20	3.0	0.24	55	2.4	8	64	5
**11**	0.30	1.0	0.08	40	2.4	8	33	0
**12**	0.25	2.0	0.16	25	2.4	8	70	0
**13**	0.30	1.0	0.16	55	2.2	6	43	0
**14**	0.25	3.0	0.24	40	2.2	6	70	4
**15**	0.20	2.0	0.08	25	2.2	6	42	0
**16**	0.20	1.0	0.16	25	1.8	10	38	1
**17**	0.25	3.0	0.08	40	1.8	10	51	22
**18**	0.30	2.0	0.24	55	1.8	10	52	30
**19**	0.20	1.0	0.24	25	2.0	8	42	9
**20**	0.25	2.0	0.16	40	2.0	8	62	14
**21**	0.30	3.0	0.08	55	2.0	8	51	18
**Replications**	0.20	2.0	0.08	40	1.8	6	58	23
	0.25	1.0	0.08	25	2.4	10	31	3
	0.20	1.0	0.24	25	2.0	8	45	1
	0.20	1.0	0.24	25	2.0	8	45	14
**Optimal**	0.30	2.4	0.24	25	2.4	10	87	4
**Replication**	0.30	2.4	0.24	25	2.4	10	87	3

Actual by Predicted Plot

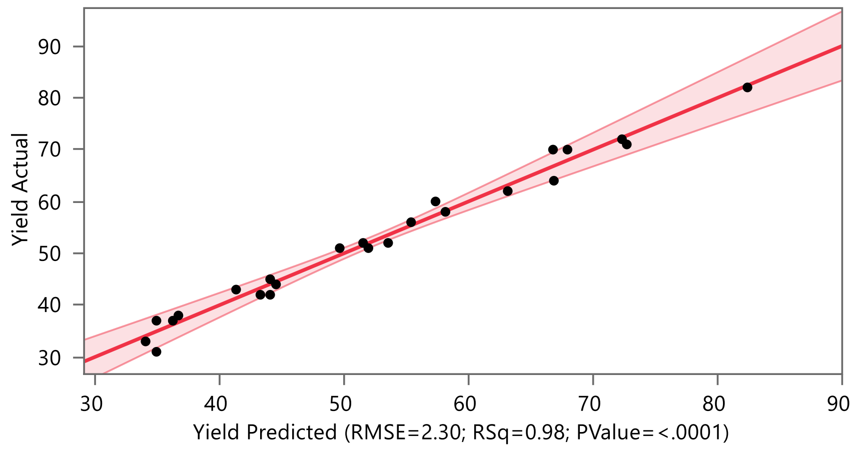

Figure 5: Reaction optimization using *Design of Experiments*: Predicted yields are plotted versus measured yields (in total 24 experiments: 21 for the optimization, 4 for replication of different experiments).

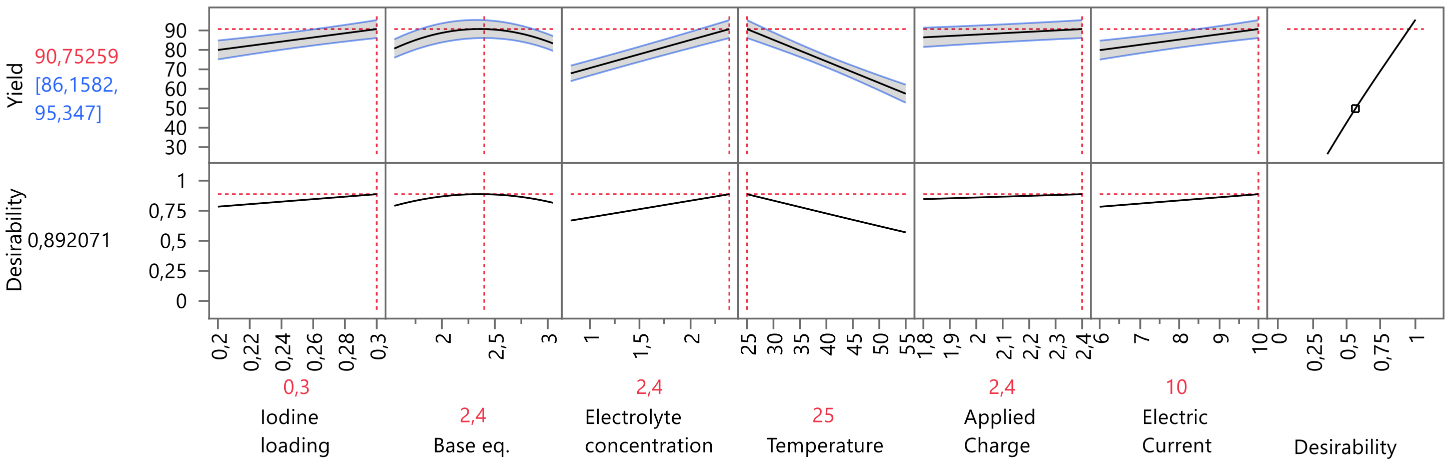

Figure 6: Reaction optimization using *Design of Experiments*: predicted optimal reaction conditions.

Effect Summary

Source	LogWorth	PValue
Base eq. × Base eq.	8,207	0,00000
Electrolyte concentration (0,8,2,4)	8,039	0,00000
Temperature × Electric Current	5,234	0,00001
Electrolyte concentration× Temperature	4,788	0,00002
Iodine loading × Temperature	3,899	0,00013
Applied Charge (1,8,2,4)	2,116	0,00766
Base eq. (2,3)	2,074	0,00843 ^
Electric Current (6,10)	0,943	0,11394 ^
Temperature (25,55)	0,685	0,20652 ^
Iodine loading (0,2,0,3)	0,537	0,29070 ^

Lack Of Fit

Source	DF	Sum of Squares	Mean Square	F Ratio
Lack Of Fit	11	49,837287	4,53066	0,5663
Pure Error	3	24,000000	8,00000	**Prob > F**
Total Error	14	73,837287		0,7883
				**Max RSq**
				0,9947

Residual by Predicted Plot

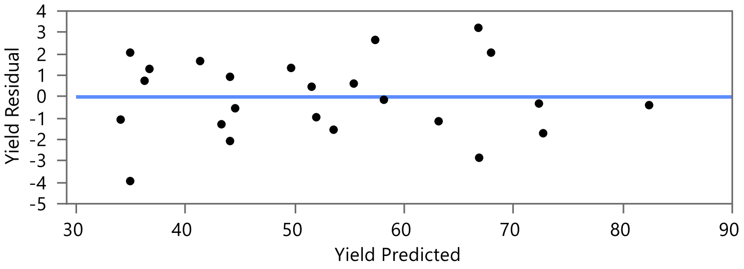

Studentized Residuals

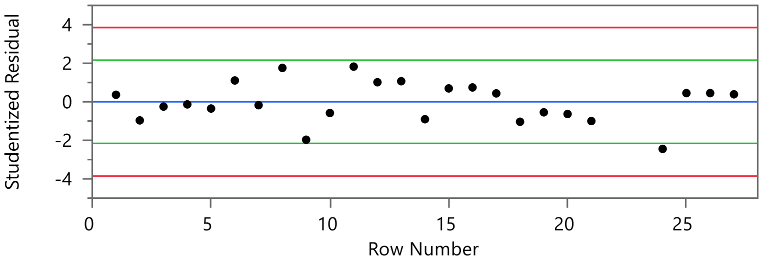

Externally studentized residuals with 95% simultaneous limits (Bonferroni) in red, individual limits in green.

Summary of Fit

RSquare	0,983806
RSquare Adj	0,972238
Root Mean Square Error	2,296539
Mean of Response	52,32
Observations (or Sum Wgts)	25

Analysis of Variance

Source	DF	Sum of Squares	Mean Square	F Ratio
Model	10	4485,6027	448,560	85,0498
Error	14	73,8373	5,274	**Prob > F**
C. Total	24	4559,4400		<,0001*

Parameter Estimates

Term	Estimate	Std Error	t Ratio	Prob>|t|
Intercept	65,459023	0,831499	78,72	<,0001*
Iodine loading (0,2,0,3)	0,679014	0,618369	1,10	0,2907
Base eq. (2,3)	-2,17391	0,709675	-3,06	0,0084*
Electrolyte concentration (0,8,2,4)	6,6974417	0,557162	12,02	<,0001*
Temperature (25,55)	-0,807795	0,609844	-1,32	0,2065
Applied Charge (1,8,2,4)	2,1211451	0,681761	3,11	0,0077*
Electric Current (6,10)	-1,031372	0,611721	-1,69	0,1139
Iodine loading × Temperature	-4,66346	0,890838	-5,23	0,0001*
Electrolyte concentration× Temperature	-4,649103	0,725437	-6,41	<,0001*
Temperature × Electric Current	-6,423375	0,912014	-7,04	<,0001*
Base eq. × Base eq.	-3,779366	0,305088	-12,39	<,0001*

Residual by Row Plot

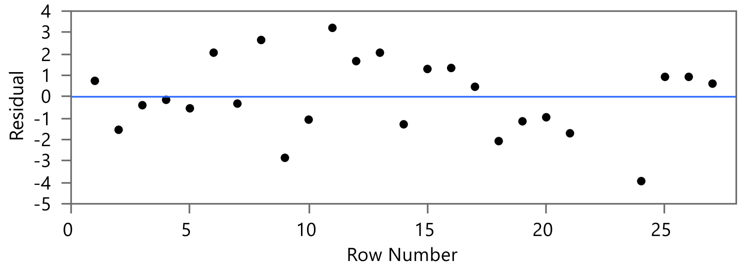

Press

Press	Press RMSE	Press RSquare
222,23426694	2,98150477	0,9513

Box-Cox Transformations

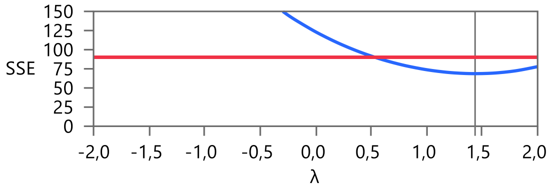

Best λ=1,44

Reaction optimization by *Design of Experiments* for categorical parameters

D-optimal screening design was generated to cover all categorical parameters using the JMP 13 software package by SAS. All linear and quadratic terms were considered. An extension of the design was performed to cover the cross interactions as well. The design contains of 65 reactions to cover all terms. Since *DoE*s with categorical parameters are very difficult to solve, this model should be regarded as a rough extensive screening to find the best combination of categorical parameters. For a more precise analysis, all possible combinations of all categorical parameters must be examined (in total 1620 experiments). All reactions were carried out on a 0.50 mmol scale. Liquid starting materials were measured using *Eppendorf*™ pipettes or by weighing in exactly.

In a H-type cell, both compartments were charged with a supporting electrolyte in respective solvent (each compartment 10 mL). An iodine source, base and enamine **1b** (126 mg, 1.00 eq., 0.50 mmol) were added to the anode compartment. The reaction was electrolyzed under constant current until the desired current was passed through the solution. The yields were determined by ^{19}F NMR using *2*-nitro-fluorobenzene (52.6 μL, 1.00 eq., 0.50 mmol) as internal standard.

Table 4: Reaction optimization using *Design of Experiments*: optimization table for categorical parameters. All reactions were carried out on a 0.5 mmol scale using substrate **2b**. Yields were determined by ^{19}F NMR spectroscopy using 2-nitro-fluorobenzene as the internal standard.

Nr.	*Solvent*	*Iodine source*	*Iodine eq.*	*Base*	*Base eq.*	*Electrolyte*	*c [mol·L^{-1}]*	*T [°C]*	*Charge [F·mol^{-1}]*	*I [mA]*	*Anode*	*Kathode*	*Yield [%]*
1	MeOH	KI	0.25	Pyridine	3.0	NBu_4BF_4	0.20	25	1.8	5.0	Graphite	Gl. carbon	0
2	DMF	NBu_4I	0.20	DBU	2.0	NEt_4OTs	0.30	40	1.8	5.0	Gl. carbon	Gl. carbon	47
3	DMF	NaI	0.20	DBU	3.0	NBu_4BF_4	0.10	55	1.8	5.0	Platinum	Platinum	45
4	MeOH	PhI	0.30	KOPiv	3.0	NEt_4OTs	0.30	25	1.8	5.0	Gl. carbon	Graphite	4
5	MeCN	PhI	0.30	Pyridine	2.0	$LiClO_4$	0.20	40	1.8	5.0	Platinum	Platinum	0
6	MeCN	KI	0.25	Lutidine	1.0	$LiClO_4$	0.10	55	1.8	5.0	Graphite	Graphite	17
7	MeCN	NaI	0.20	Lutidine	3.0	NEt_4OTs	0.30	25	1.8	7.5	Graphite	Platinum	71
8	MeOH	NaI	0.30	Lutidine	1.0	NBu_4BF_4	0.10	40	1.8	7.5	Gl. carbon	Gl. carbon	9
9	MeCN	KI	0.30	DBU	3.0	NBu_4BF_4	0.20	40	1.8	7.5	Platinum	Graphite	33
10[a]	DMF	PhI	0.25	KOPiv	1.0	NEt_4OTs	0.20	55	1.8	7.5	Graphite	Gl. carbon	38
11	MeOH	NBu_4I	0.25	Pyridine	2.0	$LiClO_4$	0.10	55	1.8	7.5	Gl. carbon	Graphite	27
12	DMF	KI	0.20	KOPiv	1.0	$LiClO_4$	0.30	25	1.8	7.5	Platinum	Platinum	42
13	DMF	NaI	0.30	Pyridine	1.0	NBu_4BF_4	0.30	55	2.4	10	Graphite	Graphite	1
14	DMF	PhI	0.25	Lutidine	1.0	$LiClO_4$	0.20	40	2.4	10	Gl. carbon	Platinum	1
15	MeCN	NaI	0.25	KOPiv	2.0	NEt_4OTs	0.10	40	2.4	10	Platinum	Gl. carbon	13
16	MeCN	NBu_4I	0.20	KOPiv	3.0	NBu_4BF_4	0.20	55	2.4	10	Gl. carbon	Platinum	5
17	MeOH	KI	0.20	Lutidine	2.0	NEt_4OTs	0.20	55	2.4	10	Platinum	Graphite	1
18	MeOH	PhI	0.20	DBU	3.0	$LiClO_4$	0.10	25	2.4	10	Graphite	Gl. carbon	1
19	MeCN	KI	0.30	KOPiv	3.0	$LiClO_4$	0.30	55	2.1	5.0	Gl. carbon	Gl. carbon	17
20	MeOH	NBu_4I	0.30	DBU	1.0	NEt_4OTs	0.20	55	2.1	5.0	Graphite	Platinum	0
21	DMF	NBu_4I	0.25	Lutidine	2.0	NBu_4BF_4	0.30	25	2.1	5.0	Graphite	Graphite	1
22	MeCN	PhI	0.20	Pyridine	2.0	NEt_4OTs	0.10	25	2.1	5.0	Platinum	Graphite	0
23	MeOH	PhI	0.25	KOPiv	2.0	NBu_4BF_4	0.10	40	2.1	5.0	Graphite	Platinum	8
24	DMF	NaI	0.20	Pyridine	2.0	$LiClO_4$	0.20	25	2.1	5.0	Gl. carbon	Gl. carbon	32

25	DMF	KI	0.20	Pyridine	2.0	NEt_4OTs	0.10	40	2.1	10	Gl. carbon	Graphite	16
26	MeCN	KI	0.30	DBU	2.0	NEt_4OTs	0.10	25	2.1	10	Graphite	Platinum	36
27	MeCN	NaI	0.25	DBU	2.0	NBu_4BF_4	0.20	25	2.1	10	Gl. carbon	Graphite	34
28	MeOH	NaI	0.25	Pyridine	2.0	$LiClO_4$	0.30	40	2.1	10	Platinum	Platinum	19
29	MeOH	PhI	0.20	Lutidine	2.0	NBu_4BF_4	0.30	55	2.1	10	Platinum	Gl. carbon	0
30	DMF	NBu_4I	0.30	KOPiv	2.0	$LiClO_4$	0.10	25	2.1	10	Platinum	Graphite	71
31	MeCN	NBu_4I	0.20	Pyridine	2.0	NBu_4BF_4	0.30	40	2.4	7.5	Graphite	Gl. carbon	1
32	MeCN	PhI	0.25	DBU	2.0	$LiClO_4$	0.30	55	2.4	7.5	Platinum	Graphite	0
33	DMF	PhI	0.30	Pyridine	2.0	NBu_4BF_4	0.10	55	2.4	7.5	Gl. carbon	Platinum	1
34	DMF	NBu_4I	0.30	Lutidine	2.0	NEt_4OTs	0.20	25	2.4	7.5	Platinum	Gl. carbon	56
35	MeOH	KI	0.25	DBU	2.0	NEt_4OTs	0.30	25	2.4	7.5	Gl. carbon	Platinum	0
36	MeOH	NaI	0.20	KOPiv	2.0	$LiClO_4$	0.20	40	2.4	7.5	Graphite	Graphite	32
37[a]	DMF	NBu_4I	0.23	Lutidine	2.2	$LiClO_4$	0.1	25	2.0	8.0	Graphite	Graphite	0
38	MeCN	NaI	0.20	Lutidine	3.0	NBu_4BF_4	0.3	25	2.4	7.5	Graphite	Platinum	3
39	DMF	NBu_4I	0.23	Lutidine	2.2	NEt_4OTs	0.3	25	2.0	8.0	Graphite	Graphite	47
40	MeCN	NaI	0.20	Lutidine	3.0	NEt_4OTs	0.3	25	2.4	7.5	Graphite	Platinum	88
41	MeCN	NaI	0.20	Lutidine	3.0	NEt_4OTs	0.3	25	1.8	7.5	Graphite	Platinum	72
42	DMF	Bu4NI	0.30	Lutidine	2.0	NEt_4OTs	0.2	25	2.4	7.5	Platinum	Gl. carbon	59
43	MeCN	KI	0.30	DBU	3.0	NBu_4BF_4	0.2	40	1.8	7.5	Platinum	Graphite	32
44	DMF	KI	0.20	KOPiv	1.0	$LiClO_4$	0.3	25	1.8	7.5	Platinum	Platinum	41
45	MeCN	KI	0.30	KOPiv	3.0	$LiClO_4$	0.3	55	2.1	5.0	Gl. carbon	Gl. carbon	14
46	DMF	NBu_4I	0.25	Lutidine	2.0	NBu_4BF_4	0.3	25	2.1	5.0	Graphite	Graphite	2
47	MeCN	NaI	0.20	Lutidine	3.0	NEt_4OTs	0.3	25	2.5	7.5	Graphite	Platinum	80
48	DMF	NBu_4I	0.23	Lutidine	2.2	$LiClO_4$	0.1	25	2.0	8.0	Graphite	Graphite	64
49	MeCN	NaI	0.20	Lutidine	3.0	NBu_4BF_4	0.3	25	2.4	7.5	Graphite	Platinum	3
50	MeCN	NaI	0.20	Lutidine	3.0	NEt_4OTs	0.3	25	2.4	8.0	Graphite	Platinum	80
51	MeCN	NaI	0.20	Lutidine	3.0	NEt_4OTs	0.3	25	2.4	7.5	Graphite	Platinum	82

52	MeCN	NaI	0.20	Lutidine	3.0	NEt_4OTs	0.3	25	2.4	8.0	Platinum	Graphite	84
53	MeCN	NBu_4I	0.20	KOPiv	1.0	NEt_4OTs	0.3	25	2.2	10	Platinum	Platinum	16
54	MeOH	NaI	0.20	DBU	2.0	$LiClO_4$	0.3	40	1.8	10	Graphite	Graphite	6
55	MeCN	NBu_4I	0.30	DBU	3.0	$LiClO_4$	0.1	25	1.8	10	Platinum	Gl. carbon	7
56	MeCN	PhI	0.20	KOPiv	2.0	$LiClO_4$	0.1	40	1.8	10	Graphite	Gl. carbon	0
57	MeOH	NBu_4I	0.30	Lutidine	3.0	$LiClO_4$	0.3	55	1.8	10	Gl. carbon	Gl. carbon	60
58	MeCN	KI	0.30	Pyridine	2.0	NEt_4OTs	0.3	25	1.8	10	Gl. carbon	Platinum	23
59	DMF	NaI	0.25	Lutidine	2.0	$LiClO_4$	0.2	40	1.8	10	Graphite	Gl. carbon	57
60	DMF	KI	0.25	Lutidine	3.0	NBu_4BF_4	0.3	40	2.4	5.0	Platinum	Gl. carbon	5
61	MeCN	NaI	0.30	Pyridine	2.0	NEt_4OTs	0.2	25	2.4	5.0	Platinum	Platinum	40
62	DMF	PhI	0.30	DBU	2.0	NBu_4BF_4	0.1	25	2.4	5.0	Graphite	Graphite	50
63	DMF	NaI	0.30	KOPiv	1.0	NBu_4BF_4	0.3	25	2.4	5.0	Graphite	Gl. carbon	27
64	DMF	NaI	0.20	Lutidine	3.0	NEt_4OTs	0.3	25	2.4	8.0	Graphite	Platinum	52
65	DMF	NaI	0.20	Lutidine	2.5	NEt_4OTs	0.3	25	2.0	8.0	Graphite	Gl. carbon	50

[a] Reactions not included in the model, because they were identified as outliers.

Actual by Predicted Plot

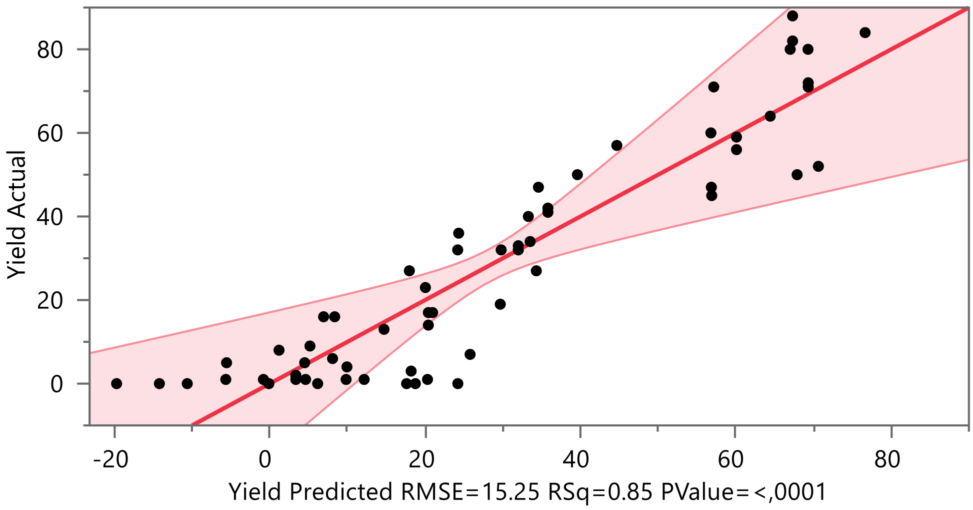

Figure 7: Reaction optimization using *Design of Experiments*: Predicted yields are plotted versus measured yields (65 experiments).

Effect Summary

Source	LogWorth	PValue
Supporting electrolyte × Base	3,561	0,00027
Iodine source	2,318	0,00481
Electrolyte concentration× Electrolyte concentration	1,818	0,01522
Temperature (25,55)	1,483	0,03291
Base eq. (1,3)	1,045	0,09022
Supporting electrolyte	0,727	0,18766 ^
Base	0,683	0,20769 ^
Anode material	0,536	0,29135
Iodine loading × Iodine loading	0,487	0,32589
Iodine loading (0,2,0,3)	0,420	0,38061 ^
Base eq. × Base eq.	0,258	0,55186
Charge density × Charge density	0,257	0,55287
Applied charge × Applied charge	0,226	0,59415
Applied charge (1,8,2,4)	0,139	0,72625 ^
Charge density (5,10)	0,093	0,80646 ^
Solvent	0,090	0,81364
Temperature × Temperature	0,070	0,85151
Electrolyte concentration (0,1,0,3)	0,031	0,93203 ^
Cathode material	0,007	0,98397

Residual by Predicted Plot

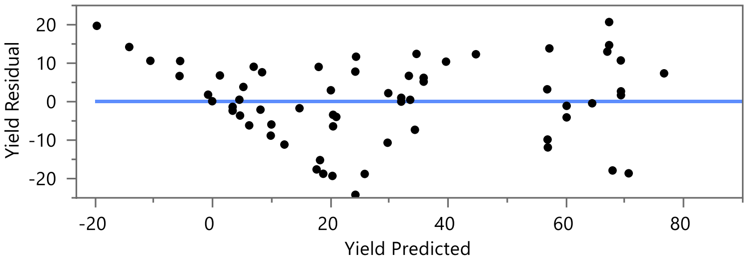

Studentized Residuals

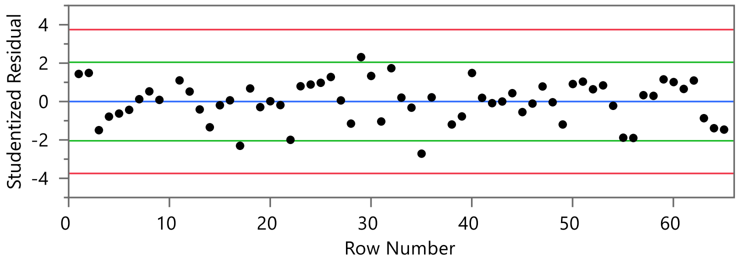

Externally studentized residuals with 95% simultaneous limits (Bonferroni) in red, individual limits in green.

Parameter Estimates

Term	Estimate	Std Error	t Ratio	Prob>|t|
Intercept	4,028866	9,446255	0,43	0,6728
Solvent [MeCN]	-1,473411	3,137532	-0,47	0,6420
Solvent [DMF]	2,1560857	3,683458	0,59	0,5627
Iodine source [NaI]	12,554088	3,962881	3,17	0,0035*
Iodine source [KI]	-7,995931	4,778994	-1,67	0,1047
Iodine source [PhI]	-10,27039	4,93695	-2,08	0,0461*
Iodine loading (0,2,0,3)	2,3823447	2,677135	0,89	0,3806
Base [Lutidine]	9,7365018	4,556549	2,14	0,0409*
Base [DBU]	-0,46893	3,930177	-0,12	0,9058
Base [Pyridine]	-6,950844	4,669516	-1,49	0,1470
Base eq. (1,3)	6,8120073	3,891001	1,75	0,0902
Supporting electrolyte [$LiClO_4$]	4,4092628	3,465246	1,27	0,2130
Supporting electrolyte [TBATFB]	-6,142688	3,579876	-1,72	0,0965
Electrolyte concentration (0,1,0,3)	-0,245166	2,850444	-0,09	0,9320
Temperature (25,55)	-7,553247	3,377447	-2,24	0,0329*
Applied charge (1,8,2,4)	-1,006237	2,847243	-0,35	0,7263
Charge density (5,10)	-0,828025	3,350017	-0,25	0,8065
Anode material [Platinum]	5,6378359	3,543543	1,59	0,1221
Anode material [Graphite]	-3,844954	4,083495	-0,94	0,3539
Cathode material [Platinum]	0,2383677	3,741085	0,06	0,9496
Cathode material [Graphite]	0,3994459	3,611149	0,11	0,9127
Iodine loading × Iodine loading	6,4520772	6,459905	1,00	0,3259
Base eq. × Base eq.	-3,806005	6,324798	-0,60	0,5519
Electrolyte concentration× Electrolyte concentration	14,6176	5,677999	2,57	0,0152*
Temperature × Temperature	1,0801233	5,720539	0,19	0,8515
Applied charge × Applied charge	2,9829535	5,538589	0,54	0,5942
Charge density × Charge density	-3,570511	5,948768	-0,60	0,5529
Supporting electrolyte [$LiClO_4$] × Base [Lutidine]	16,683554	6,300052	2,65	0,0128*
Supporting electrolyte [$LiClO_4$] × Base [DBU]	-26,34633	7,2219	-3,65	0,0010*
Supporting electrolyte [$LiClO_4$] × Base [Pyridine]	0,4651916	6,606032	0,07	0,9443
Supporting electrolyte [NBu_4BF_4] × Base [Lutidine]	-28,95644	6,015339	-4,81	<,0001*
Supporting electrolyte [NBu_4BF_4] × Base [DBU]	22,178629	7,470549	2,97	0,0058*
Supporting electrolyte [NBu_4BF_4] × Base [Pyridine]	-1,282876	7,703434	-0,17	0,8689

Effect Tests

Source	Nparm	DF	Sum of Squares	F Ratio	Prob > F
Solvent	2	2	96,5669	0,2077	0,8136
Iodine source	3	3	3683,3795	5,2805	0,0048*
Iodine loading (0,2,0,3)	1	1	184,1276	0,7919	0,3806
Base	3	3	1123,3135	1,6104	0,2077

Source	Nparm	DF	Sum of Squares	F Ratio	Prob > F
Base eq. (1,3)	1	1	712,6526	3,0650	0,0902
Supporting electrolyte	2	2	823,1166	1,7700	0,1877
Electrolyte concentration (0,1,0,3)	1	1	1,7201	0,0074	0,9320
Temperature (25,55)	1	1	1162,8954	5,0014	0,0329*
Applied charge (1,8,2,4)	1	1	29,0403	0,1249	0,7263
Charge density (5,10)	1	1	14,2051	0,0611	0,8065
Anode material	2	2	597,7275	1,2854	0,2913
Cathode material	2	2	7,5174	0,0162	0,9840
Iodine loadingIodine loading	1	1	231,9515	0,9976	0,3259
Base eq. × Base eq.	1	1	84,1968	0,3621	0,5519
Electrolyte concentration× Electrolyte concentration	1	1	1541,0323	6,6277	0,0152*
Temperature × Temperature	1	1	8,2894	0,0357	0,8515
Applied charge × Applied charge	1	1	67,4443	0,2901	0,5942
Charge density × Charge density	1	1	83,7639	0,3603	0,5529
Supporting electrolyte × Base	6	6	8572,4221	6,1447	0,0003*

Press

Press	Press RMSE	Press RSquare
39219,929659	24,9507228	0,1567

Scaled Estimates

Nominal factors expanded to all levels

Term	Scaled Estimate	Std Error	t Ratio	Prob>|t|
Intercept	4,028866	9,446255	0,43	0,6728
Solvent [MeCN]	-1,473411	3,137532	-0,47	0,6420
Solvent [DMF]	2,1560857	3,683458	0,59	0,5627
Solvent [MeOH]	-0,682674	3,838749	-0,18	0,8600
Iodine source [NaI]	12,554088	3,962881	3,17	0,0035*
Iodine source [KI]	-7,995931	4,778994	-1,67	0,1047
Iodine source [PhI]	-10,27039	4,93695	-2,08	0,0461*
Iodine source [Bu_4NI]	5,7122367	4,227157	1,35	0,1867
Iodine loading (0,2,0,3)	2,3823447	2,677135	0,89	0,3806
Base [Lutidine]	9,7365018	4,556549	2,14	0,0409*
Base [DBU]	-0,46893	3,930177	-0,12	0,9058
Base [Pyridine]	-6,950844	4,669516	-1,49	0,1470
Base [KOPiv]	-2,316728	4,412384	-0,53	0,6034
Base eq. (1,3)	6,8120073	3,891001	1,75	0,0902
Supporting electrolyte [$LiClO_4$]	4,4092628	3,465246	1,27	0,2130
Supporting electrolyte [NBu_4BF_4]	-6,142688	3,579876	-1,72	0,0965
Supporting electrolyte [NEt_4OTs]	1,7334257	4,125438	0,42	0,6773
Electrolyte concentration (0,1,0,3)	-0,245166	2,850444	-0,09	0,9320
Temperature (25,55)	-7,553247	3,377447	-2,24	0,0329*
Applied charge (1,8,2,4)	-1,006237	2,847243	-0,35	0,7263
Charge density (5,10)	-0,828025	3,350017	-0,25	0,8065
Anode material [Platinum]	5,6378359	3,543543	1,59	0,1221
Anode material [Graphite]	-3,844954	4,083495	-0,94	0,3539
Anode material [Glassy carbon]	-1,792882	3,907152	-0,46	0,6496
Cathode material [Platinum]	0,2383677	3,741085	0,06	0,9496
Cathode material [Graphite]	0,3994459	3,611149	0,11	0,9127
Cathode material [Glassy carbon]	-0,637814	3,6065	-0,18	0,8608
Iodine loading × Iodine loading	6,4520772	6,459905	1,00	0,3259
Base eq. × Base eq.	-3,806005	6,324798	-0,60	0,5519

Term	Scaled Estimate	Std Error	t Ratio	Prob>|t|
Electrolyte concentration× Electrolyte concentration	14,6176	5,677999	2,57	0,0152*
Temperature × Temperature	1,0801233	5,720539	0,19	0,8515
Applied charge × Applied charge	2,9829535	5,538589	0,54	0,5942
Charge density × Charge density	-3,570511	5,948768	-0,60	0,5529
Supporting electrolyte [$LiClO_4$] × Base [Lutidine]	16,683554	6,300052	2,65	0,0128*
Supporting electrolyte [$LiClO_4$] × Base [DBU]	-26,34633	7,2219	-3,65	0,0010*
Supporting electrolyte [$LiClO_4$] × Base [Pyridine]	0,4651916	6,606032	0,07	0,9443
Supporting electrolyte [$LiClO_4$] × Base [KOPiv]	9,1975804	6,793664	1,35	0,1859
Supporting electrolyte [NBu_4BF_4] × Base [Lutidine]	-28,95644	6,015339	-4,81	<,0001*
Supporting electrolyte [NBu_4BF_4] × Base [DBU]	22,178629	7,470549	2,97	0,0058*
Supporting electrolyte [NBu_4BF_4] × Base [Pyridine]	-1,282876	7,703434	-0,17	0,8689
Supporting electrolyte [NBu_4BF_4] × Base [KOPiv]	8,0606901	6,531052	1,23	0,2267
Supporting electrolyte [NEt_4OTs] × Base [Lutidine]	12,27289	5,895988	2,08	0,0460*
Supporting electrolyte [NEt_4OTs] × Base [DBU]	4,1676961	8,133789	0,51	0,6121
Supporting electrolyte [NEt_4OTs] × Base [Pyridine]	0,8176847	6,647613	0,12	0,9029
Supporting electrolyte [NEt_4OTs] × Base [KOPiv]	-17,25827	7,330847	-2,35	0,0253*

Prediction Profiler

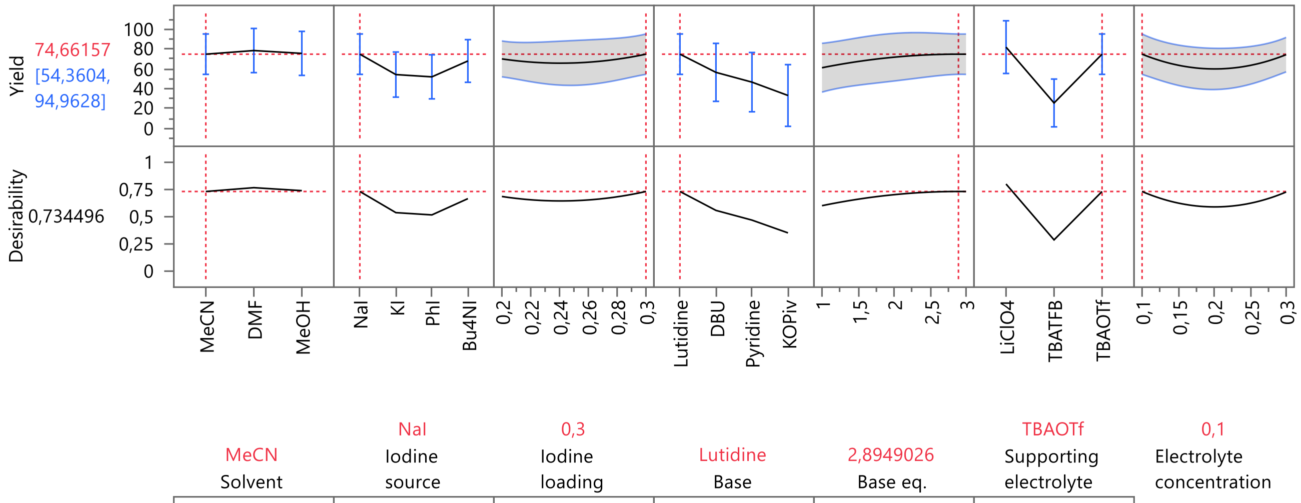

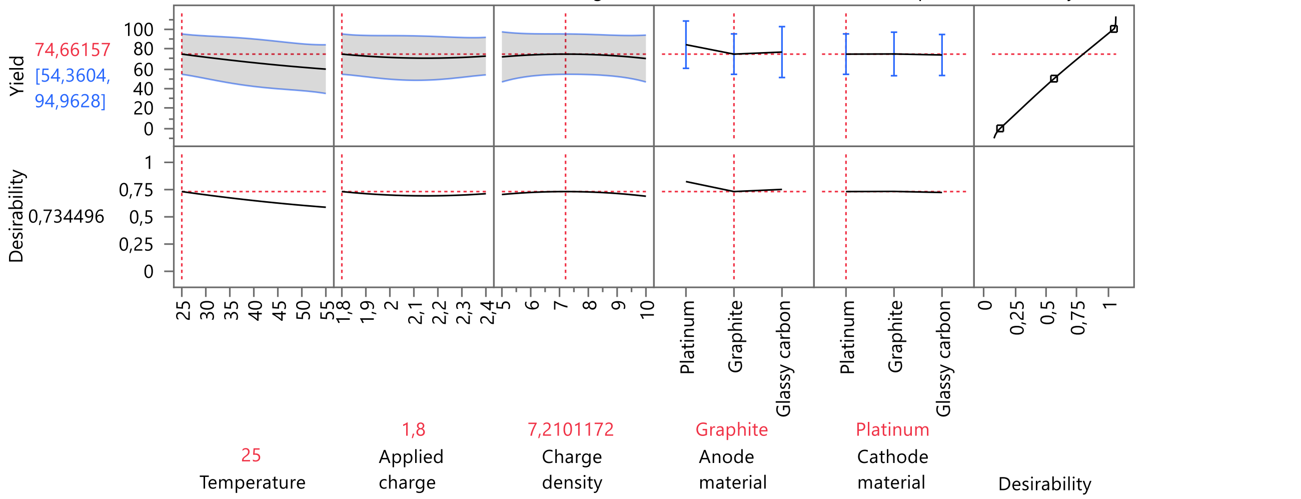

Sensitivity assessment

In a H-type cell, both compartments were charged with NEt_4OTs (723 mg, 4.80 eq, 2.40 mmol) in acetonitrile. Sodium iodide (22.4 mg, 0.30 eq., 0.15 mmol), 2,6-lutidine (130 mg, 2.40 eq., 1.20 mmol) and enamine **1b** (126 mg, 1.00 eq., 0.50 mmol) were added to the anode compartment. The cathode compartment was equipped with platinum electrode, the anode compartment was equipped with a graphite electrode. The reaction was electrolyzed under constant current at room temperature for 3 h 13 min (2.4 $F{\cdot}mol^{-1}$). The yields were determined by ^{19}F NMR using *2*-nitro-fluorobenzene (52.6 μL, 1.00 eq., 0.50 mmol) as internal standard.

Table 5: Sensitivity assessment. All reactions were carried out on a 0.5 mmol scale using substrate **2b**.

Parameters		Usual reaction conditions	
	high	*87%*	low
**Concentration**	V_{rxn} – 10% V_{rxn}<br>9 mL per compartment	*V_{rxn} = 10 mL per compartment*	V_{rxn} + 10% V_{rxn}<br>11 mL per compartment
**H_2O level**	HPLC grade acetonitrile + 10 eq. H_2O	*HPLC grade acetonitril*	dry acetonitrile over molecular sieve 3Å
**O_2 level**	O_2 atmosphere	atmospheric conditions	Argon atmosphere
**Current density**	12 $mA{\cdot}cm^{-2}$	10 $mA{\cdot}cm^{-2}$	8 $mA{\cdot}cm^{-2}$
**Distance of electrodes**	–	*6.5 cm*	7.5 cm
**Electrode surface**	1.0 × 3.0 cm	*1.7 × 3.0 cm*	–

Table 6: Outcome of the sensitivity assessment. The yields were determined by ^{19}F NMR spectroscopy using 2-nitro-fluorobenzene as the internal standard.

	Yield [%]	Yield of Reference [%]	Deviation
Reference		87	
Low concentration	84		-3
High concentration	85		-2
Low H_2O	79		-8
High H_2O	87		0
Low O_2	80		-7
High O_2	72		-15
Low current density	88		1
High current density	78		-9
Distance of electrodes	81		-6
Small electrodes surface	76		-11

Table 7: Table of content for radar diagram.

	Deviation [%]	Dark red ring	Red ring	Rose ring	White ring	Bright green	Dark green ring
Low concentration	-3	-87.5	-62.5	-37.5	-12.5	12.5	37.5
High concentration	-2	-87.5	-62.5	-37.5	-12.5	12.5	37.5
Low H_2O	-8	-87.5	-62.5	-37.5	-12.5	12.5	37.5
High H_2O	-2	-87.5	-62.5	-37.5	-12.5	12.5	37.5
Low O_2	-7	-87.5	-62.5	-37.5	-12.5	12.5	37.5
High O_2	-15	-87.5	-62.5	-37.5	-12.5	12.5	37.5
Low current density	1	-87.5	-62.5	-37.5	-12.5	12.5	37.5
High current density	-9	-87.5	-62.5	-37.5	-12.5	12.5	37.5
Distance of electrodes	-6	-87.5	-62.5	-37.5	-12.5	12.5	37.5
Small electrodes surface	-11	-87.5	-62.5	-37.5	-12.5	12.5	37.5

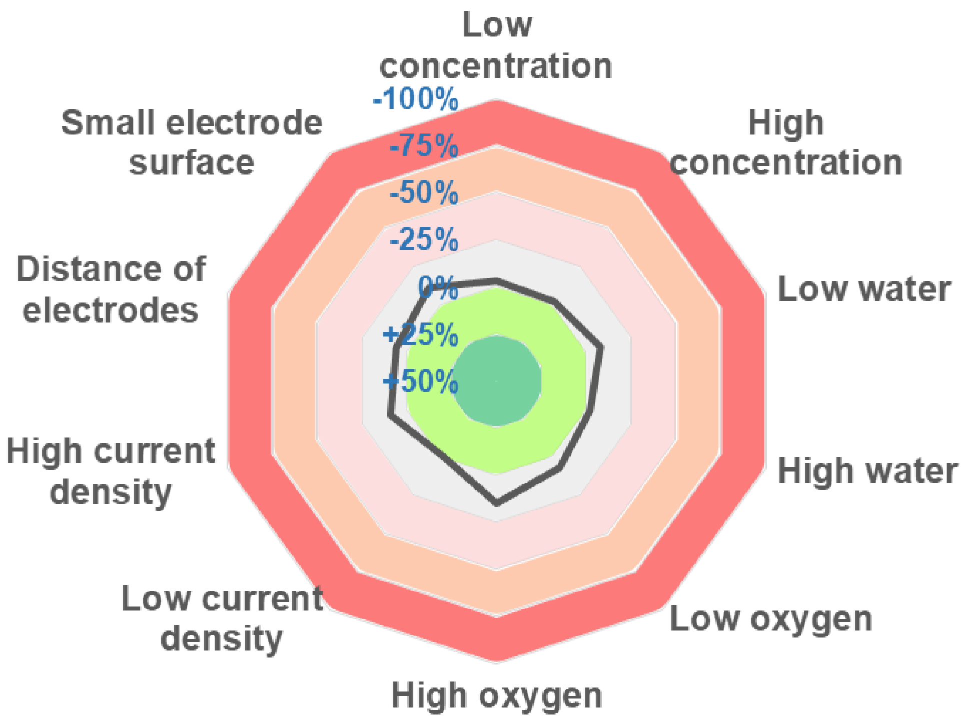

Figure 8: Radar diagram of electrochemical iodine-mediated oxidation of enamino-esters to 2*H*-azirine-2-carboxylates.

Robustness screen

In a H-type cell, both compartments were charged with NEt_4OTs (723 mg, 4.80 eq, 2.40 mmol) in acetonitrile (each compartment 10 mL). Sodium iodide (22.4 mg, 0.30 eq., 0.15 mmol), 2,6-lutidine (130 mg, 2.40 eq., 1.20 mmol), enamine **1b** (126 mg, 1.00 eq., 0.50 mmol) and respective additive (1.00 eq., 0.50 mmol) were added to the anode compartment. The cathode compartment was equipped with a platinum electrode, the anode compartment was equipped with a graphite electrode. The reaction was electrolyzed under constant current (10 $mA{\cdot}cm^{-2}$) at room temperature for 3 h 13 (2.4 $F{\cdot}mol^{-1}$). A stock solution of 2-nitro-fluorobenzene (70.6 mg, 1.00 eq., 0.50 mmol) and *n*-dodecane (85.2 mg, 1.00 eq., 0.50 mmol) in chloroform was added to the anode compartment. The yields were determined with ^{19}F NMR and GC-FID.

Table 8: Functional group tolerance test with screening substrate **1b**.[a]

	Additve	Yield starting material [%][b]	Yield product [%][b]	Yield additive [%][c]
	**no additive**	**0**	**87**	**–**
**1**	2-chlorochinoline	3	**69**	**>95**
**2**	benzaldehyde	2	**73**	**49**
**3**	phenol	78	**1**	**39**
**4**	1-dodecyne	10	**70**	**56**
**5**	acetanilide	0	**53**	**32**
**6**	1-chlorooctane	2	**69**	**>95**
**7**	dodecylamine	15	**72**	**3**
**8**	sulfolane	3	**70**	**0**
**9**	1-octene	17	**57**	**55**
**10**	benzothiazole	0	**67**	**58**
**11**	*N*-methyl indole	89	**1**	**0**
**12**	1-N-Boc-2-piperidone	2	**72**	**0**
**13**	benzonitrile	2	**73**	**67**
**14**	1,2-epoxyoctane	10	**76**	**61**
**15**	carbazole	70	**1**	**40**
**16**	*N*-benzyl pyrrole	76	**0**	**19**
**17**	benzothiophene	9	**71**	**0**
**18**	2-methyl anisole	4	**69**	**52**
**19**	2-vinyl naphthalene	15	**62**	**12**
**20**	valerophenone	2	**76**	**58**
**21**	1-butanol	2	**77**	**49**
**22**	cyclopropyl benzene	0	**67**	**61**

[a] All reactions were carried out on a 0.5 mmol scale. [b] Yields were determined by ^{19}F NMR spectroscopy using *2*-nitro-fluorobenzene as the internal standard. [c] The yields were determined by GC-FID analysis using mesitylene as the internal standard.

Cyclic voltammetry

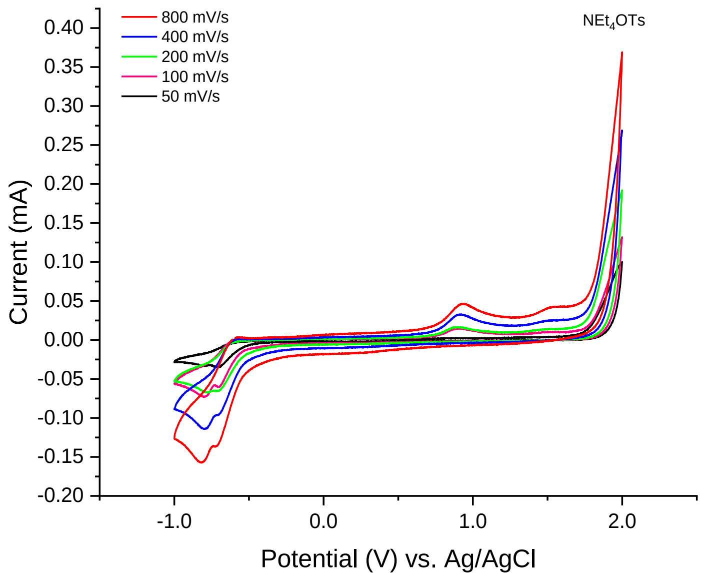

Figure 9: Cyclic voltammetry of NEt_4OTs (0.24 mol·L^{-1}) in acetonitrile (10 mL) with glassy carbon anode, platinum wire as cathode and Ag/AgCl as reference electrode (3 mol·L^{-1} NaCl solution). Switching potential 1: 2.00 V, switching potential 2: -1.00 V.

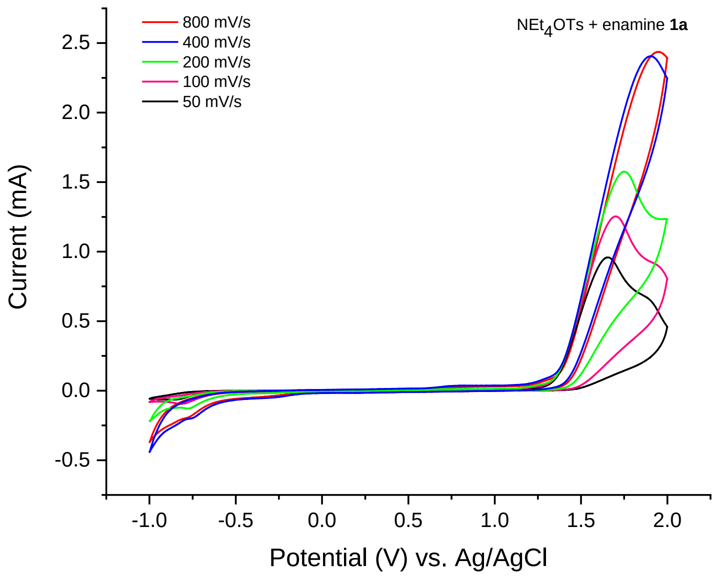

Figure 10: Cyclic voltammetry of NEt_4OTs (0.24 mol·L^{-1}) and enamine **1a** (0.050 mol·L^{-1}) in acetonitrile (10 mL) with glassy carbon anode, platinum wire as cathode and Ag/AgCl as reference electrode (3 mol·L^{-1} NaCl solution). Switching potential 1: 2.00 V, switching potential 2: -1.00 V.

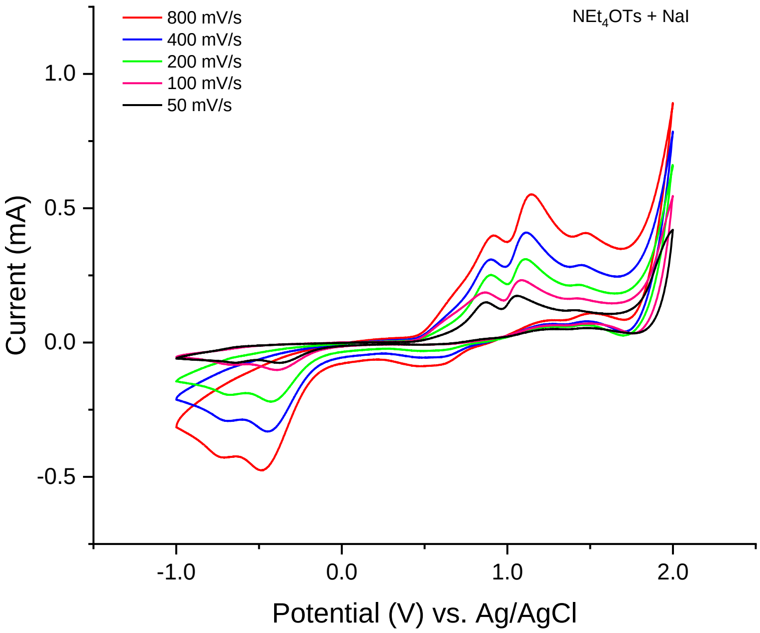

Figure 11: Cyclic voltammetry of NEt_4OTs (0.24 mol·L^{-1}) and sodium iodide (0.015 mol·L^{-1}) in acetonitrile (10 mL) with glassy carbon anode, platinum wire as cathode and Ag/AgCl as reference electrode (3 mol·L^{-1} NaCl solution). Switching potential 1: 2.00 V, switching potential 2: -1.00 V.

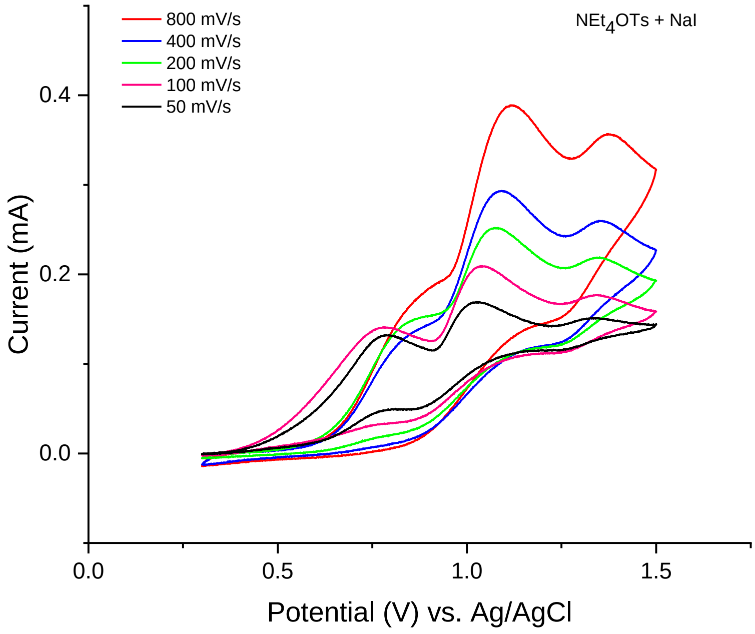

Figure 12: Cyclic voltammetry of NEt_4OTs (0.24 mol·L^{-1}) and sodium iodide (0.015 mol·L^{-1}) in acetonitrile (10 mL) with glassy carbon anode, platinum wire as cathode and Ag/AgCl as reference electrode (3 mol·L^{-1} NaCl solution). Switching potential 1: 1.50 V, switching potential 2: 0.30 V.

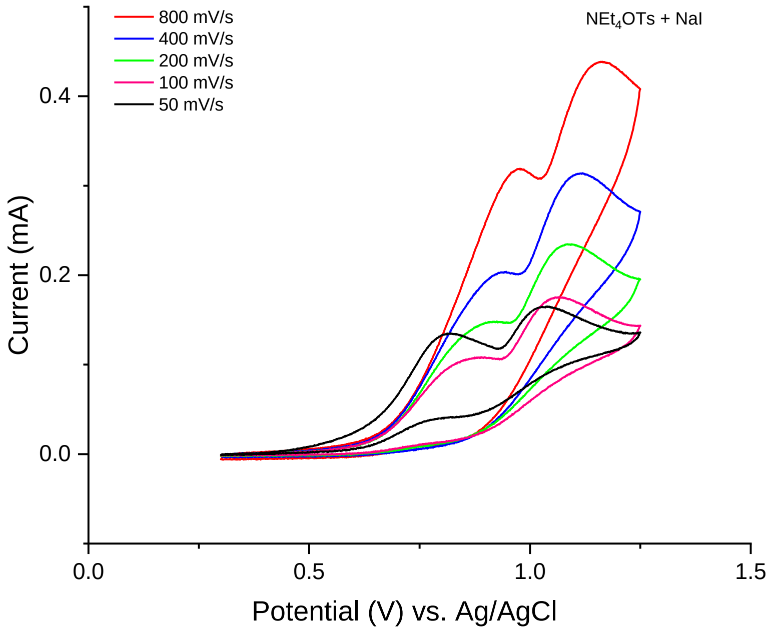

Figure 13: Cyclic voltammetry of NEt_4OTs (0.24 mol·L^{-1}) and sodium iodide (0.015 mol·L^{-1}) in acetonitrile (10 mL) with glassy carbon anode, platinum wire as cathode and Ag/AgCl as reference electrode (3 mol·L^{-1} NaCl solution). Switching potential 1: 1.25 V, switching potential 2: 0.30 V.

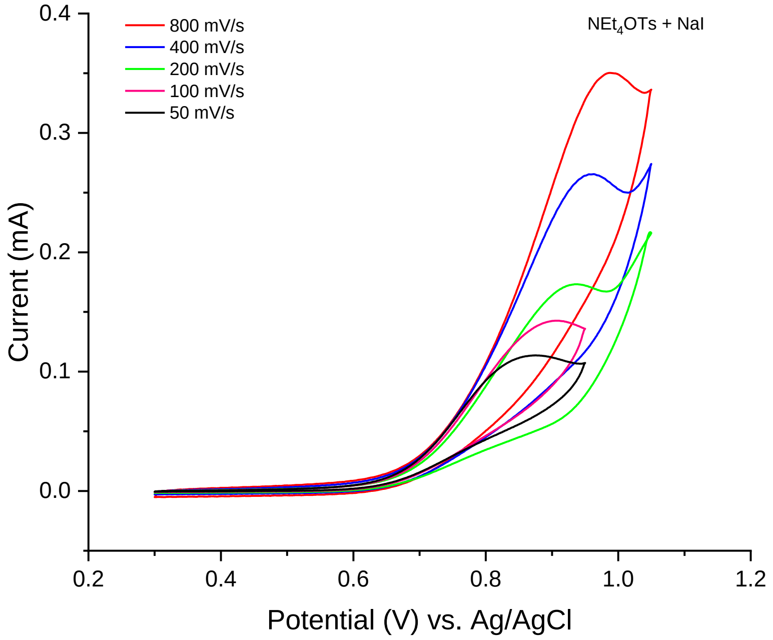

Figure 14: Cyclic voltammetry of NEt_4OTs (0.24 mol·L^{-1}) and sodium iodide (0.015 mol·L^{-1}) in acetonitrile (10 mL) with glassy carbon anode, platinum wire as cathode and Ag/AgCl as reference electrode (3 mol·L^{-1} NaCl solution). Switching potential 1: 0.95 V, switching potential 2: 0.30 V.

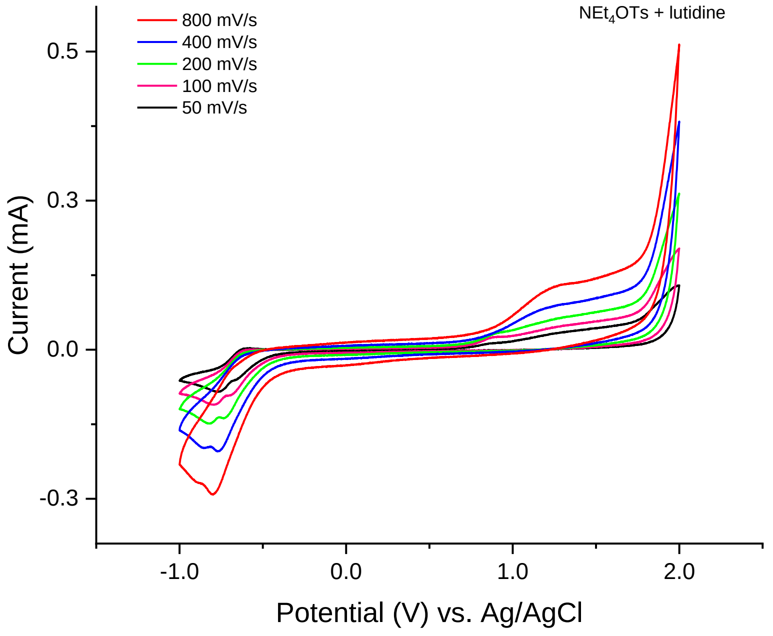

Figure 15: Cyclic voltammetry of NEt_4OTs (0.24 mol·L^{-1}) and lutidine (0.150 mol·L^{-1}) in acetonitrile (10 mL) with glassy carbon anode, platinum wire as cathode and Ag/AgCl as reference electrode (3 mol·L^{-1} NaCl solution). Switching potential 1: 2.00 V, switching potential 2: -1.00 V.

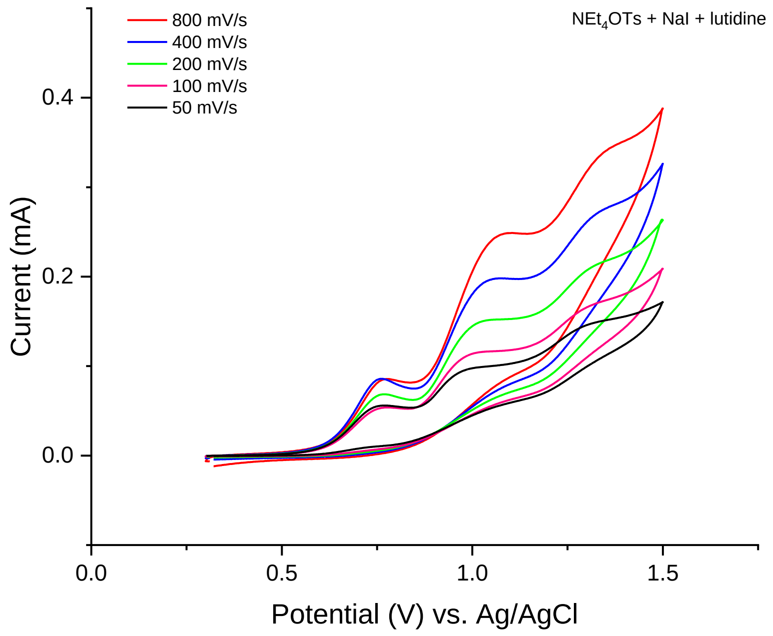

Figure 16: Cyclic voltammetry of NEt_4OTs (0.24 mol·L^{-1}), sodium iodide (0.015 mol·L^{-1}), and lutidine (0.150 mol·L^{-1}) in acetonitrile (10 mL) with glassy carbon anode, platinum wire as cathode and Ag/AgCl as reference electrode (3 mol·L^{-1} NaCl solution). Switching potential 1: 1.50 V, switching potential 2: 0.30 V.

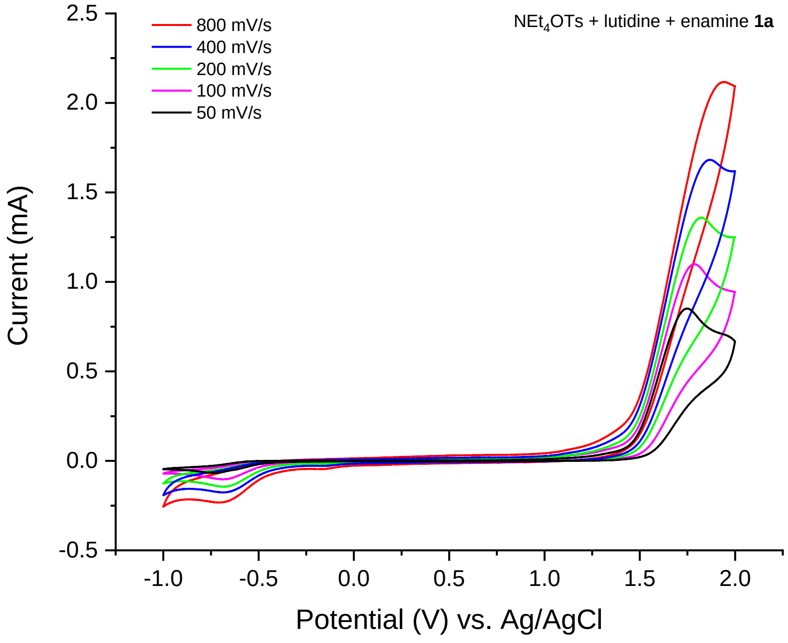

Figure 17: Cyclic voltammetry of NEt_4OTs (0.24 mol·L^{-1}), lutidine (0.150 mol·L^{-1}), and enamine **1a** (0.050 mol·L^{-1}) in acetonitrile (10 mL) with glassy carbon anode, platinum wire as cathode and Ag/AgCl as reference electrode (3 mol·L^{-1} NaCl solution). Switching potential 1: 2.00 V, switching potential 2: -1.00 V.

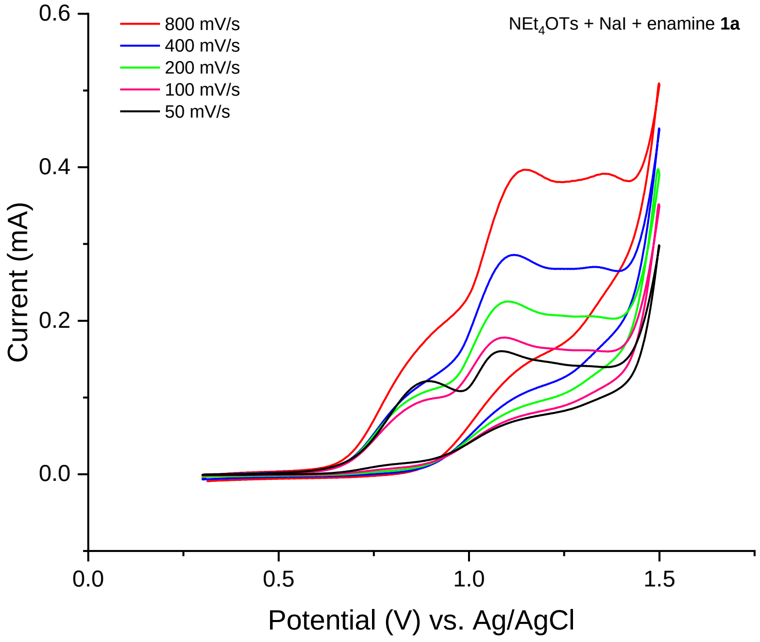

Figure 18: Cyclic voltammetry of NEt_4OTs (0.24 mol·L^{-1}), sodium iodide (0.015 mol·L^{-1}), and enamine **1a** (0.050 mol·L^{-1}) in acetonitrile (10 mL) with glassy carbon anode, platinum wire as cathode and Ag/AgCl as reference electrode (3 mol·L^{-1} NaCl solution). Switching potential 1: 1.50 V, switching potential 2: 0.30 V.

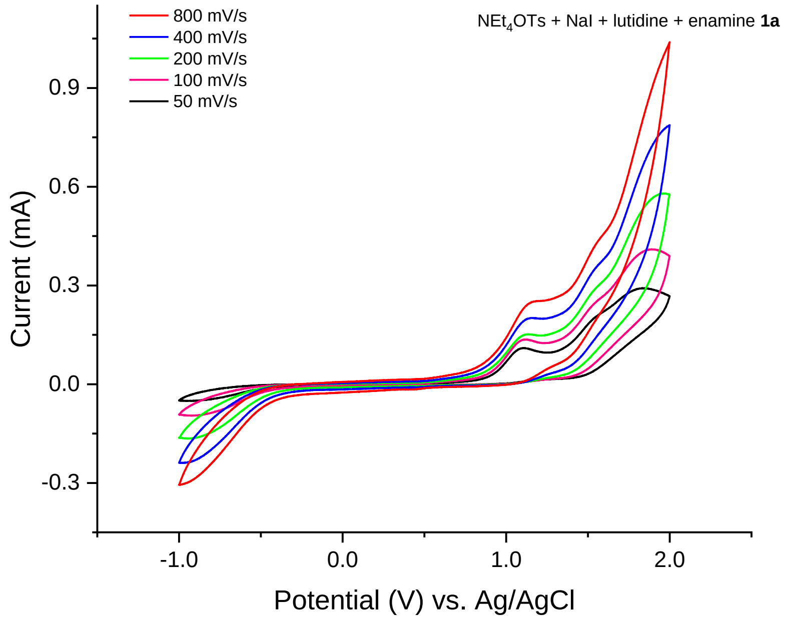

Figure 19: Cyclic voltammetry of NEt_4OTs (0.24 mol·L^{-1}), sodium iodide (0.015 mol·L^{-1}), lutidine (0.150 mol·L^{-1}), and enamine **1a** (0.050 mol·L^{-1}) in acetonitrile (10 mL) with glassy carbon anode, platinum wire as cathode and Ag/AgCl as reference electrode (3 mol·L^{-1} NaCl solution). Switching potential 1: 2.00 V, switching potential 2: -1.00 V.

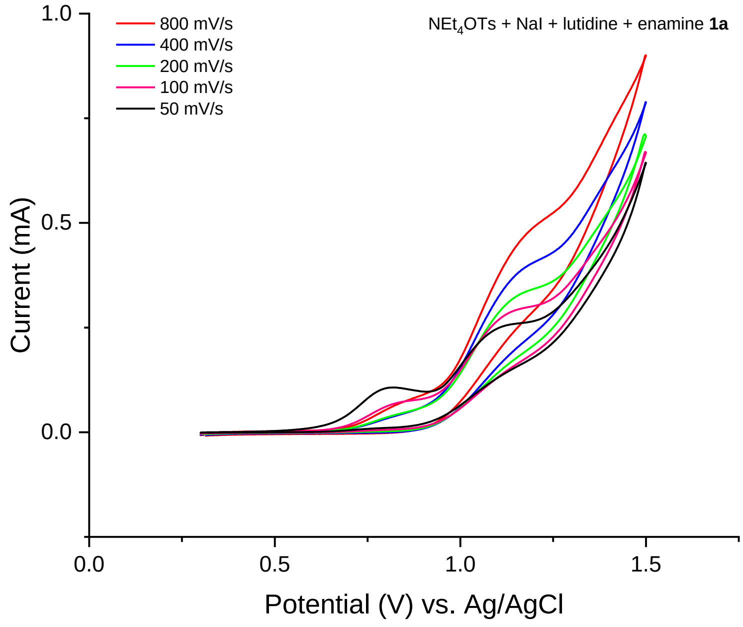

Figure 20: Cyclic voltammetry of NEt_4OTs (0.24 mol·L^{-1}), sodium iodide (0.015 mol·L^{-1}), lutidine (0.150 mol·L^{-1}), and enamine **1a** (0.050 mol·L^{-1}) in acetonitrile (10 mL) with glassy carbon anode, platinum wire as cathode and Ag/AgCl as reference electrode (3 mol·L^{-1} NaCl solution). Switching potential 1: 1.50 V, switching potential 2: 0.30 V.

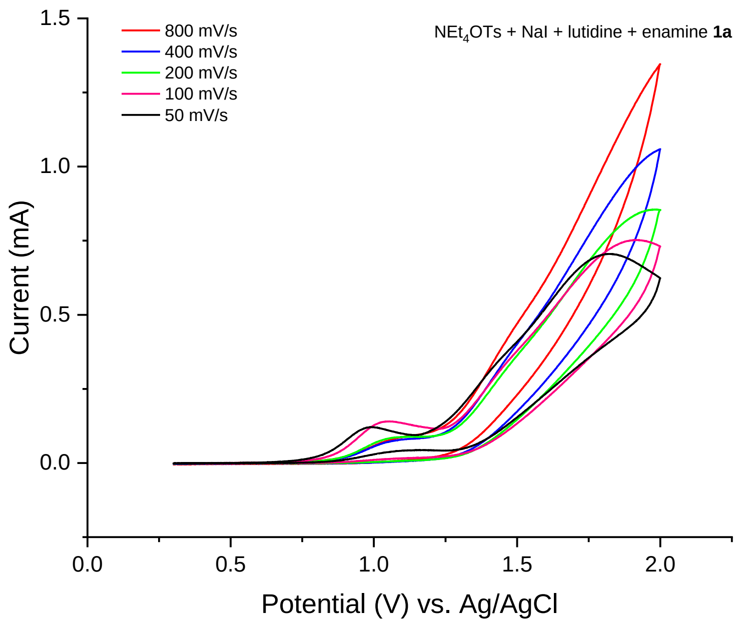

Figure 21: Cyclic voltammetry of NEt4OTs (0.24 mol·L^{-1}), sodium iodide (0.015 mol·L^{-1}), lutidine (0.150 mol·L^{-1}), and enamine **1a** (0.050 mol·L^{-1}) in acetonitrile (10 mL) with glassy carbon anode, platinum wire as cathode and Ag/AgCl as reference electrode (3 mol·L^{-1} NaCl solution). Switching potential 1: 2.00 V, switching potential 2: 0.30 V.

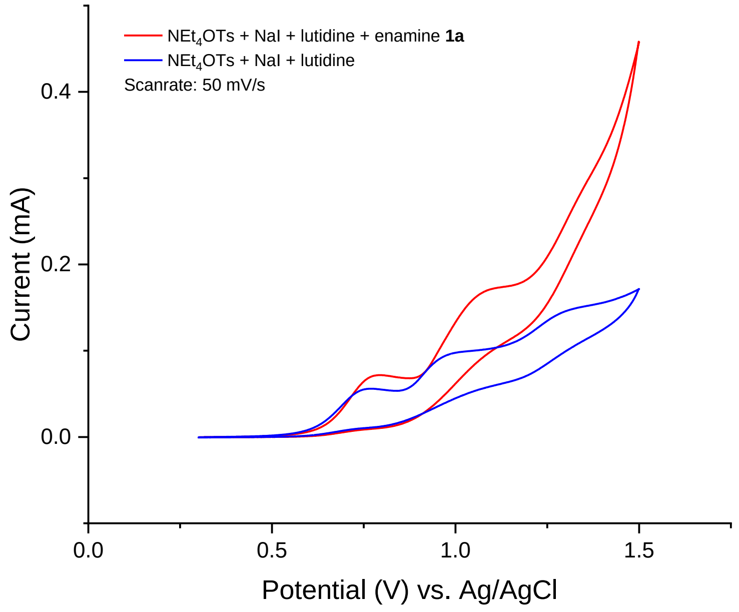

Figure 22: Cyclic voltammetry of NEt4OTs (0.24 mol·L^{-1}), sodium iodide (0.015 mol·L^{-1}), lutidine (0.150 mol·L^{-1}), and enamine **1a** (0.050 mol·L^{-1}) in acetonitrile (10 mL) with glassy carbon anode, platinum wire as cathode and Ag/AgCl as reference electrode (3 mol·L^{-1} NaCl solution). Switching potential 1: 1.50 V, switching potential 2: 0.30 V.

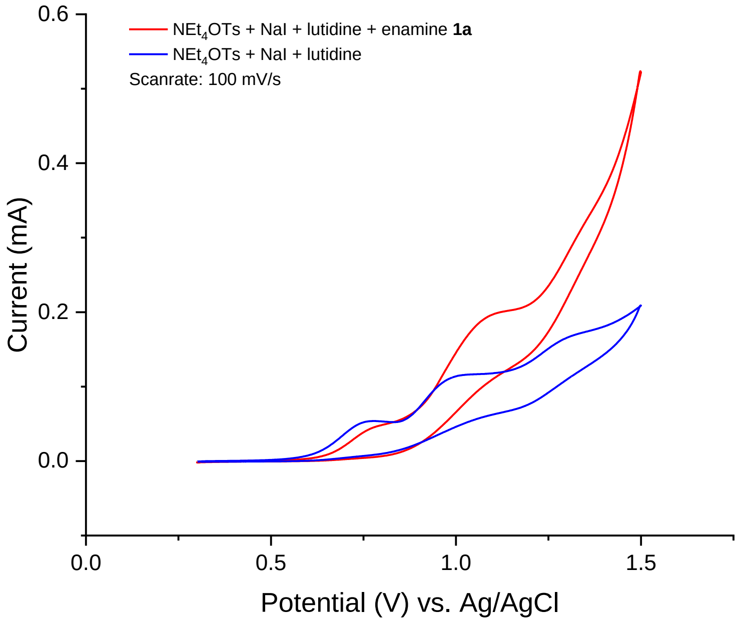

Figure 23: Cyclic voltammetry of NEt_4OTs (0.24 mol·L^{-1}), sodium iodide (0.015 mol·L^{-1}), lutidine (0.150 mol·L^{-1}), and enamine **1a** (0.050 mol·L^{-1}) in acetonitrile (10 mL) with glassy carbon anode, platinum wire as cathode and Ag/AgCl as reference electrode (3 mol·L^{-1} NaCl solution). Switching potential 1: 1.50 V, switching potential 2: 0.30 V.

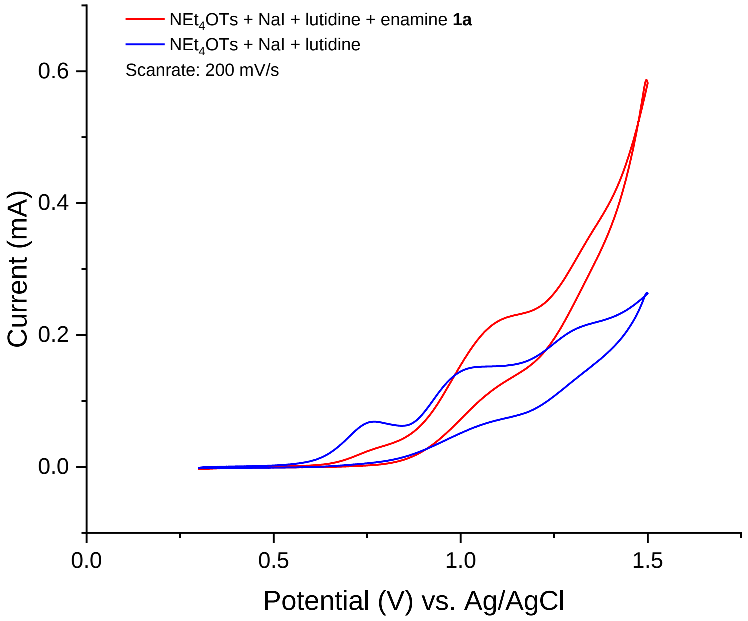

Figure 24: Cyclic voltammetry of NEt_4OTs (0.24 mol·L^{-1}), sodium iodide (0.015 mol·L^{-1}), lutidine (0.150 mol·L^{-1}), and enamine **1a** (0.050 mol·L^{-1}) in acetonitrile (10 mL) with glassy carbon anode, platinum wire as cathode and Ag/AgCl as reference electrode (3 mol·L^{-1} NaCl solution). Switching potential 1: 1.50 V, switching potential 2: 0.30 V.

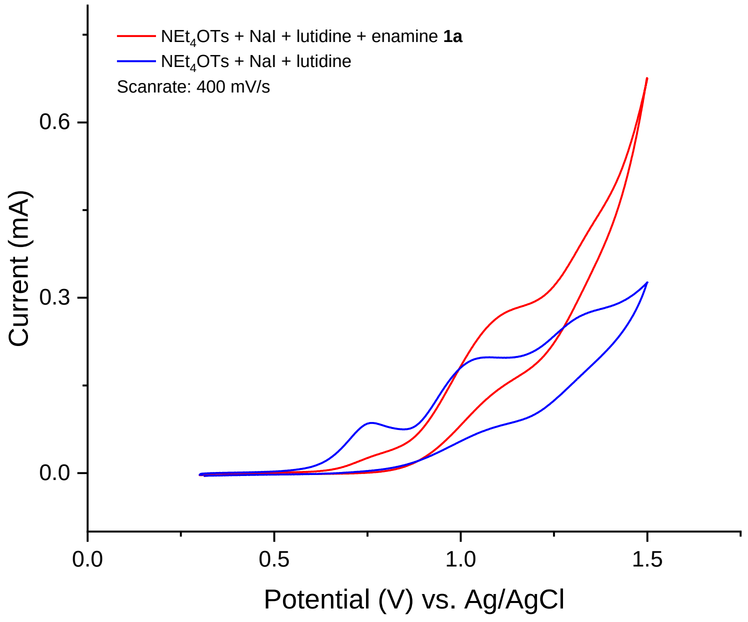

Figure 25: Cyclic voltammetry of NEt_4OTs (0.24 mol·L^{-1}), sodium iodide (0.015 mol·L^{-1}), lutidine (0.150 mol·L^{-1}), and enamine **1a** (0.050 mol·L^{-1}) in acetonitrile (10 mL) with glassy carbon anode, platinum wire as cathode and Ag/AgCl as reference electrode (3 mol·L^{-1} NaCl solution). Switching potential 1: 1.50 V, switching potential 2: 0.30 V.

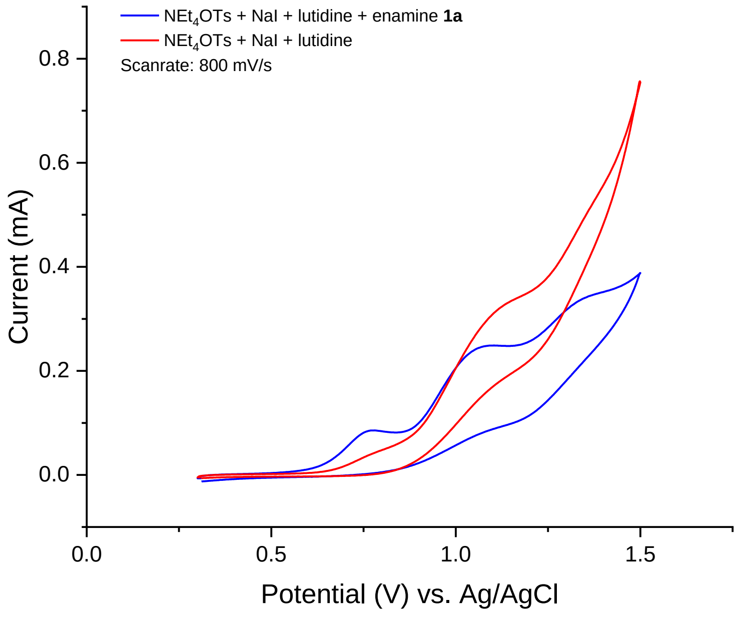

Figure 26: Cyclic voltammetry of NEt_4OTs (0.24 mol·L^{-1}), sodium iodide (0.015 mol·L^{-1}), lutidine (0.150 mol·L^{-1}), and enamine **1a** (0.050 mol·L^{-1}) in acetonitrile (10 mL) with glassy carbon anode, platinum wire as cathode and Ag/AgCl as reference electrode (3 mol·L^{-1} NaCl solution). Switching potential 1: 1.50 V, switching potential 2: 0.30 V.

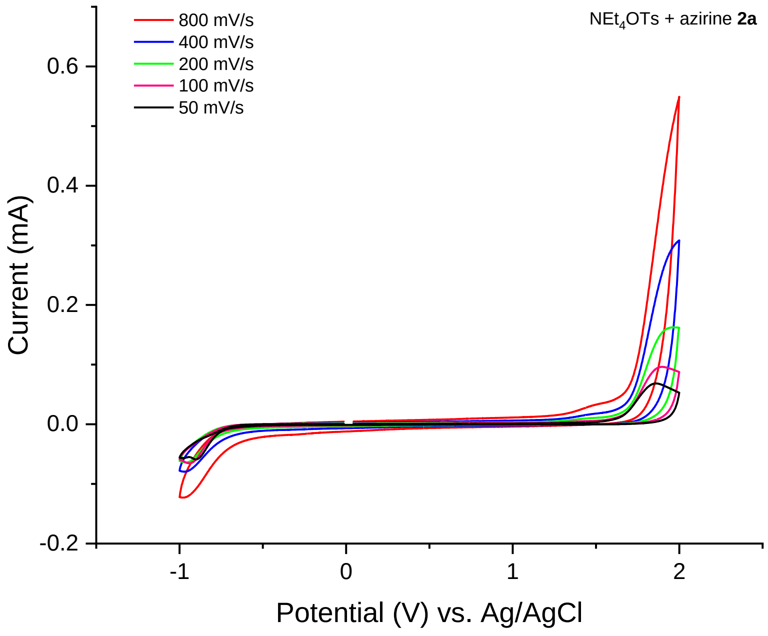

Figure 27: Cyclic voltammetry of NEt_4OTs (0.24 mol·L^{-1}) and azirine **2a** (0.050 mol·L^{-1}) in acetonitrile (10 mL) with glassy carbon anode, platinum wire as cathode and Ag/AgCl as reference electrode (3 mol·L^{-1} NaCl solution). Switching potential 1: 2.00 V, switching potential 2: -1.00 V.

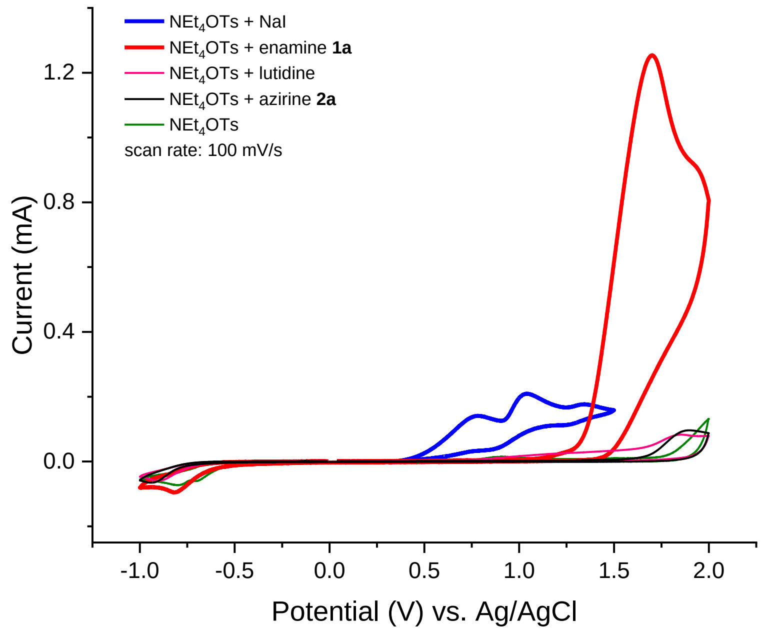

Figure 28: Cyclic voltammetry of NEt_4OTs (0.24 mol·L^{-1}), sodium iodide (0.015 mol·L^{-1}), lutidine (0.150 mol·L^{-1}), enamine **1a** (0.050 mol·L^{-1}), and azirine (0.050 mol·L^{-1}) in acetonitrile (10 mL) with glassy carbon anode, platinum wire as cathode and Ag/AgCl as reference electrode (3 mol·L^{-1} NaCl solution). Switching potential 1: 2.00 V, switching potential 2: -1.00 V.

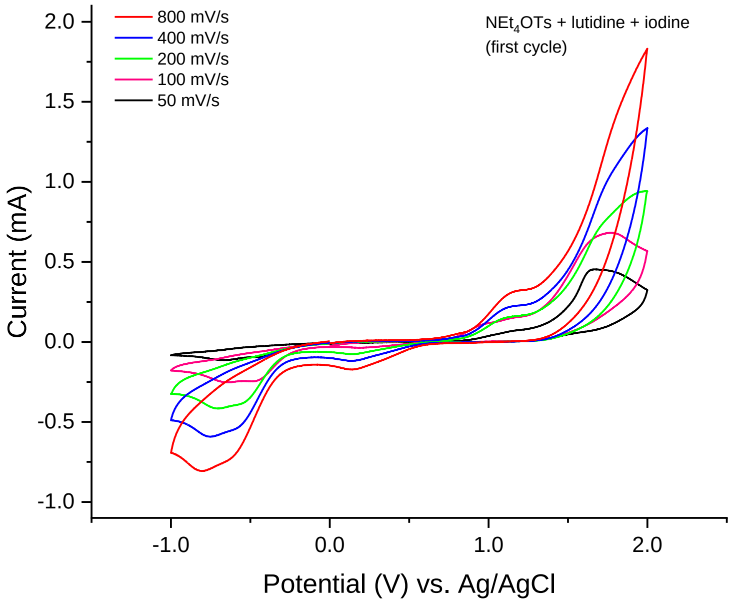

Figure 29: Cyclic voltammetry of NEt_4OTs (0.24 mol·L^{-1}), lutidine (0.150 mol·L^{-1}), and iodine (0.015 mol·L^{-1}) in acetonitrile (10 mL) with glassy carbon anode, platinum wire as cathode and Ag/AgCl as reference electrode (3 mol·L^{-1} NaCl solution). Switching potential 1: 2.00 V, switching potential 2: 1.00 V, number of segments: 3 (first cycle).

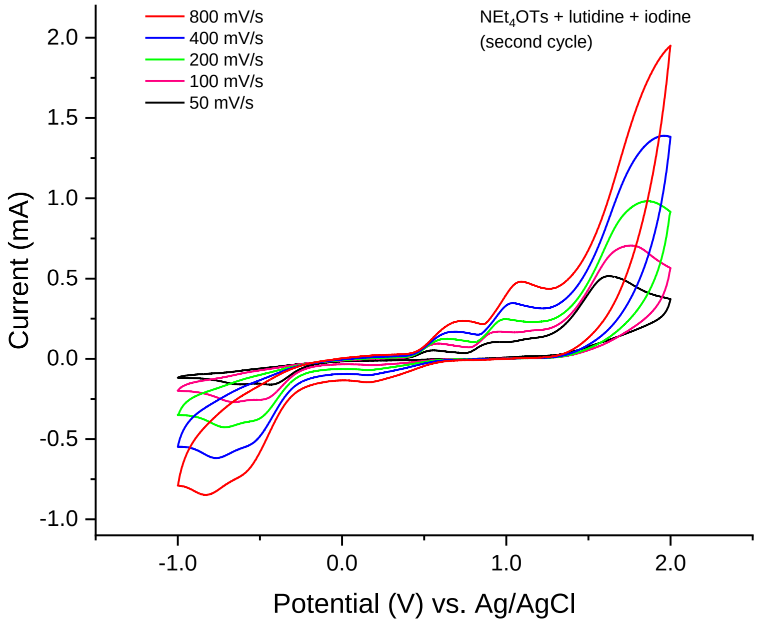

Figure 30: Cyclic voltammetry of NEt_4OTs (0.24 mol·L^{-1}), lutidine (0.150 mol·L^{-1}), and iodine (0.015 mol·L^{-1}) in acetonitrile (10 mL) with glassy carbon anode, platinum wire as cathode and Ag/AgCl as reference electrode (3 mol·L^{-1} NaCl solution). Switching potential 1: 2.00 V, switching potential 2: 1.00 V, number of segments: 6 (second cycle).

NMR spectra

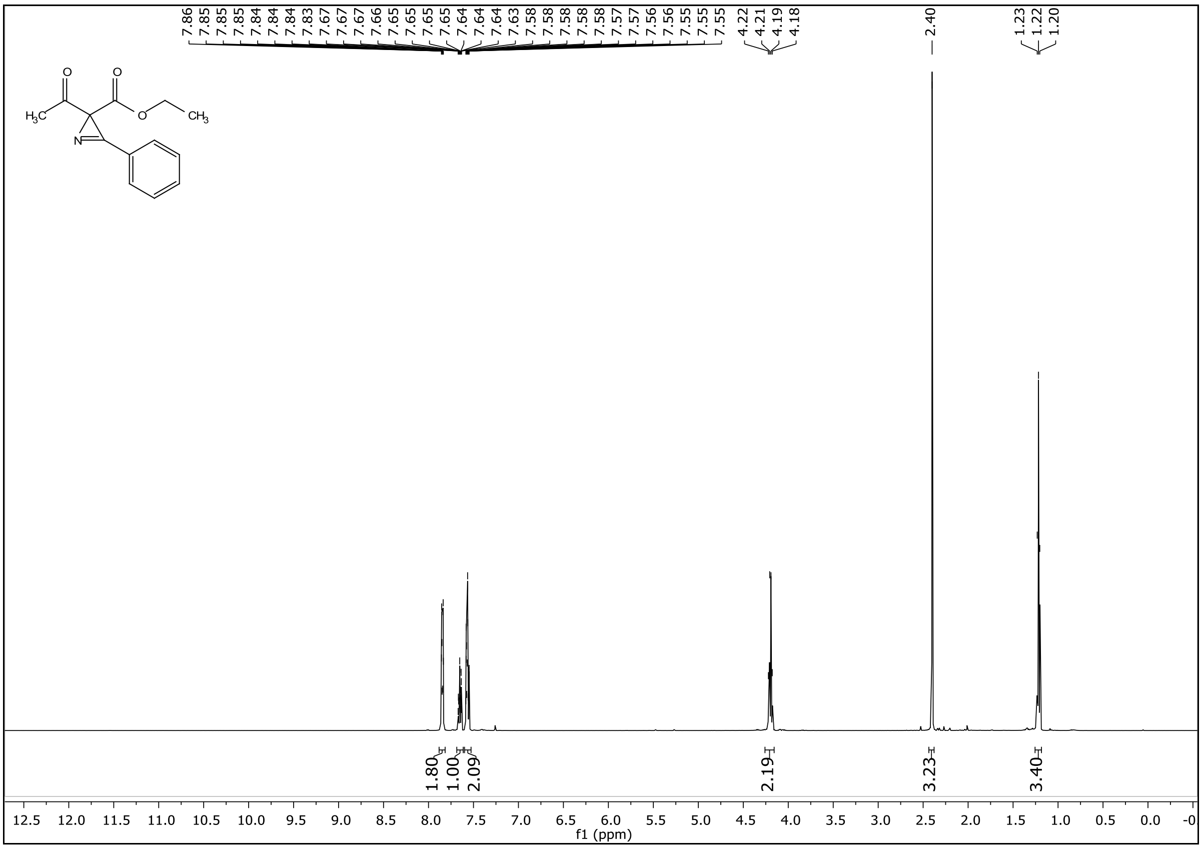
7.86
7.85
7.85
7.85
7.84
7.84
7.84
7.83
7.67
7.67
7.67
7.66
7.65
7.65
7.65
7.65
7.64
7.64
7.64
7.63
7.58
7.58
7.58
7.58
7.58
7.57
7.56
7.56
7.55
7.55
7.55
4.22
4.21
4.19
4.18
2.40
1.23
1.22
1.20
1.80
1.00
2.09
2.19
3.23
3.40
f1 (ppm)

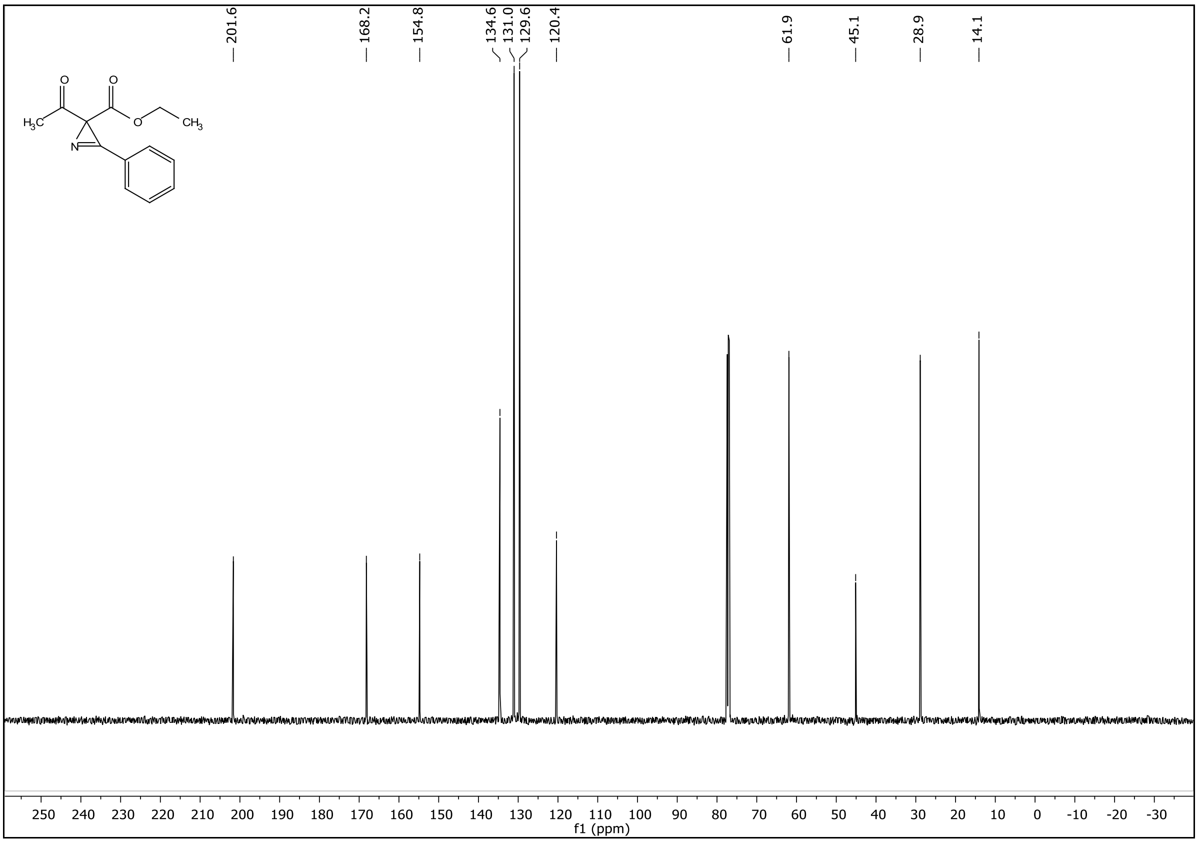
201.6
168.2
154.8
134.6
131.0
129.6
120.4
61.9
45.1
28.9
14.1
f1 (ppm)

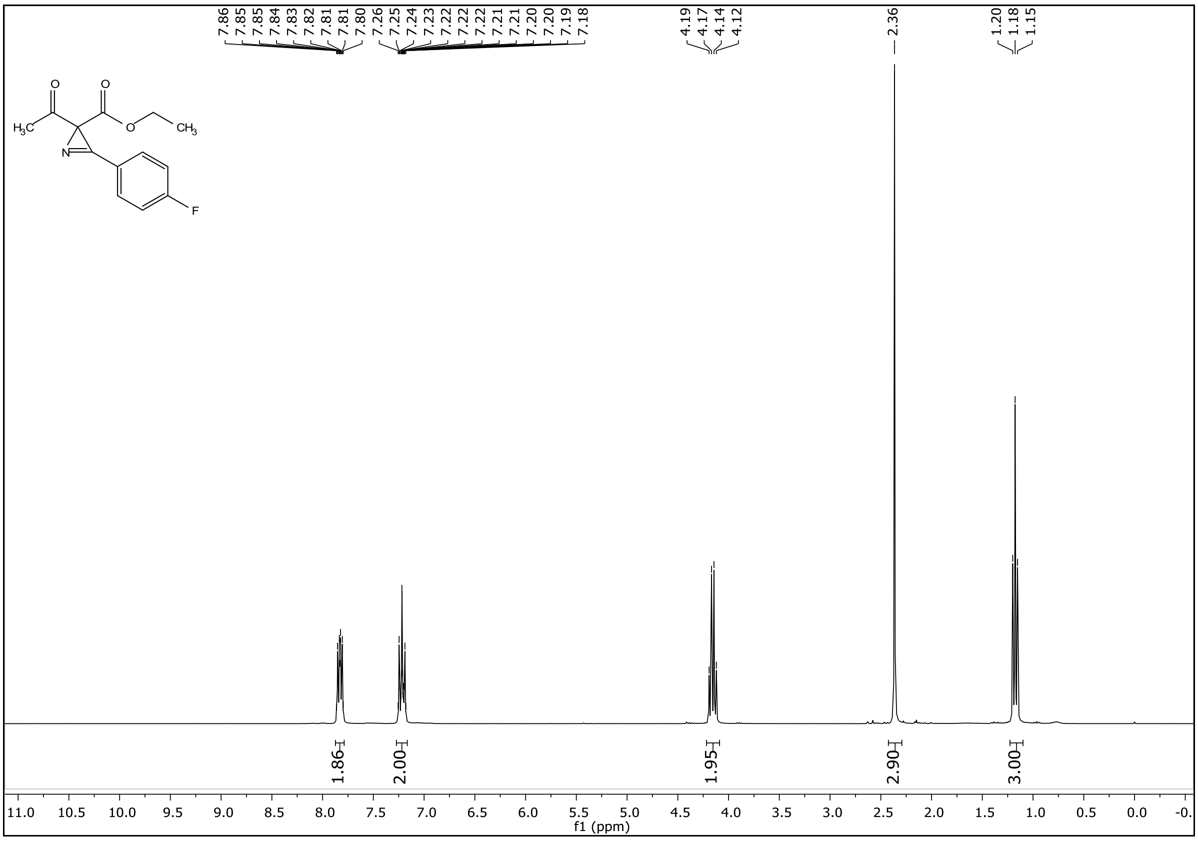
7.86
7.85
7.85
7.84
7.83
7.82
7.81
7.81
7.80
7.26
7.25
7.24
7.23
7.22
7.22
7.22
7.21
7.21
7.20
7.20
7.19
7.18
4.19
4.17
4.14
4.12
2.36
1.20
1.18
1.15
O
O
H3C
O
CH3
N
F
1.86
2.00
1.95
2.90
3.00
11.0 10.5 10.0 9.5 9.0 8.5 8.0 7.5 7.0 6.5 6.0 5.5 5.0 4.5 4.0 3.5 3.0 2.5 2.0 1.5 1.0 0.5 0.0
f1 (ppm)

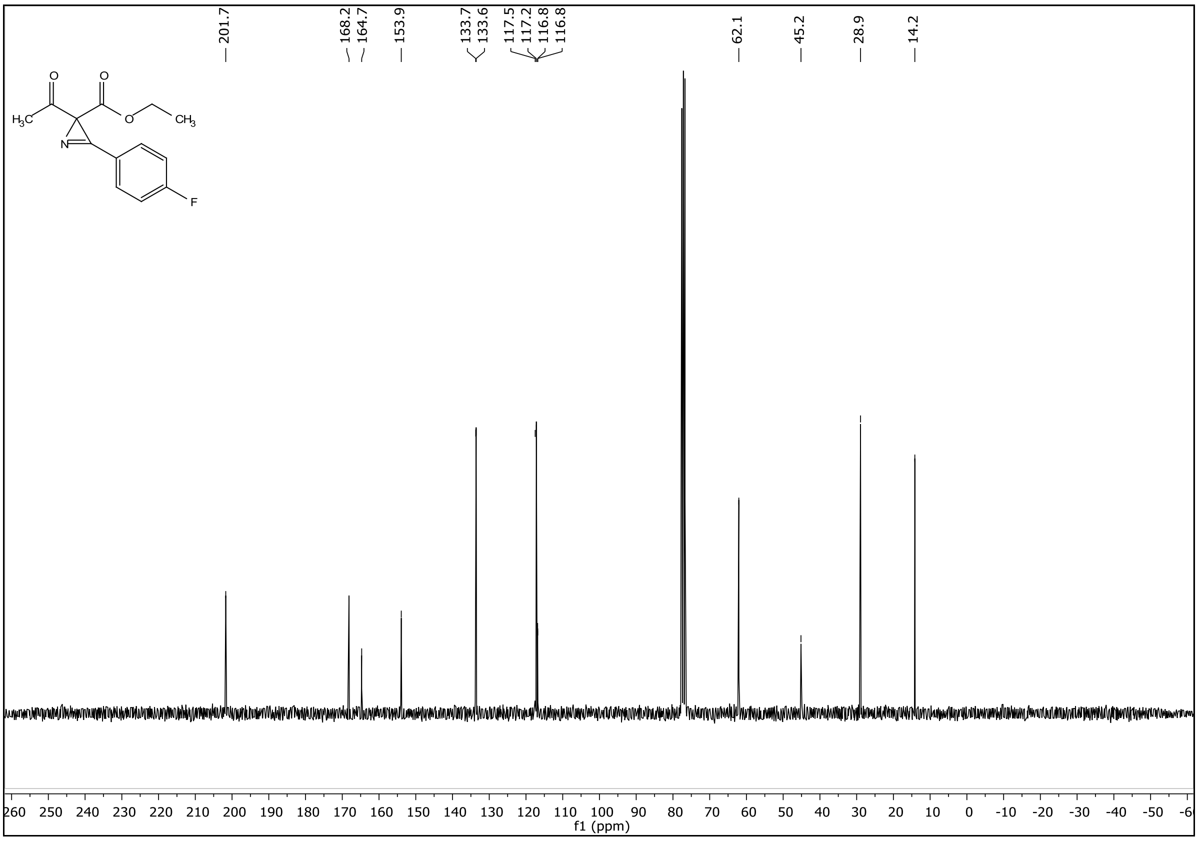
201.7
168.2
164.7
153.9
133.7
133.6
117.5
117.2
116.8
116.8
62.1
45.2
28.9
14.2
O
O
H3C
O
CH3
N
F
250 240 230 220 210 200 190 180 170 160 150 140 130 120 110 100 90 80 70 60 50 40 30 20 10 0 -10 -20 -30 -40 -50
f1 (ppm)

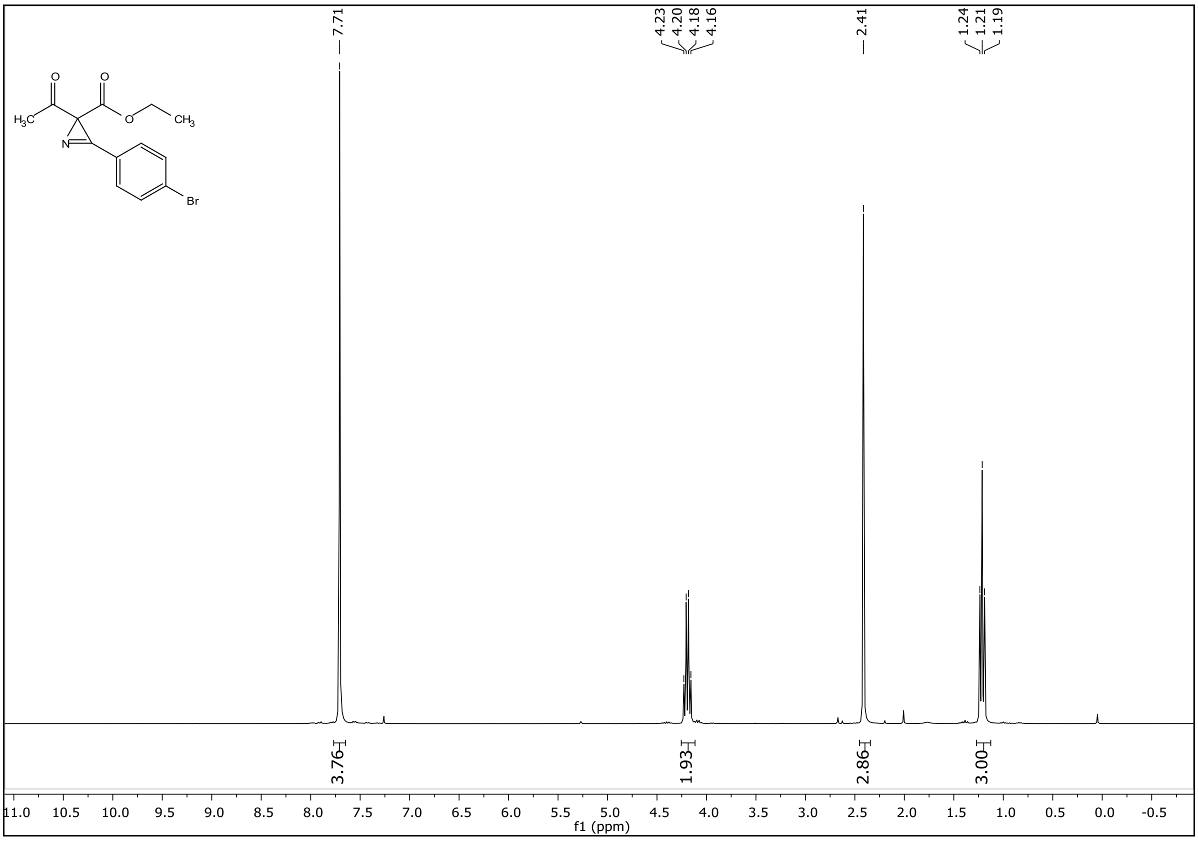
7.71
4.23
4.20
4.18
4.16
2.41
1.24
1.21
1.19
H3C
O
O
O
CH3
N
Br
3.76
1.93
2.86
3.00
11.0 10.5 10.0 9.5 9.0 8.5 8.0 7.5 7.0 6.5 6.0 5.5 5.0 4.5 4.0 3.5 3.0 2.5 2.0 1.5 1.0 0.5 0.0 -0.5
f1 (ppm)

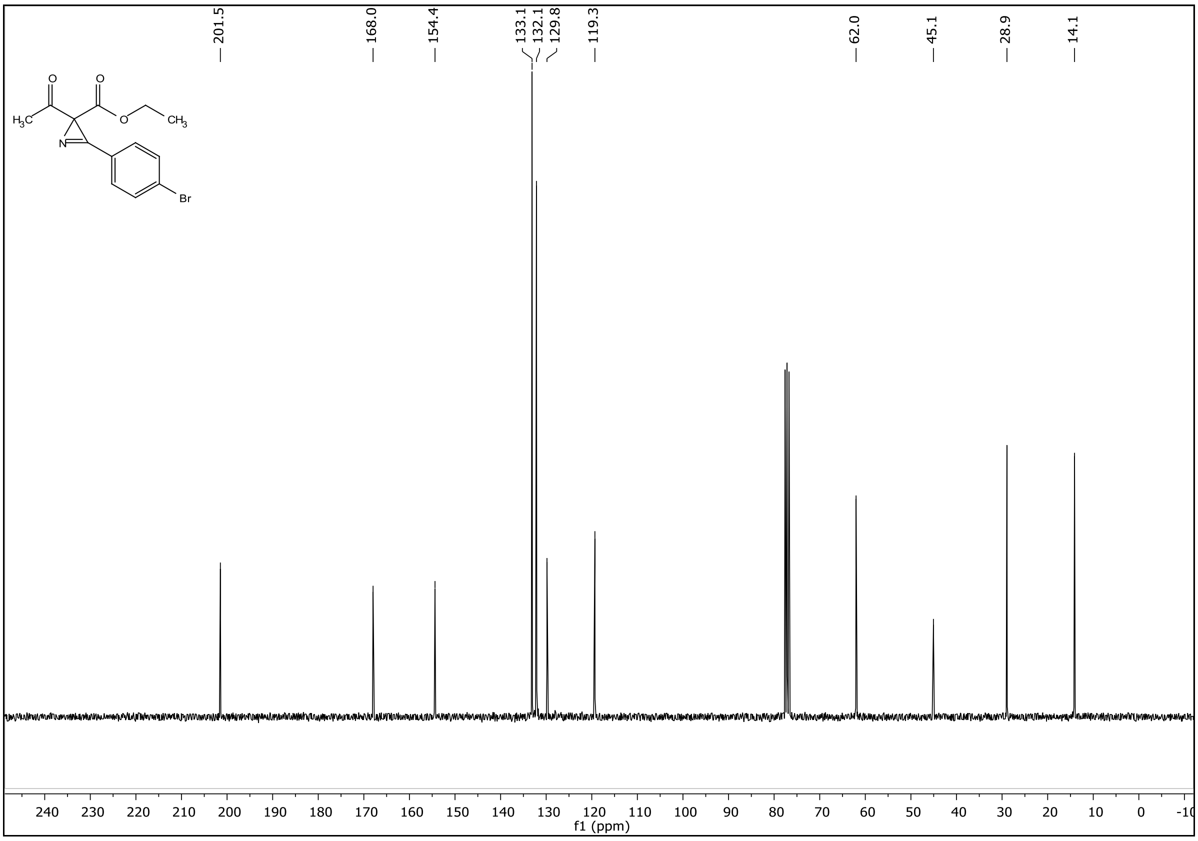
201.5
168.0
154.4
133.1
132.1
129.8
119.3
62.0
45.1
28.9
14.1
H3C
O
O
O
CH3
N
Br
240 230 220 210 200 190 180 170 160 150 140 130 120 110 100 90 80 70 60 50 40 30 20 10 0
f1 (ppm)

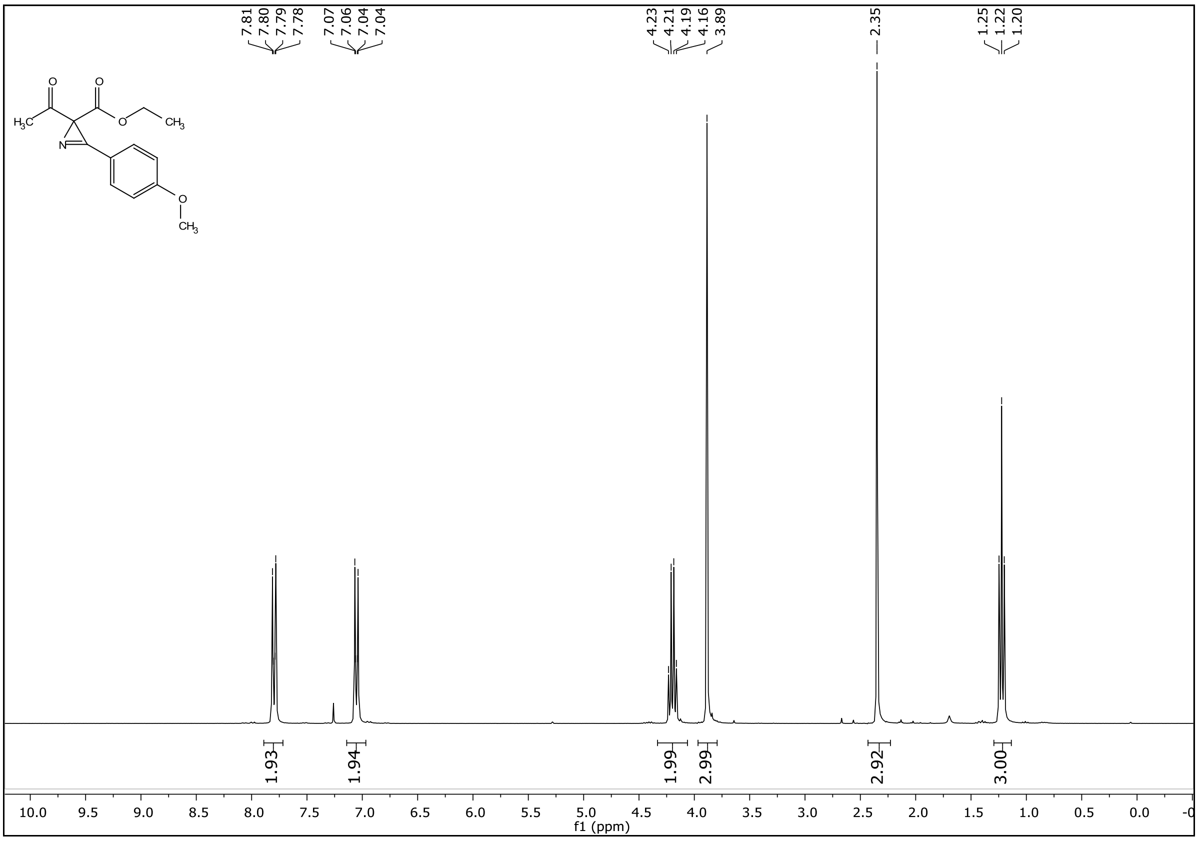
7.81
7.80
7.79
7.78
7.07
7.06
7.04
7.04
4.23
4.21
4.19
4.16
3.89
2.35
1.25
1.22
1.20
1.93
1.94
1.99
2.99
2.92
3.00
f1 (ppm)

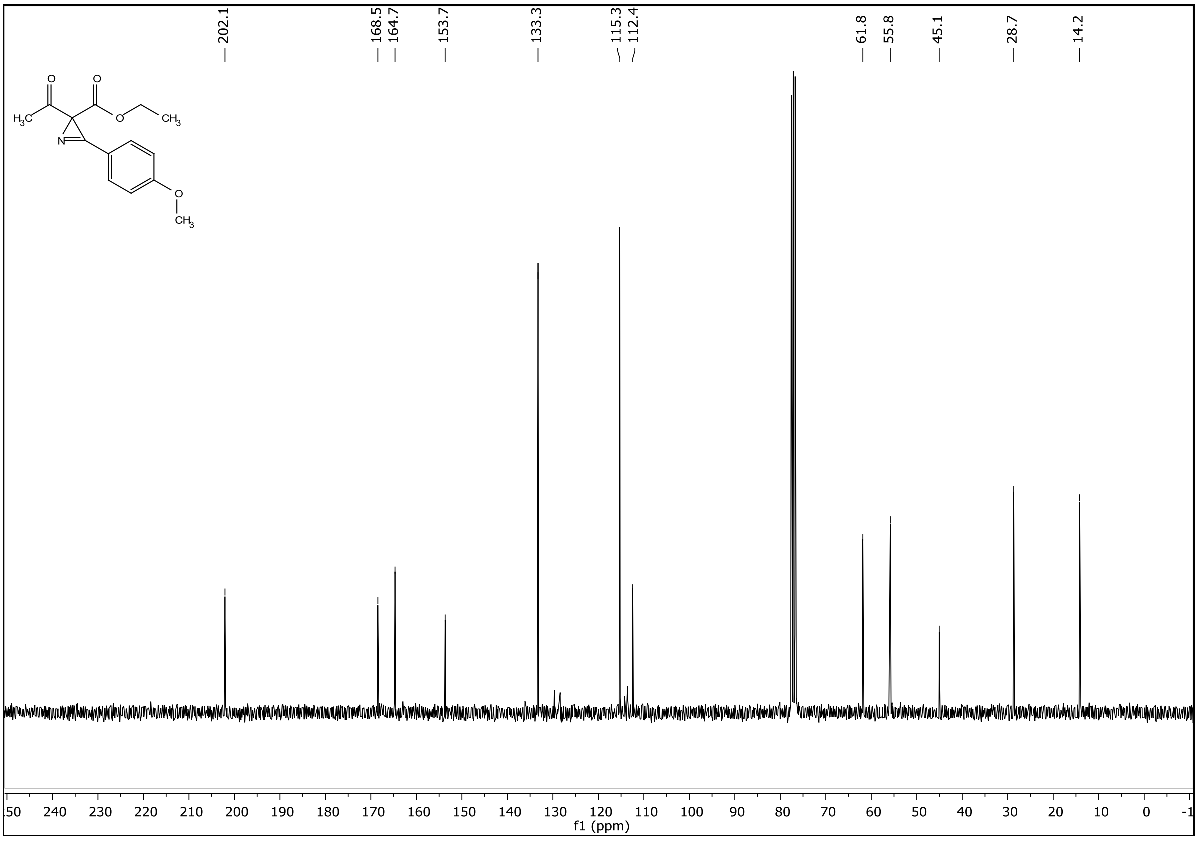
202.1
168.5
164.7
153.7
133.3
115.3
112.4
61.8
55.8
45.1
28.7
14.2
f1 (ppm)

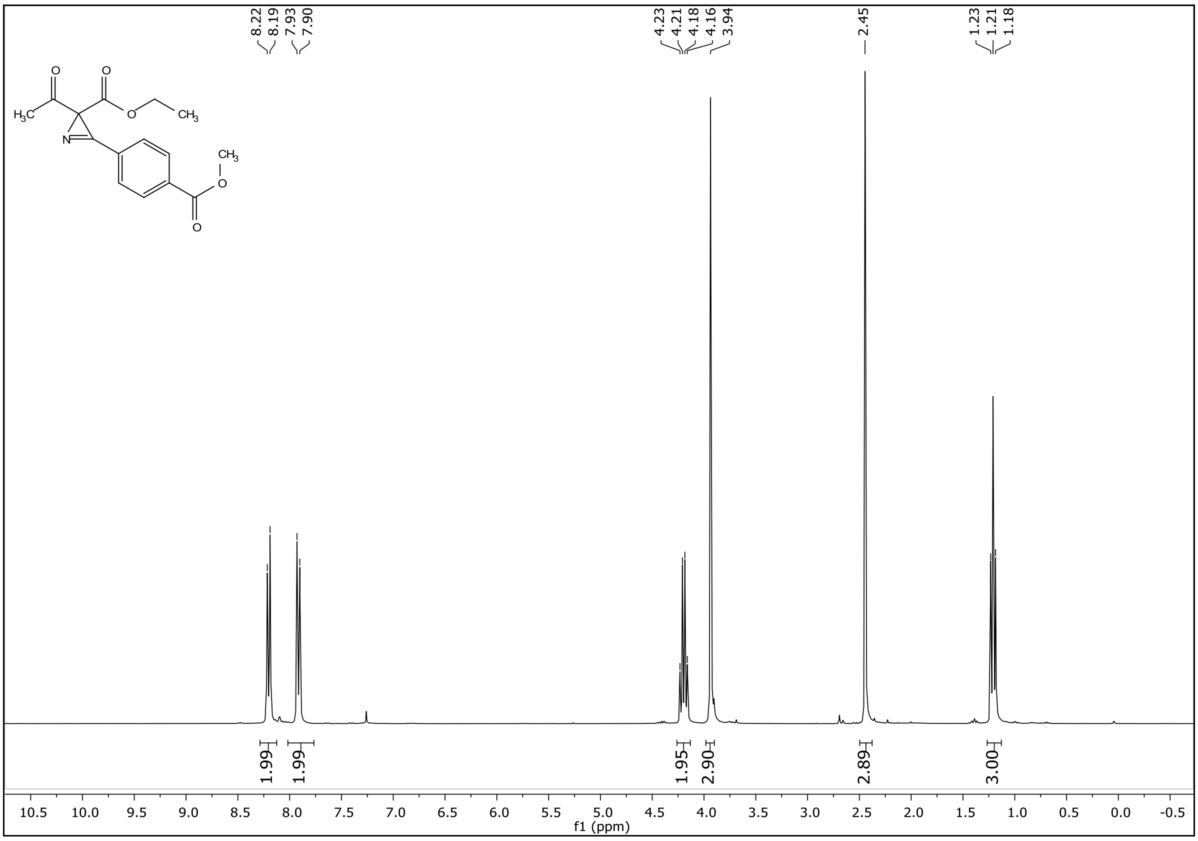
8.22
8.19
7.93
7.90
4.23
4.21
4.18
4.16
3.94
2.45
1.23
1.21
1.18
1.99
1.99
1.95
2.90
2.89
3.00
f1 (ppm)

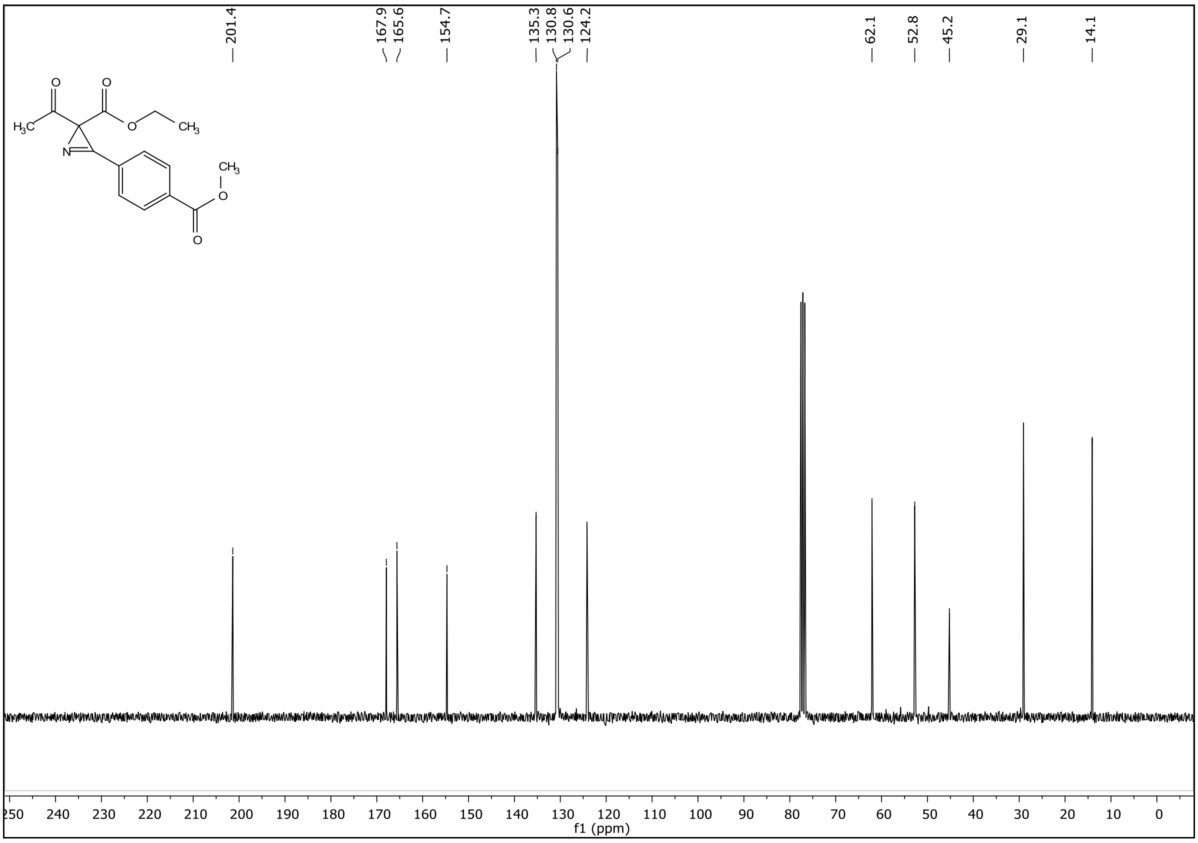
201.4
167.9
165.6
154.7
135.3
130.8
130.6
124.2
62.1
52.8
45.2
29.1
14.1
f1 (ppm)

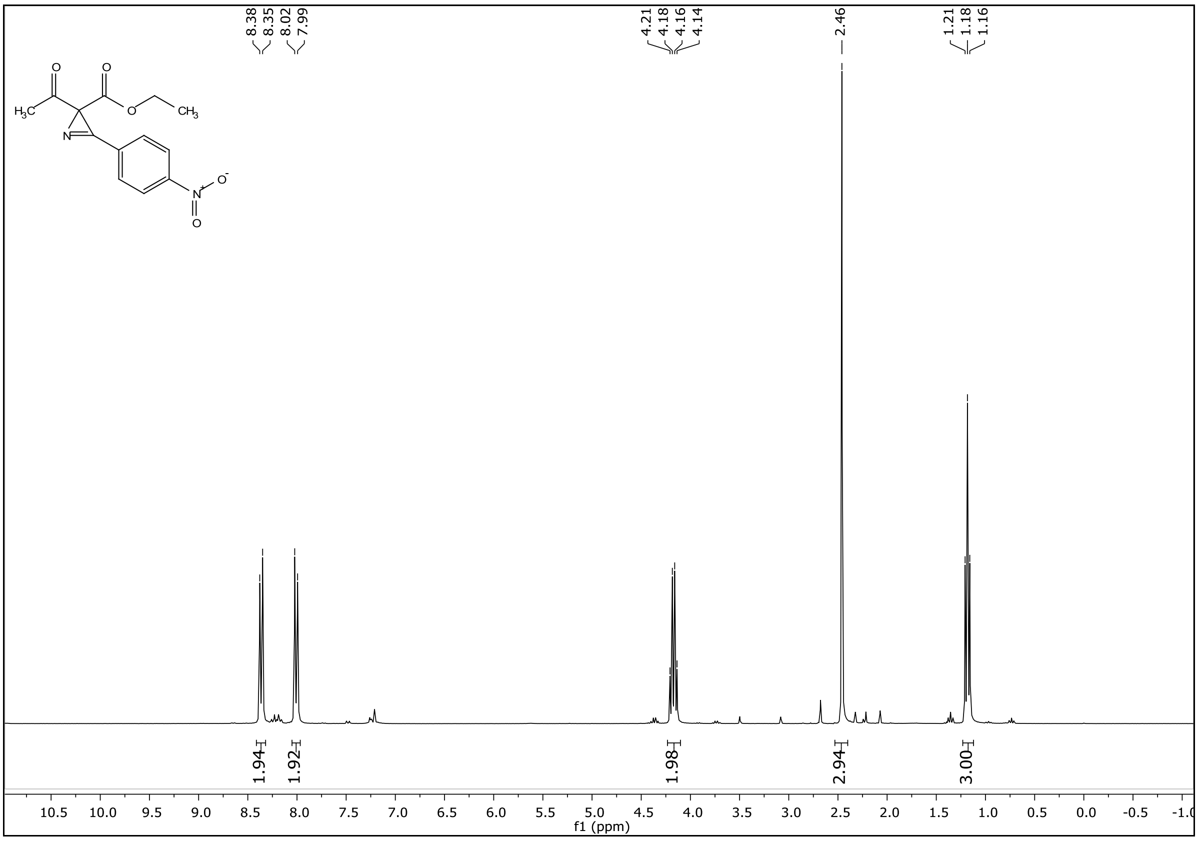
8.38
8.35
8.02
7.99
4.21
4.18
4.16
4.14
2.46
1.21
1.18
1.16
O
O
H3C
O
CH3
N
N+
O-
O
1.94
1.92
1.98
2.94
3.00
10.5 10.0 9.5 9.0 8.5 8.0 7.5 7.0 6.5 6.0 5.5 5.0 4.5 4.0 3.5 3.0 2.5 2.0 1.5 1.0 0.5 0.0 -0.5
f1 (ppm)

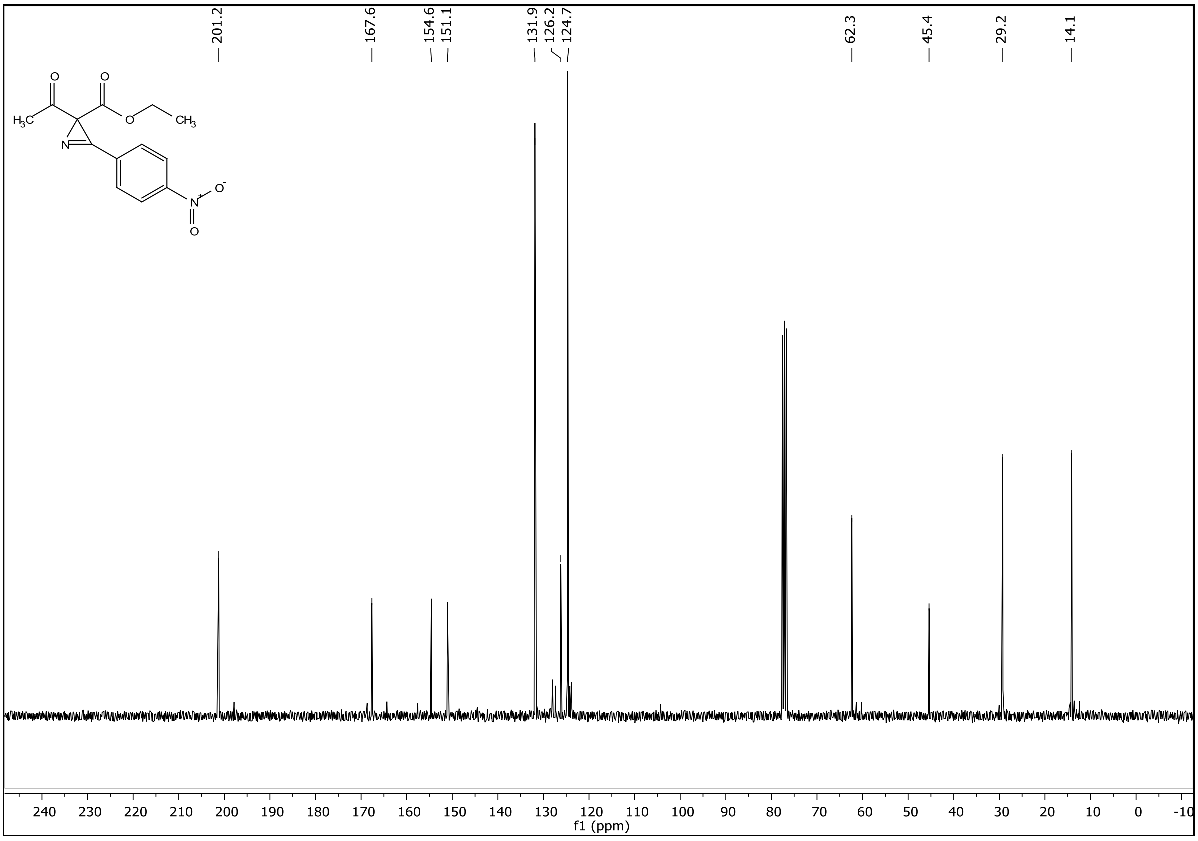
201.2
167.6
154.6
151.1
131.9
126.2
124.7
62.3
45.4
29.2
14.1
O
O
H3C
O
CH3
N
N+
O-
O
240 230 220 210 200 190 180 170 160 150 140 130 120 110 100 90 80 70 60 50 40 30 20 10 0
f1 (ppm)

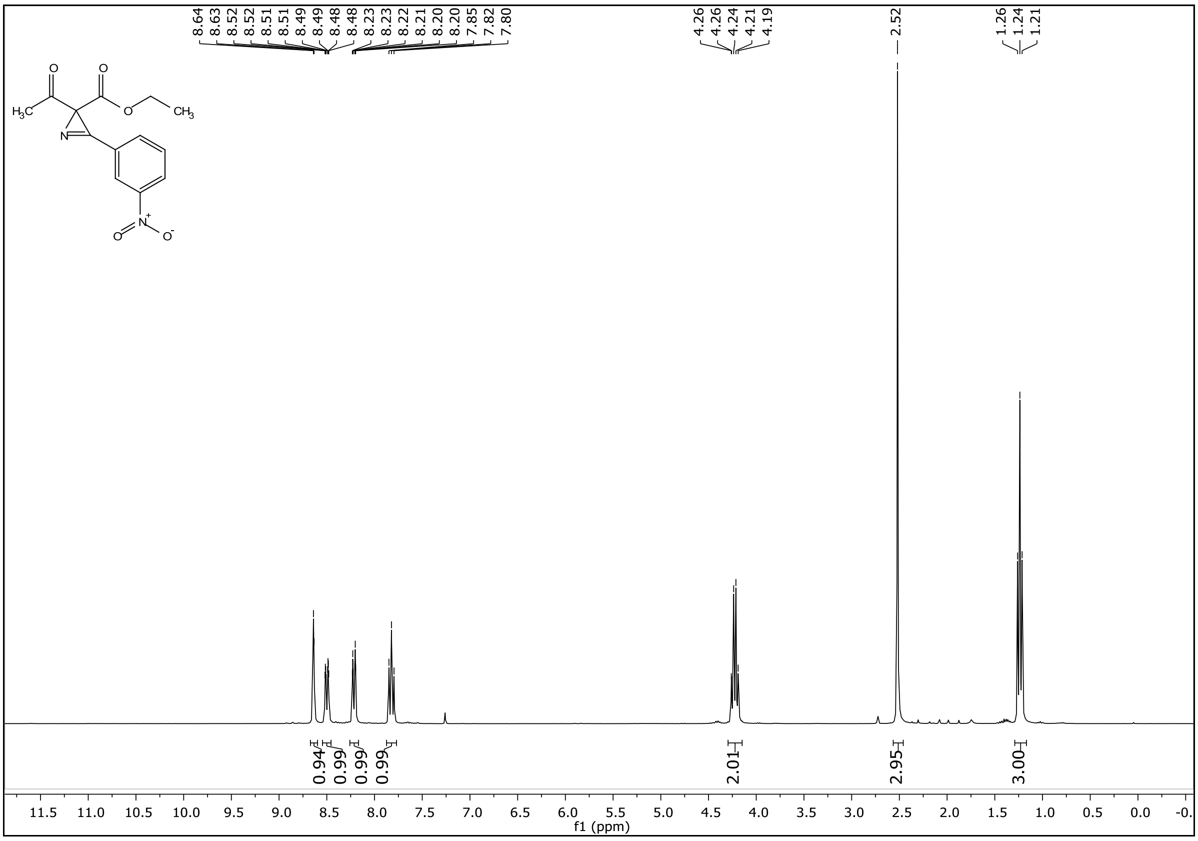
8.64
8.63
8.52
8.52
8.51
8.51
8.49
8.48
8.48
8.23
8.23
8.22
8.21
8.20
8.20
7.85
7.82
7.80
4.26
4.26
4.24
4.21
4.19
2.52
1.26
1.24
1.21
0.94
0.99
0.99
0.99
2.01
2.95
3.00
f1 (ppm)

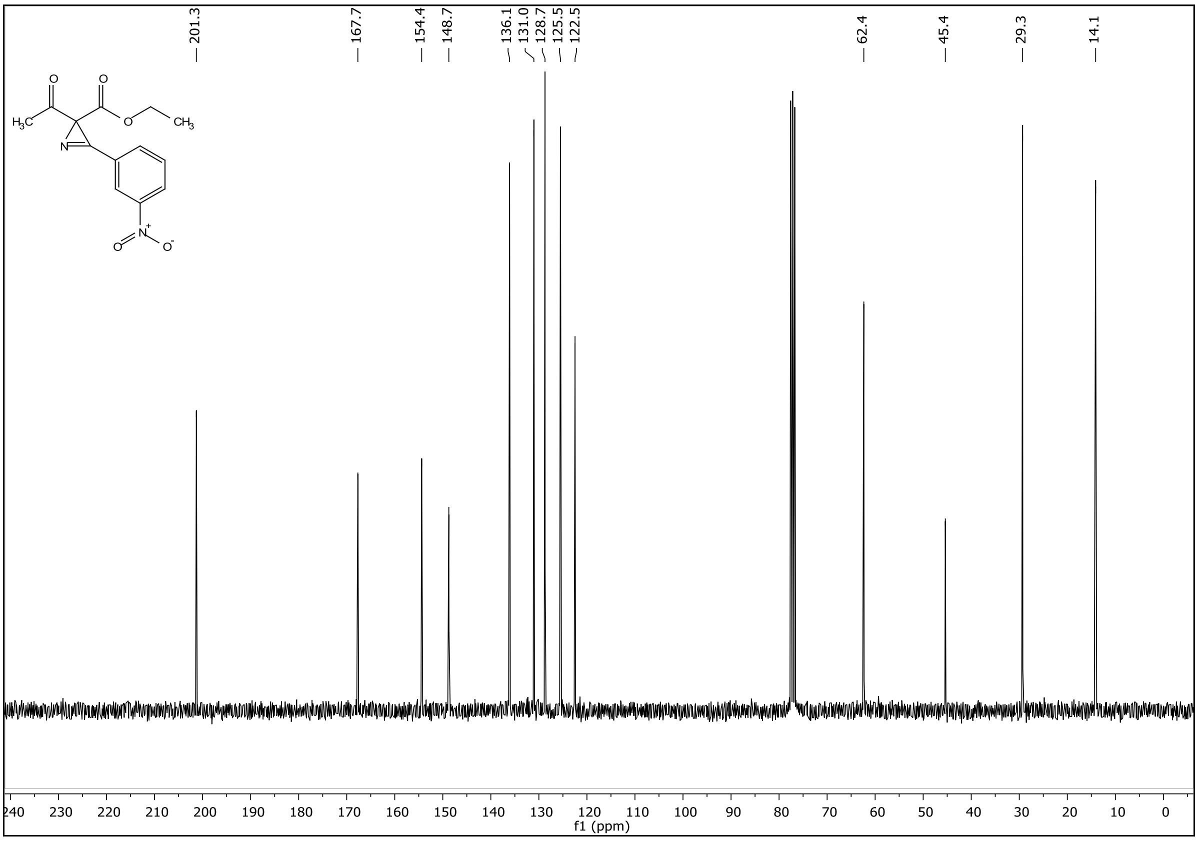
201.3
167.7
154.4
148.7
136.1
131.0
128.7
125.5
122.5
62.4
45.4
29.3
14.1
f1 (ppm)

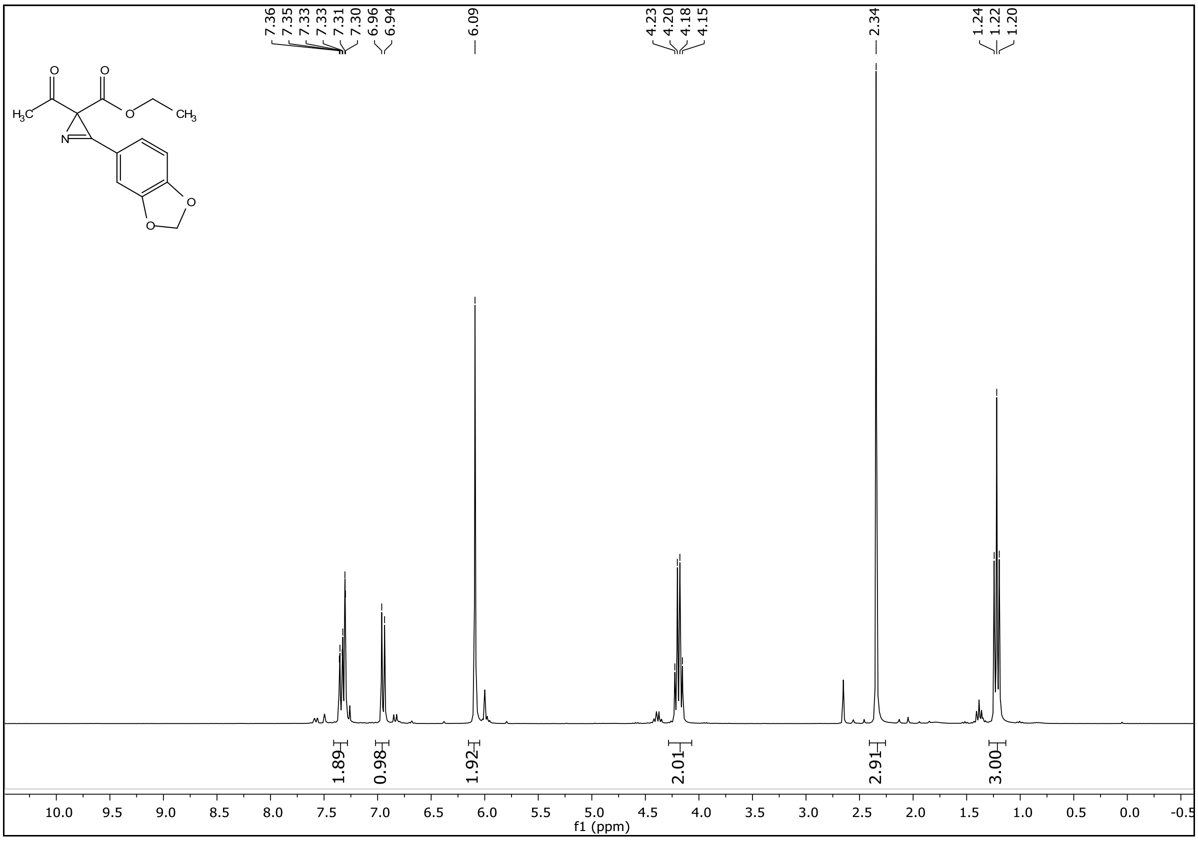
7.36
7.35
7.33
7.33
7.31
7.30
6.96
6.94
6.09
4.23
4.20
4.18
4.15
2.34
1.24
1.22
1.20
1.89
0.98
1.92
2.01
2.91
3.00
f1 (ppm)

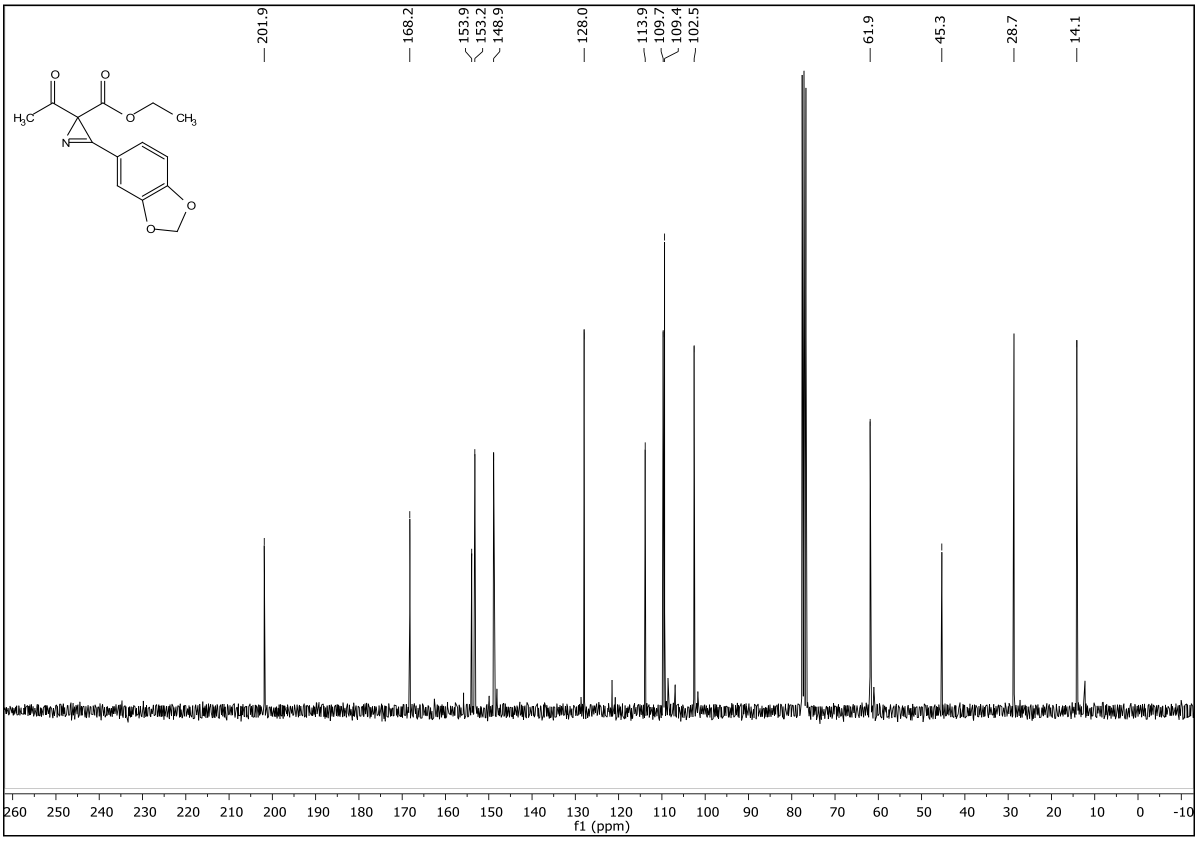
201.9
168.2
153.9
153.2
148.9
128.0
113.9
109.7
109.4
102.5
61.9
45.3
28.7
14.1
f1 (ppm)

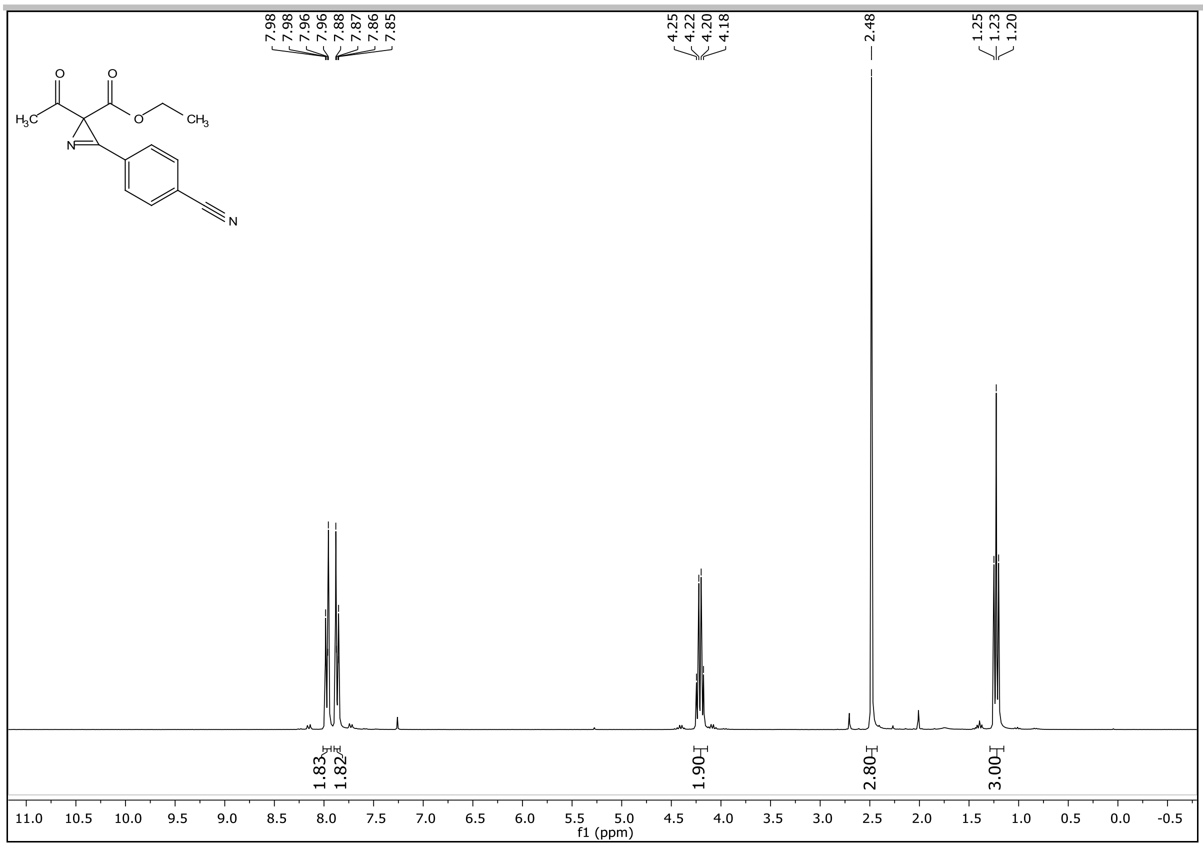
7.98
7.98
7.96
7.96
7.88
7.87
7.86
7.85
4.25
4.22
4.20
4.18
2.48
1.25
1.23
1.20
1.83
1.82
1.90
2.80
3.00
11.0 10.5 10.0 9.5 9.0 8.5 8.0 7.5 7.0 6.5 6.0 5.5 5.0 4.5 4.0 3.5 3.0 2.5 2.0 1.5 1.0 0.5 0.0 -0.5
f1 (ppm)

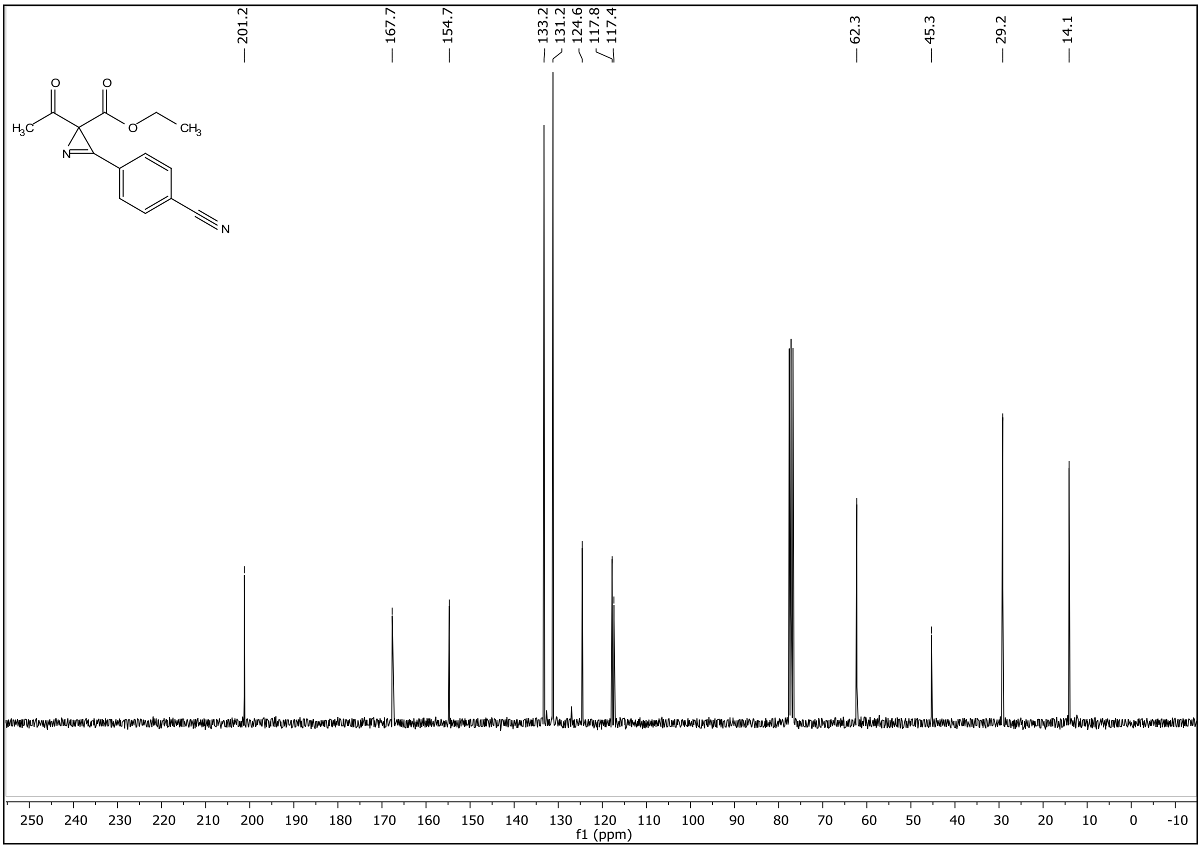
201.2
167.7
154.7
133.2
131.2
124.6
117.8
117.4
62.3
45.3
29.2
14.1
250 240 230 220 210 200 190 180 170 160 150 140 130 120 110 100 90 80 70 60 50 40 30 20 10 0 -10
f1 (ppm)

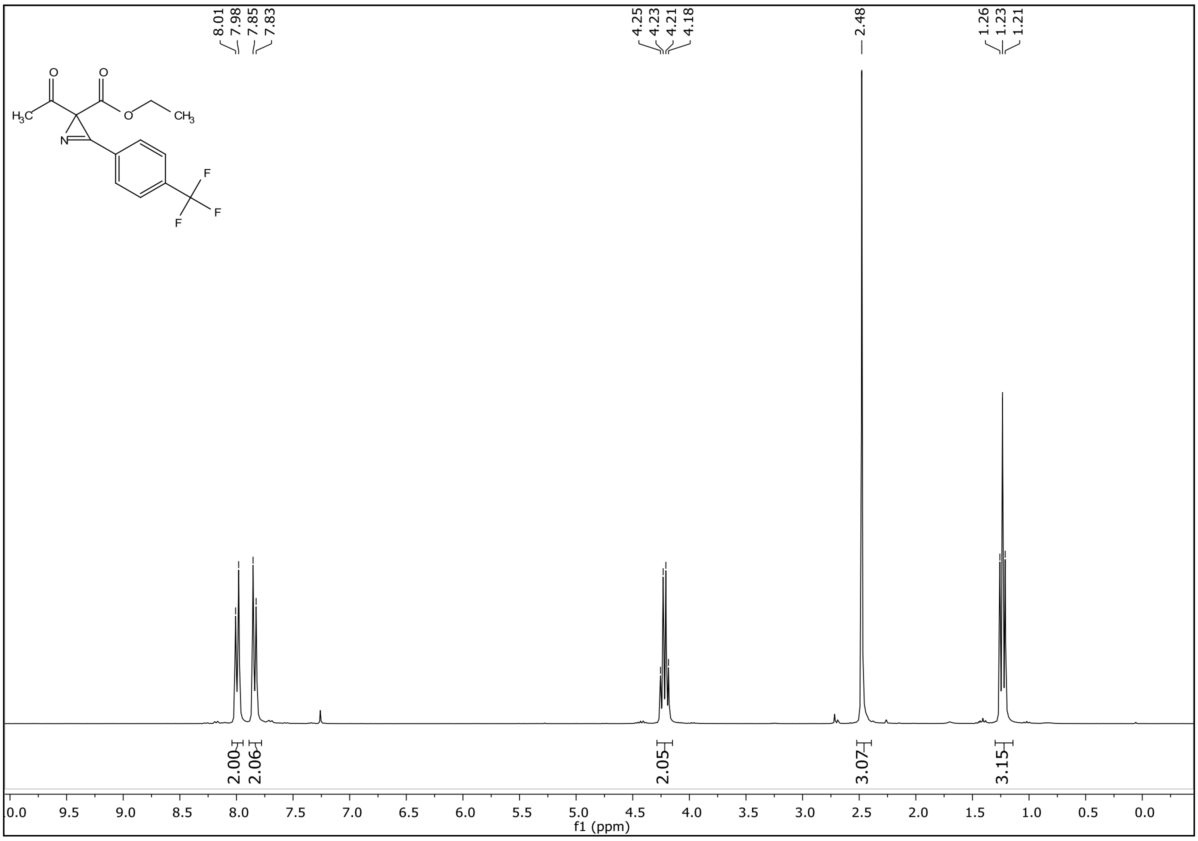
8.01
7.98
7.85
7.83
4.25
4.23
4.21
4.18
2.48
1.26
1.23
1.21
2.00
2.06
2.05
3.07
3.15
f1 (ppm)

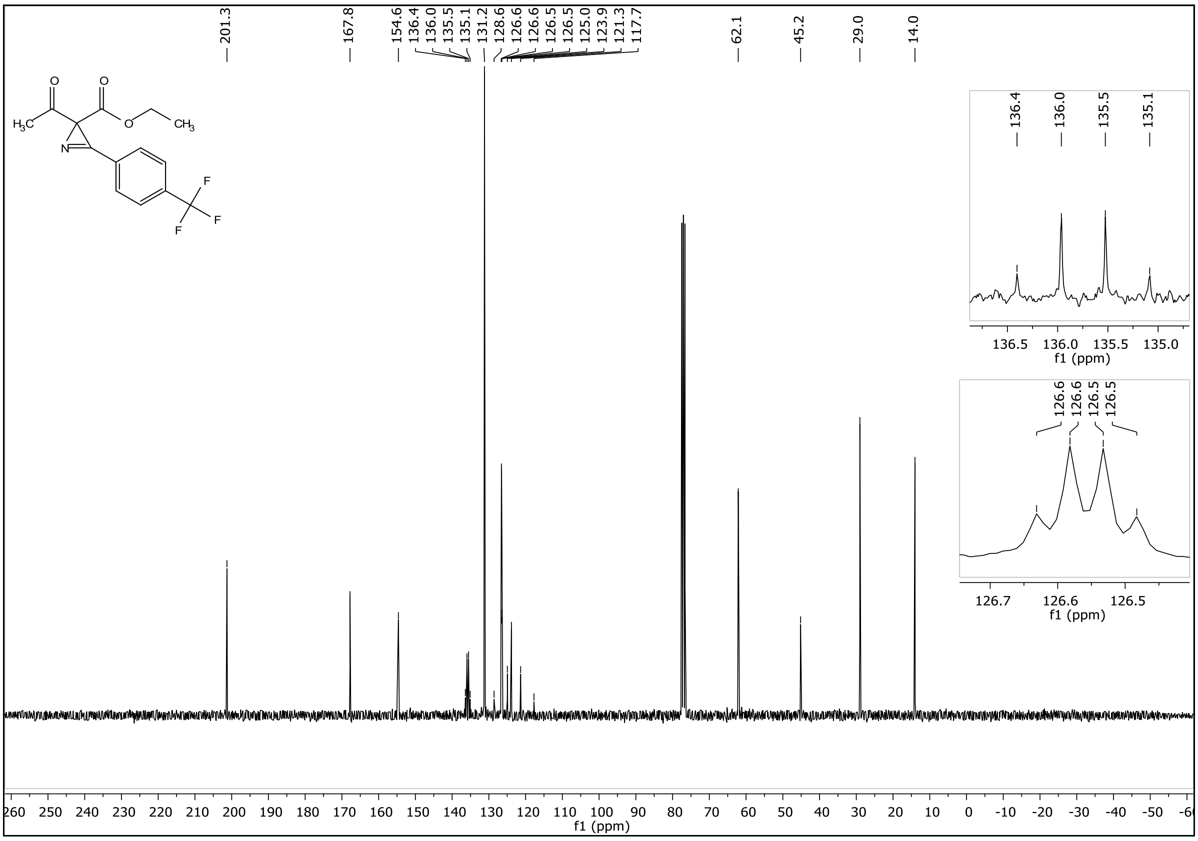
201.3
167.8
154.6
136.4
136.0
135.5
135.1
131.2
128.6
126.6
126.6
126.5
126.5
125.0
123.9
121.3
117.7
62.1
45.2
29.0
14.0
f1 (ppm)

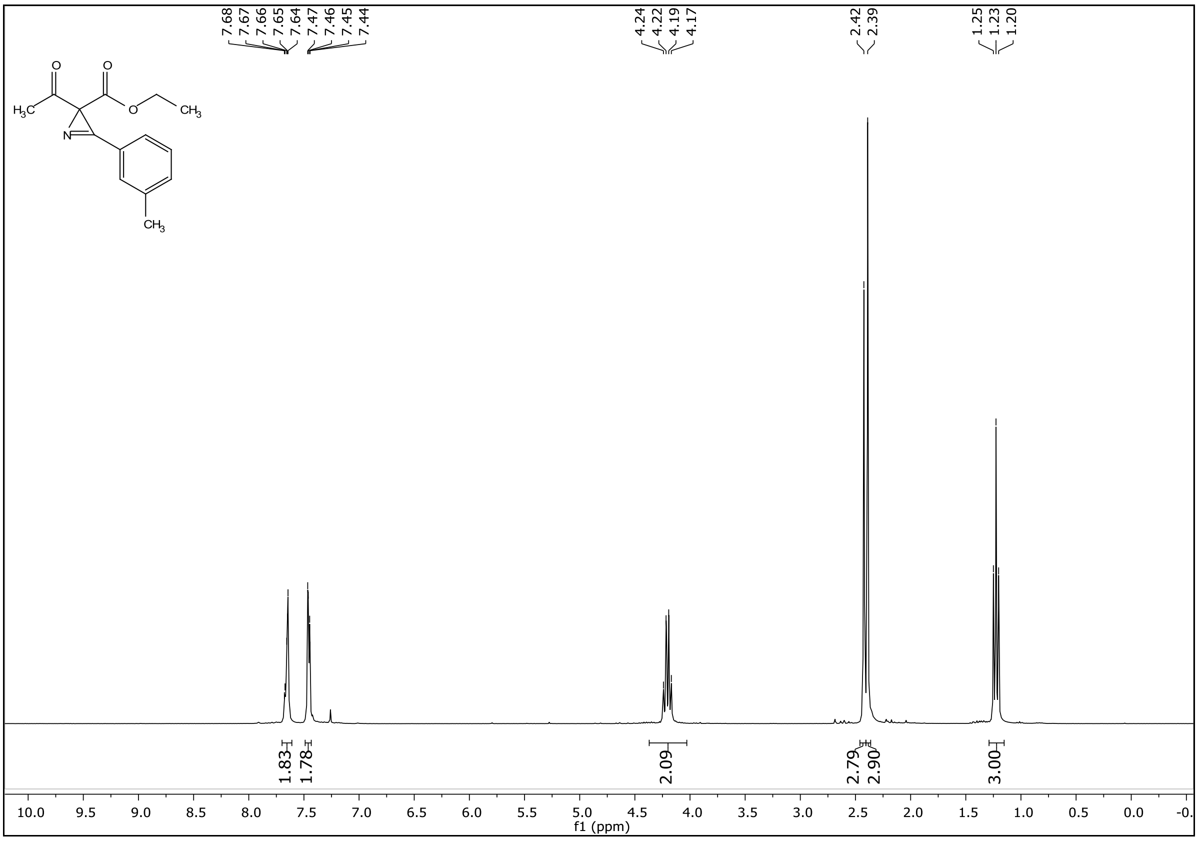
7.68
7.67
7.66
7.65
7.64
7.47
7.46
7.45
7.44
4.24
4.22
4.19
4.17
2.42
2.39
1.25
1.23
1.20
H3C
O
O
O
CH3
N
CH3
1.83
1.78
2.09
2.79
2.90
3.00
10.0
9.5
9.0
8.5
8.0
7.5
7.0
6.5
6.0
5.5
5.0
4.5
4.0
3.5
3.0
2.5
2.0
1.5
1.0
0.5
0.0
f1 (ppm)

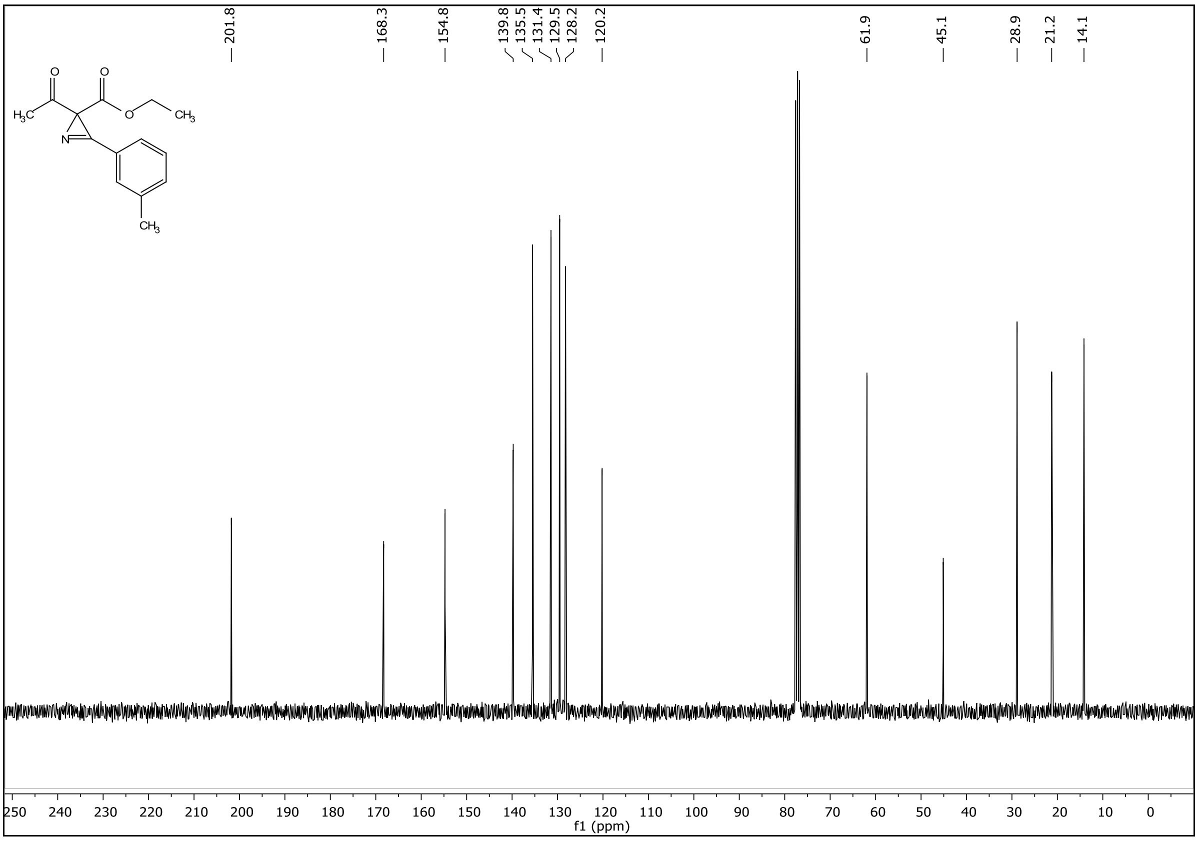
201.8
168.3
154.8
139.8
135.5
131.4
129.5
128.2
120.2
61.9
45.1
28.9
21.2
14.1
H3C
O
O
O
CH3
N
CH3
250
240
230
220
210
200
190
180
170
160
150
140
130
120
110
100
90
80
70
60
50
40
30
20
10
0
f1 (ppm)

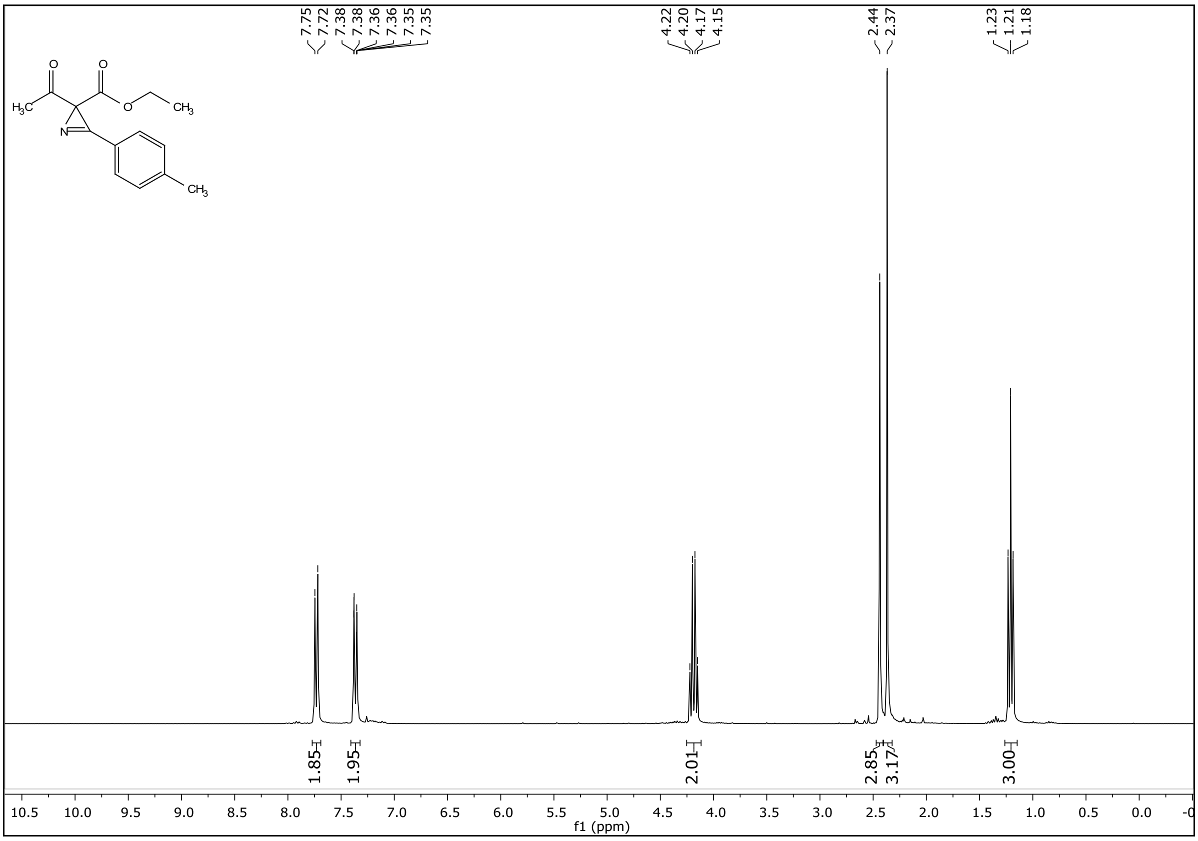
7.75
7.72
7.38
7.38
7.36
7.36
7.35
7.35
4.22
4.20
4.17
4.15
2.44
2.37
1.23
1.21
1.18
1.85
1.95
2.01
2.85
3.17
3.00
f1 (ppm)

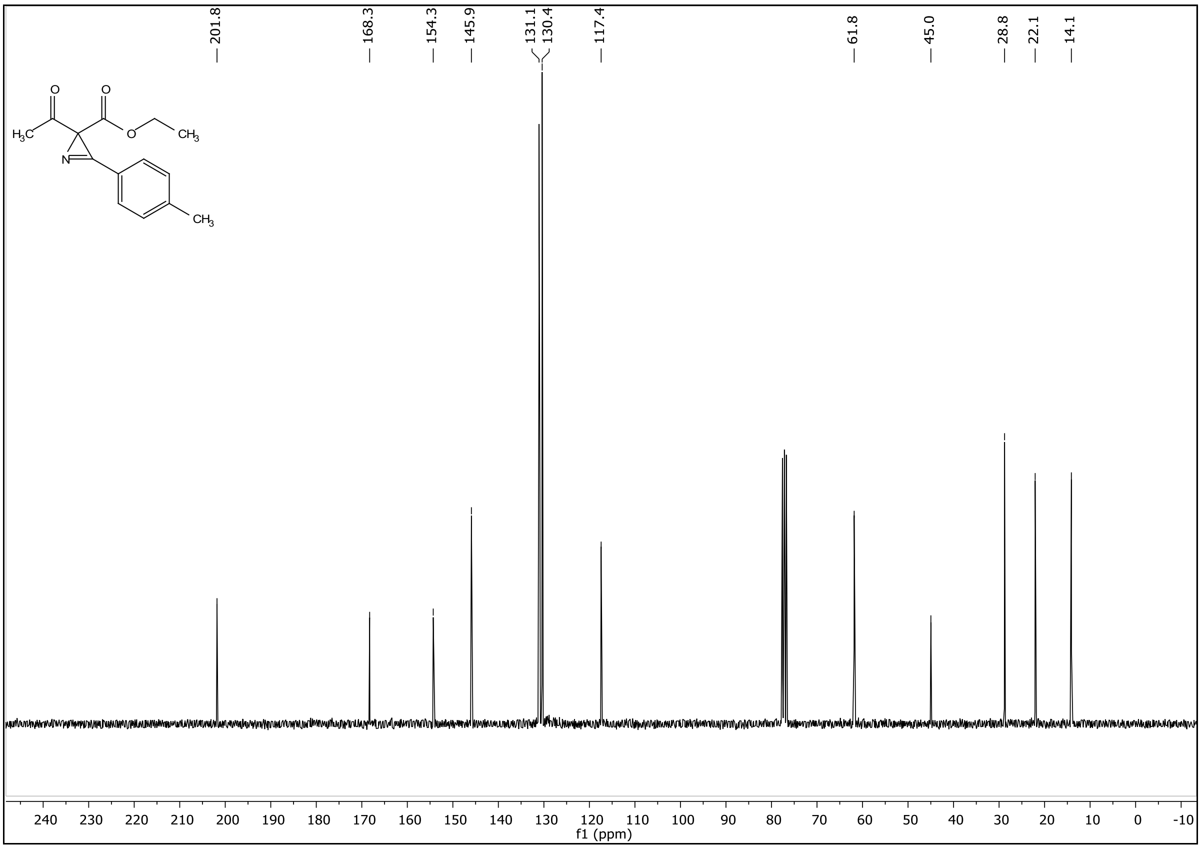
201.8
168.3
154.3
145.9
131.1
130.4
117.4
61.8
45.0
28.8
22.1
14.1
f1 (ppm)

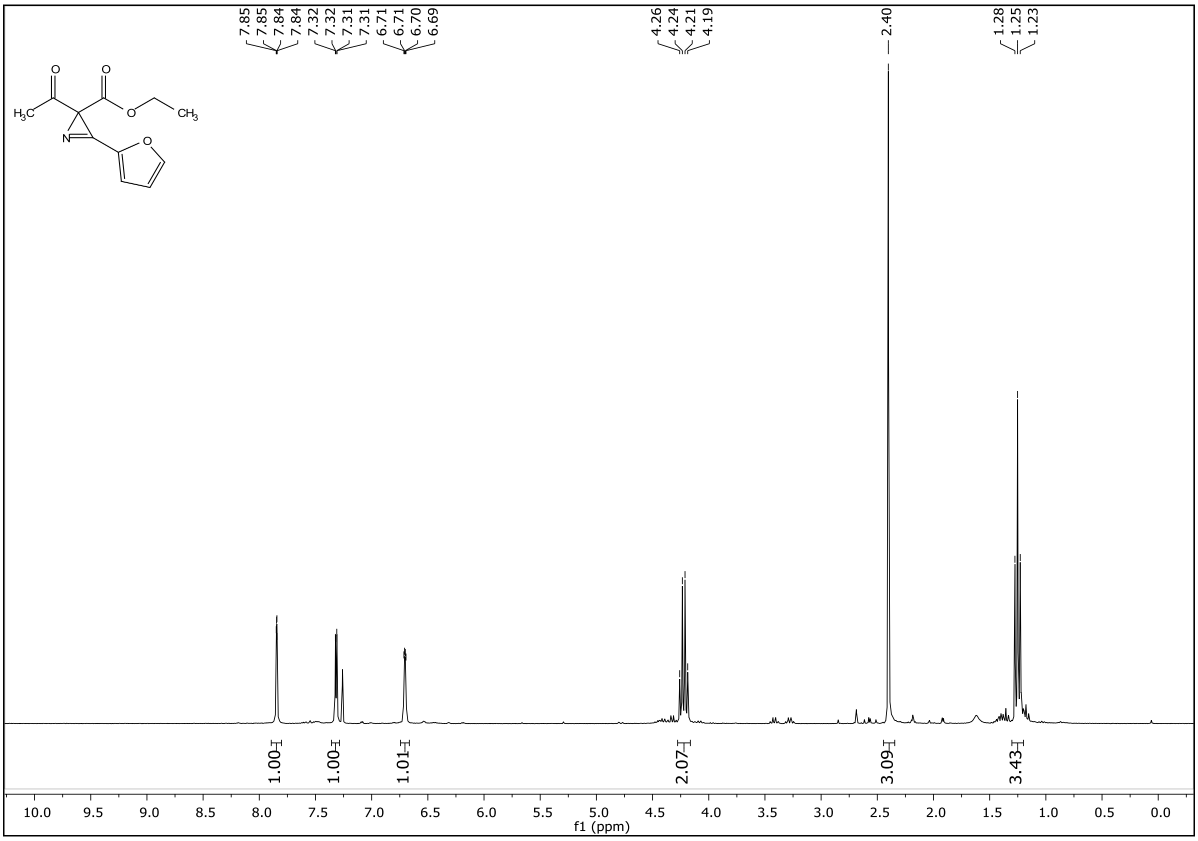
7.85
7.85
7.84
7.84
7.32
7.32
7.31
7.31
6.71
6.71
6.70
6.69
4.26
4.24
4.21
4.19
2.40
1.28
1.25
1.23
O
O
H3C
O
CH3
N
O
1.00
1.00
1.01
2.07
3.09
3.43
10.0 9.5 9.0 8.5 8.0 7.5 7.0 6.5 6.0 5.5 5.0 4.5 4.0 3.5 3.0 2.5 2.0 1.5 1.0 0.5 0.0
f1 (ppm)

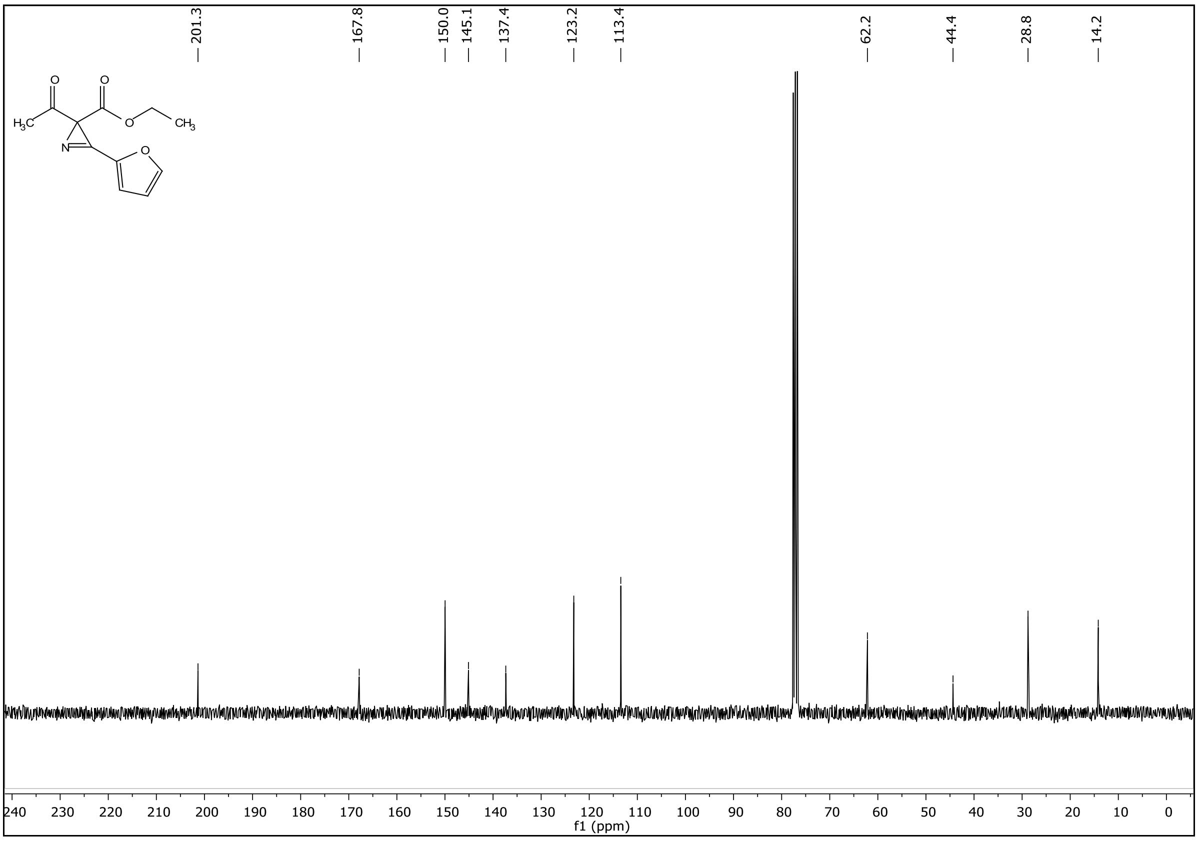
201.3
167.8
150.0
145.1
137.4
123.2
113.4
62.2
44.4
28.8
14.2
O
O
H3C
O
CH3
N
O
240 230 220 210 200 190 180 170 160 150 140 130 120 110 100 90 80 70 60 50 40 30 20 10 0
f1 (ppm)

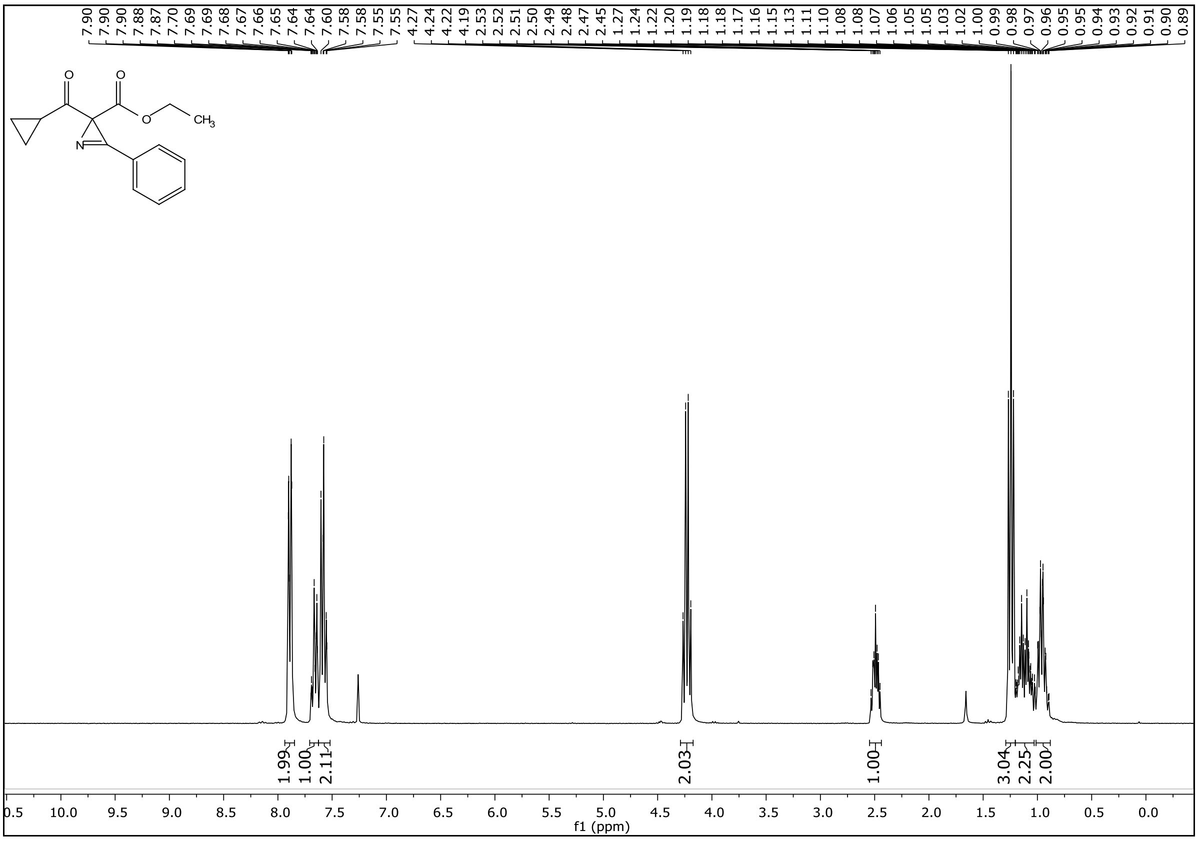
7.90
7.90
7.88
7.87
7.70
7.69
7.69
7.68
7.67
7.66
7.65
7.64
7.64
7.60
7.58
7.58
7.55
7.55
4.27
4.24
4.22
4.19
2.53
2.52
2.51
2.50
2.49
2.48
2.47
2.45
1.27
1.24
1.22
1.20
1.19
1.18
1.18
1.17
1.16
1.15
1.13
1.11
1.10
1.08
1.08
1.07
1.06
1.05
1.05
1.03
1.02
1.00
0.99
0.98
0.97
0.96
0.95
0.95
0.94
0.93
0.92
0.91
0.90
0.89
O
O
O
CH3
N
1.99
1.00
2.11
2.03
1.00
3.04
2.25
2.00
f1 (ppm)

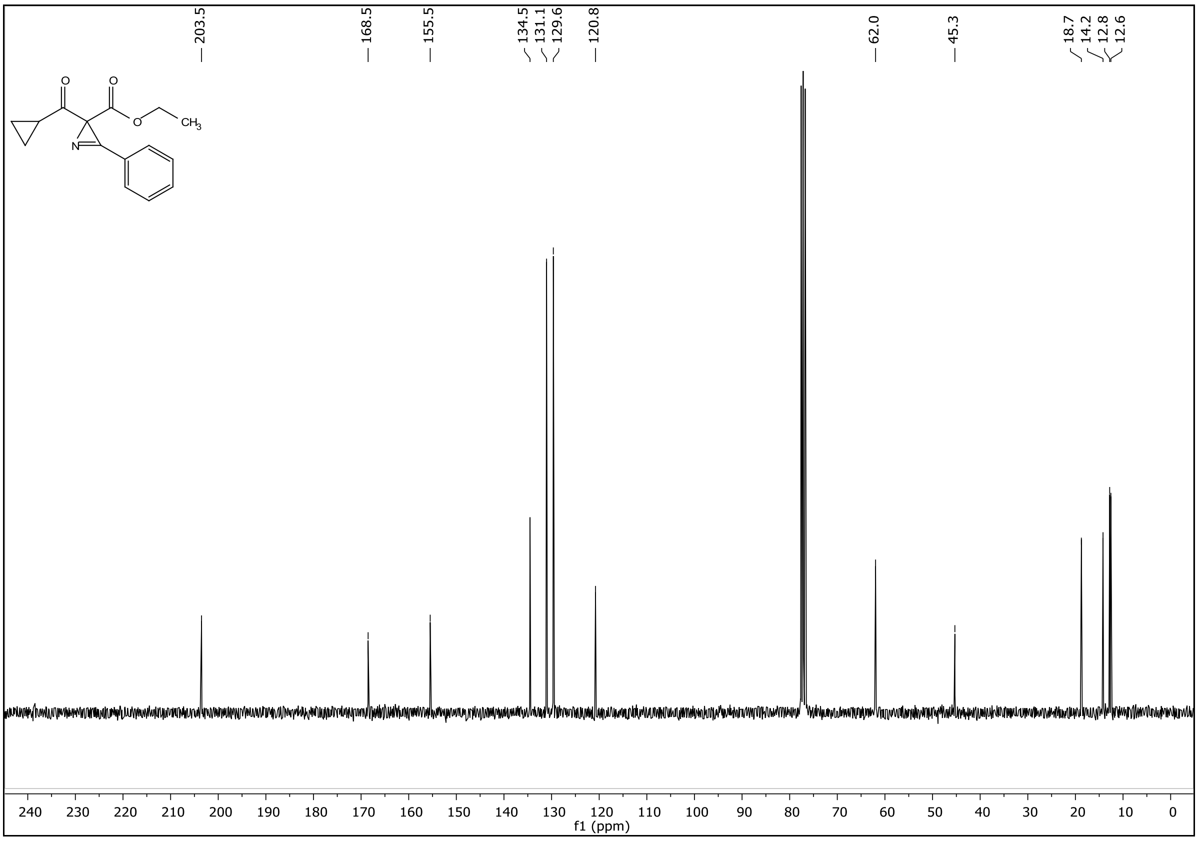
203.5
168.5
155.5
134.5
131.1
129.6
120.8
62.0
45.3
18.7
14.2
12.8
12.6
O
O
O
CH3
N
f1 (ppm)

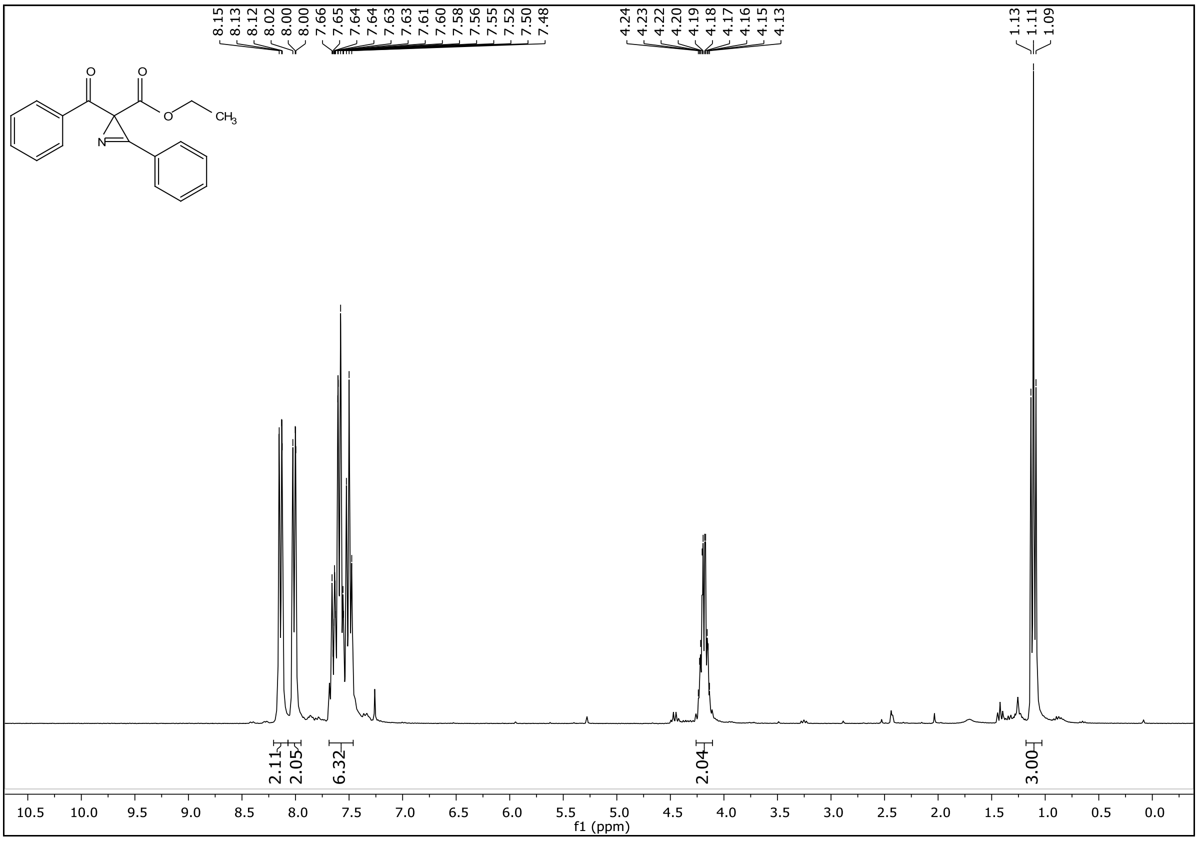
8.15
8.13
8.12
8.02
8.00
8.00
7.66
7.65
7.64
7.64
7.63
7.63
7.61
7.60
7.58
7.56
7.55
7.52
7.50
7.48
4.24
4.23
4.22
4.20
4.19
4.18
4.17
4.16
4.15
4.13
1.13
1.11
1.09
O
O
O
CH3
N
2.11
2.05
6.32
2.04
3.00
10.5 10.0 9.5 9.0 8.5 8.0 7.5 7.0 6.5 6.0 5.5 5.0 4.5 4.0 3.5 3.0 2.5 2.0 1.5 1.0 0.5 0.0
f1 (ppm)

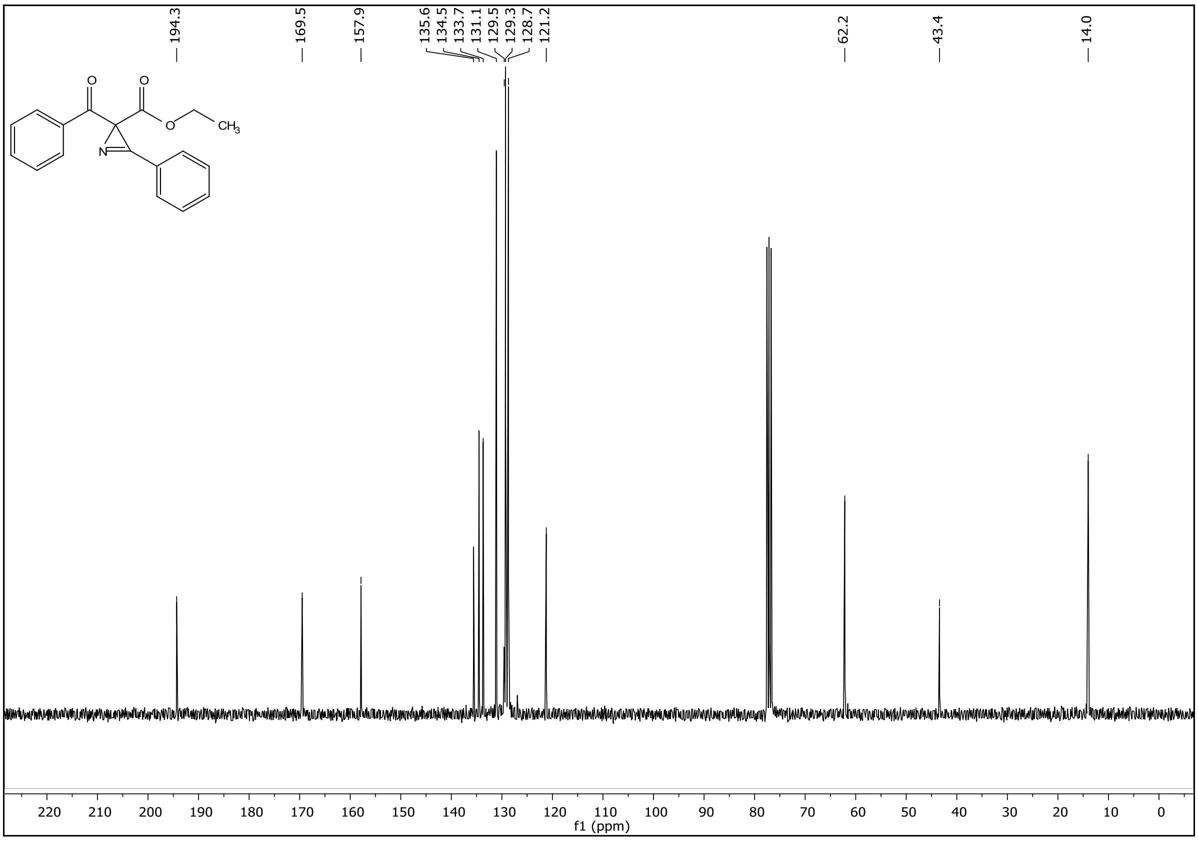
194.3
169.5
157.9
135.6
134.5
133.7
131.1
129.5
129.3
128.7
121.2
62.2
43.4
14.0
O
O
O
CH3
N
220 210 200 190 180 170 160 150 140 130 120 110 100 90 80 70 60 50 40 30 20 10 0
f1 (ppm)

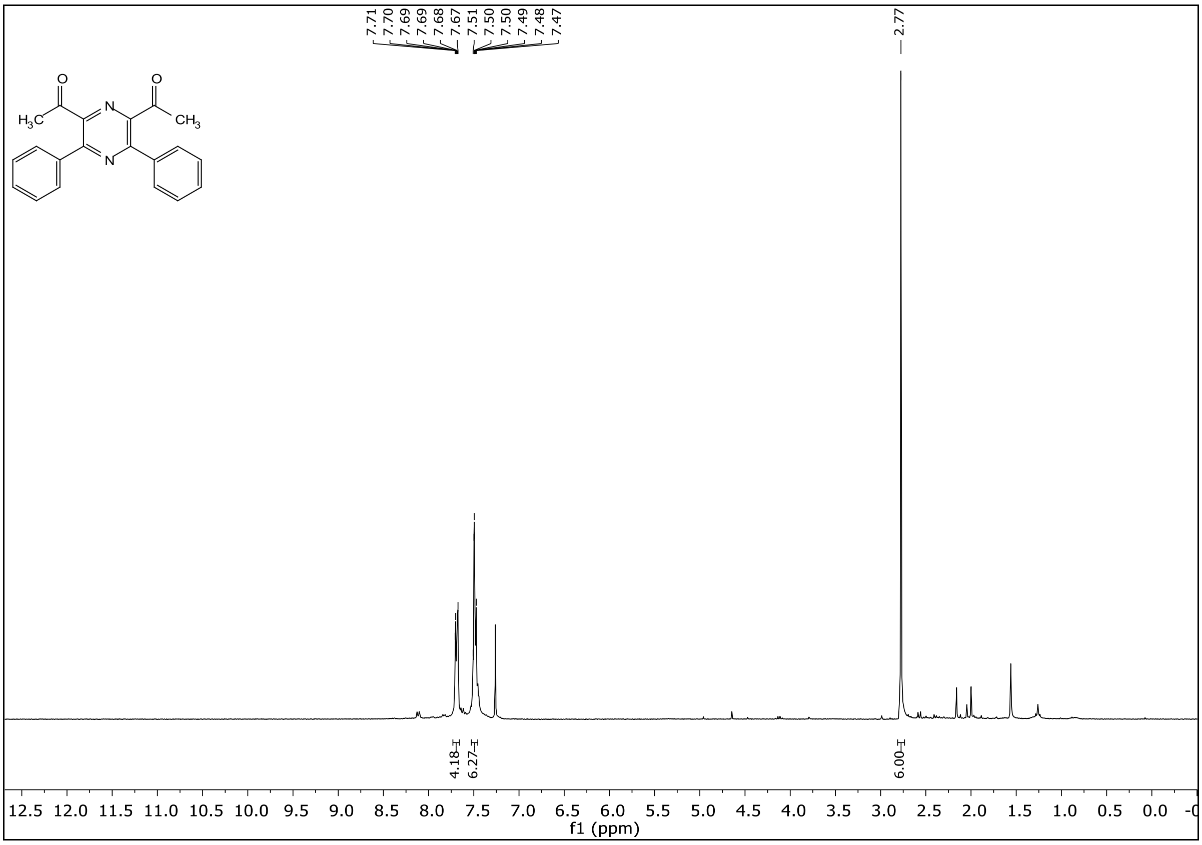
7.71
7.70
7.69
7.69
7.68
7.67
7.51
7.50
7.50
7.49
7.48
7.47
2.77
O
O
N
H3C
CH3
N
4.18
6.27
6.00
12.5 12.0 11.5 11.0 10.5 10.0 9.5 9.0 8.5 8.0 7.5 7.0 6.5 6.0 5.5 5.0 4.5 4.0 3.5 3.0 2.5 2.0 1.5 1.0 0.5 0.0
f1 (ppm)

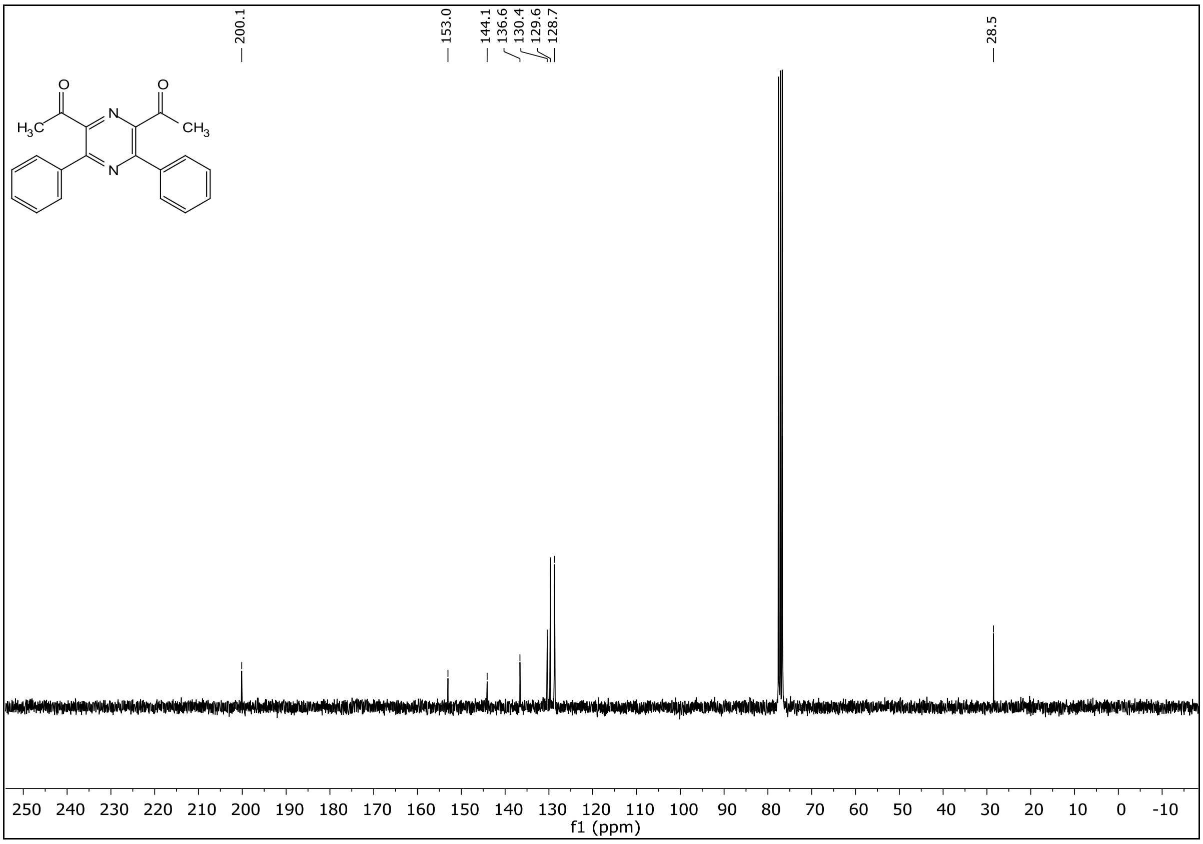
200.1
153.0
144.1
136.6
130.4
129.6
128.7
28.5
O
O
N
H3C
CH3
N
250 240 230 220 210 200 190 180 170 160 150 140 130 120 110 100 90 80 70 60 50 40 30 20 10 0 -10
f1 (ppm)

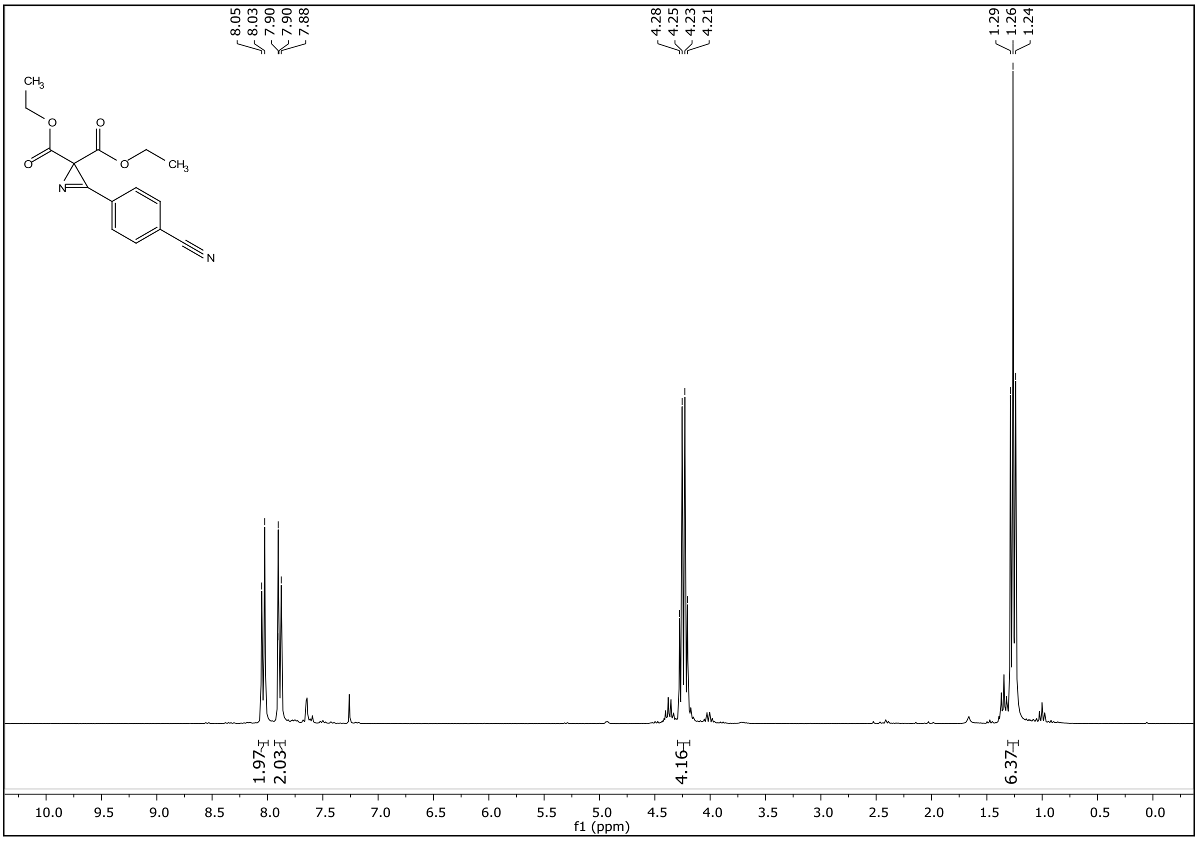
8.05
8.03
7.90
7.90
7.88
4.28
4.25
4.23
4.21
1.29
1.26
1.24
CH3
O
O
O
O
CH3
N
N
1.97
2.03
4.16
6.37
10.0 9.5 9.0 8.5 8.0 7.5 7.0 6.5 6.0 5.5 5.0 4.5 4.0 3.5 3.0 2.5 2.0 1.5 1.0 0.5 0.0
f1 (ppm)

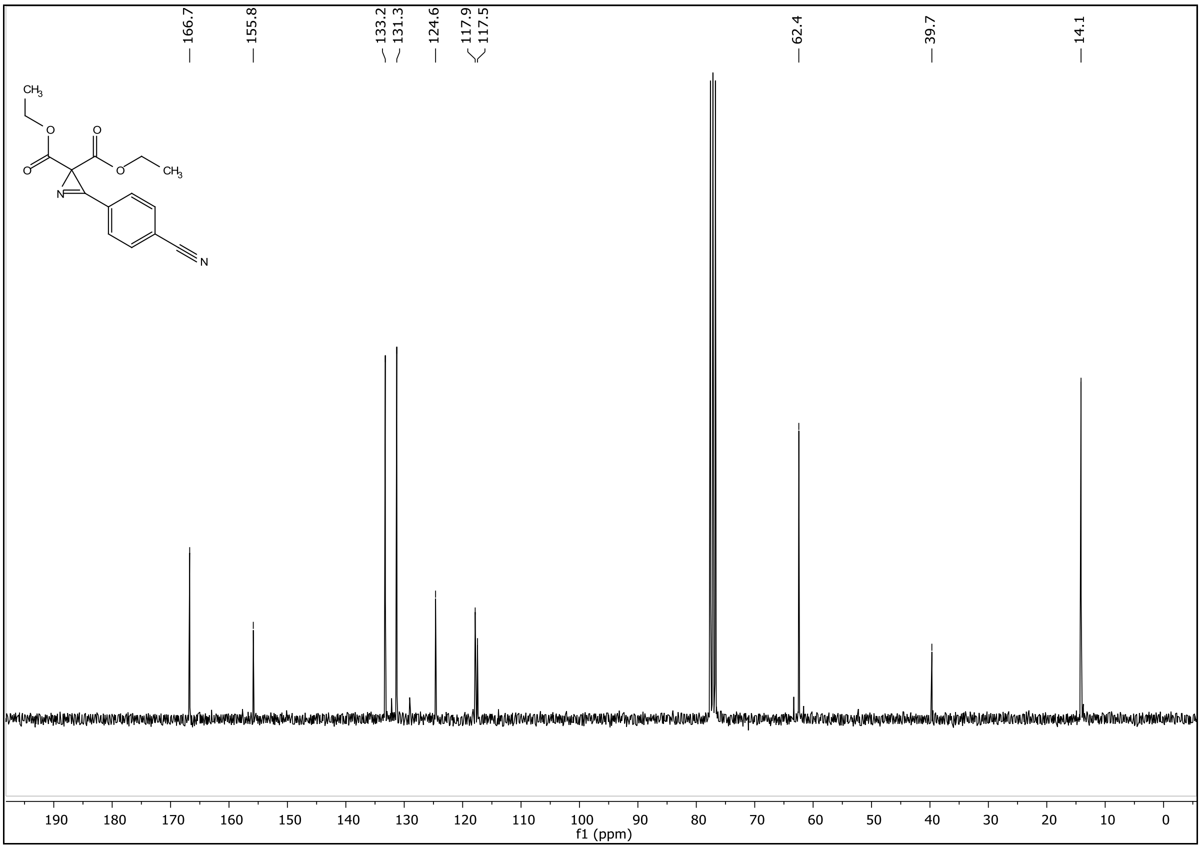
166.7
155.8
133.2
131.3
124.6
117.9
117.5
62.4
39.7
14.1
CH3
O
O
O
O
CH3
N
N
190 180 170 160 150 140 130 120 110 100 90 80 70 60 50 40 30 20 10 0
f1 (ppm)

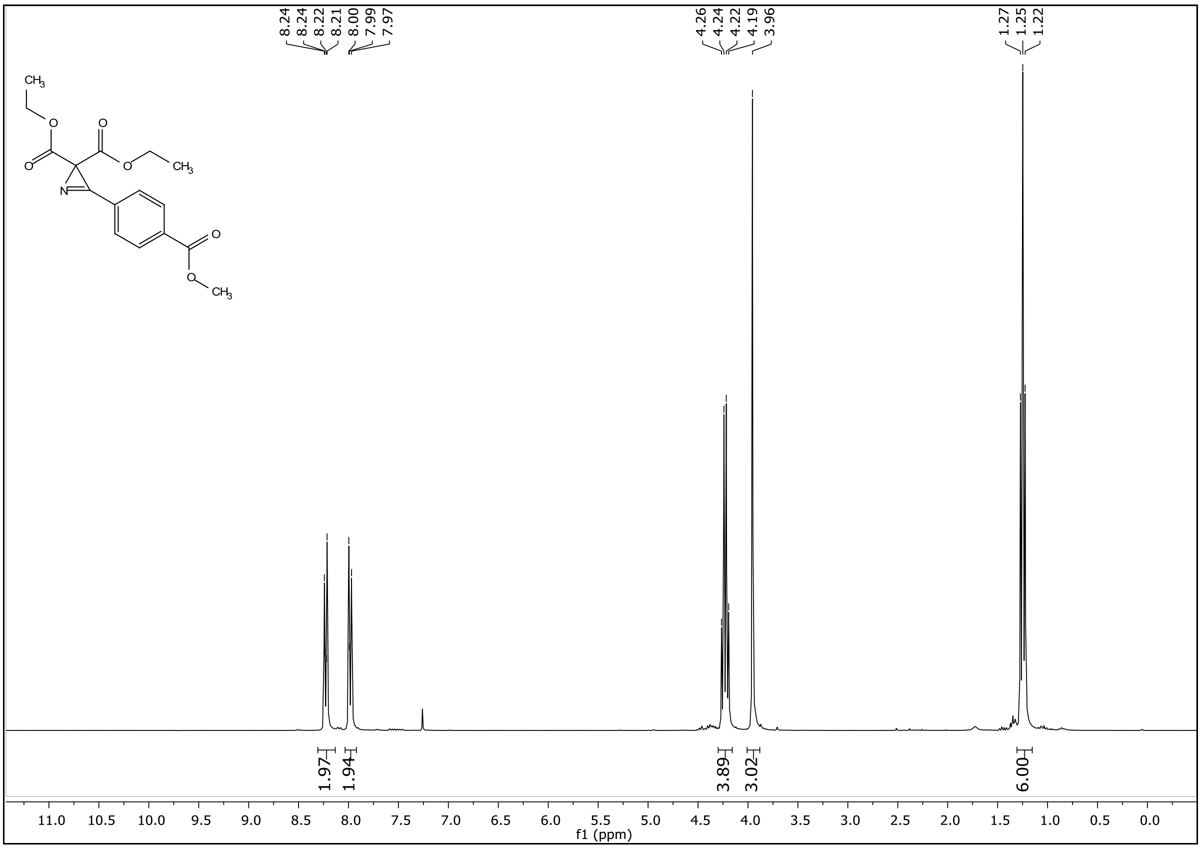
8.24
8.24
8.22
8.21
8.00
7.99
7.97
4.26
4.24
4.22
4.19
3.96
1.27
1.25
1.22
1.97
1.94
3.89
3.02
6.00
11.0 10.5 10.0 9.5 9.0 8.5 8.0 7.5 7.0 6.5 6.0 5.5 5.0 4.5 4.0 3.5 3.0 2.5 2.0 1.5 1.0 0.5 0.0
f1 (ppm)

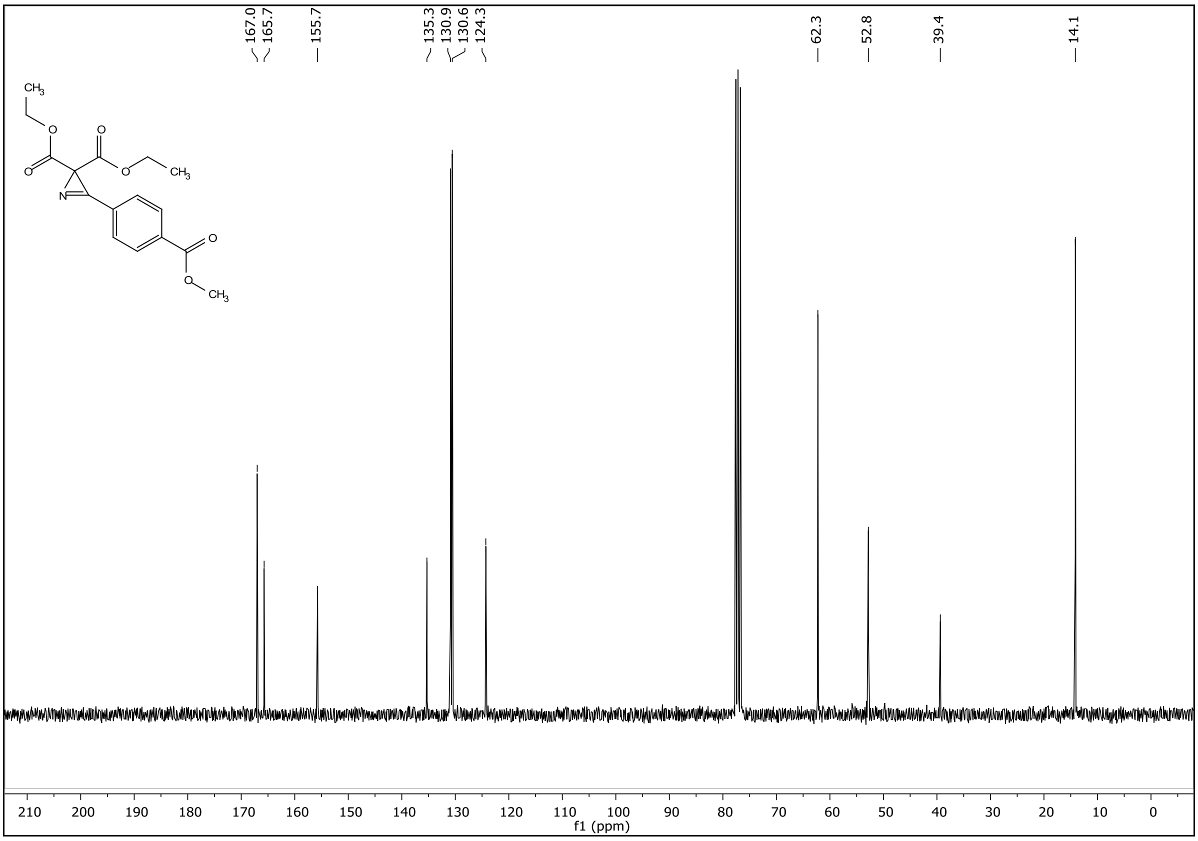
167.0
165.7
155.7
135.3
130.9
130.6
124.3
62.3
52.8
39.4
14.1
210 200 190 180 170 160 150 140 130 120 110 100 90 80 70 60 50 40 30 20 10 0
f1 (ppm)

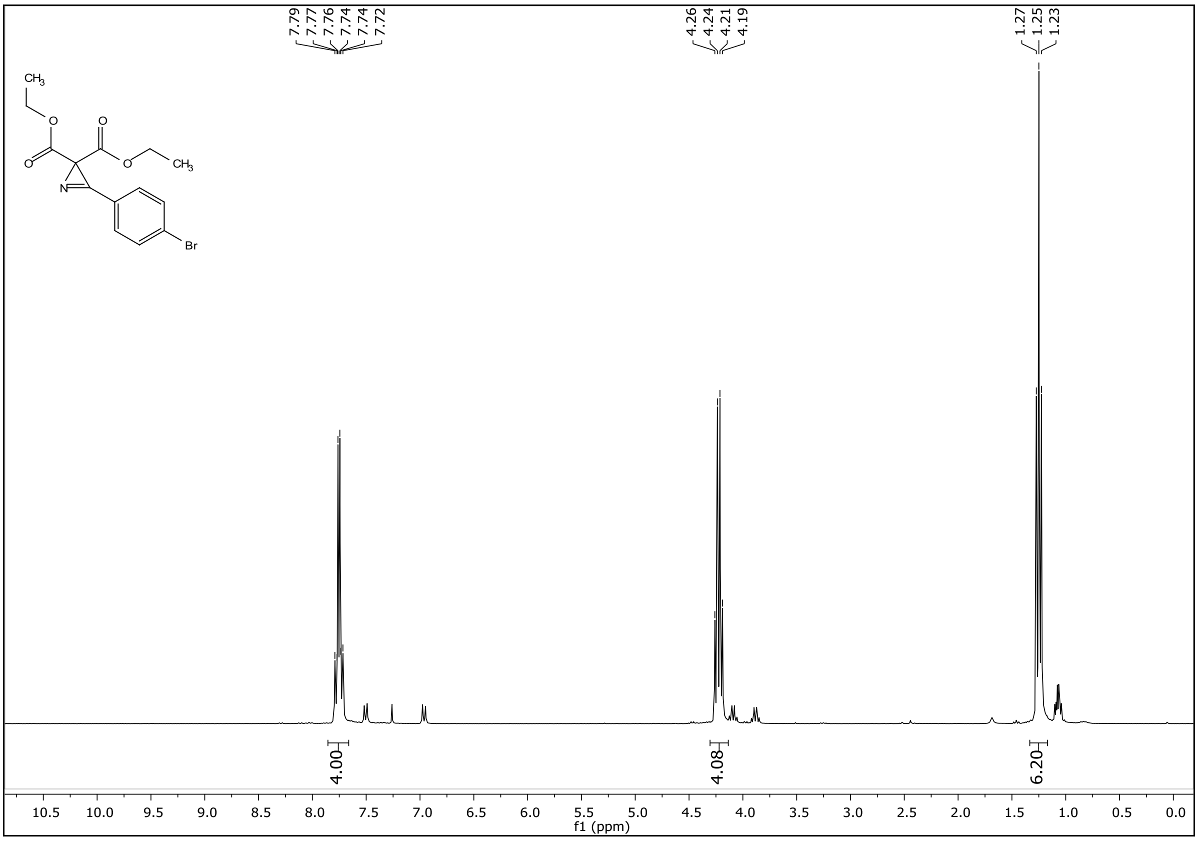
7.79
7.77
7.76
7.74
7.74
7.72
4.26
4.24
4.21
4.19
1.27
1.25
1.23
CH3
O
O
O
O
CH3
N
Br
4.00
4.08
6.20
10.5 10.0 9.5 9.0 8.5 8.0 7.5 7.0 6.5 6.0 5.5 5.0 4.5 4.0 3.5 3.0 2.5 2.0 1.5 1.0 0.5 0.0
f1 (ppm)

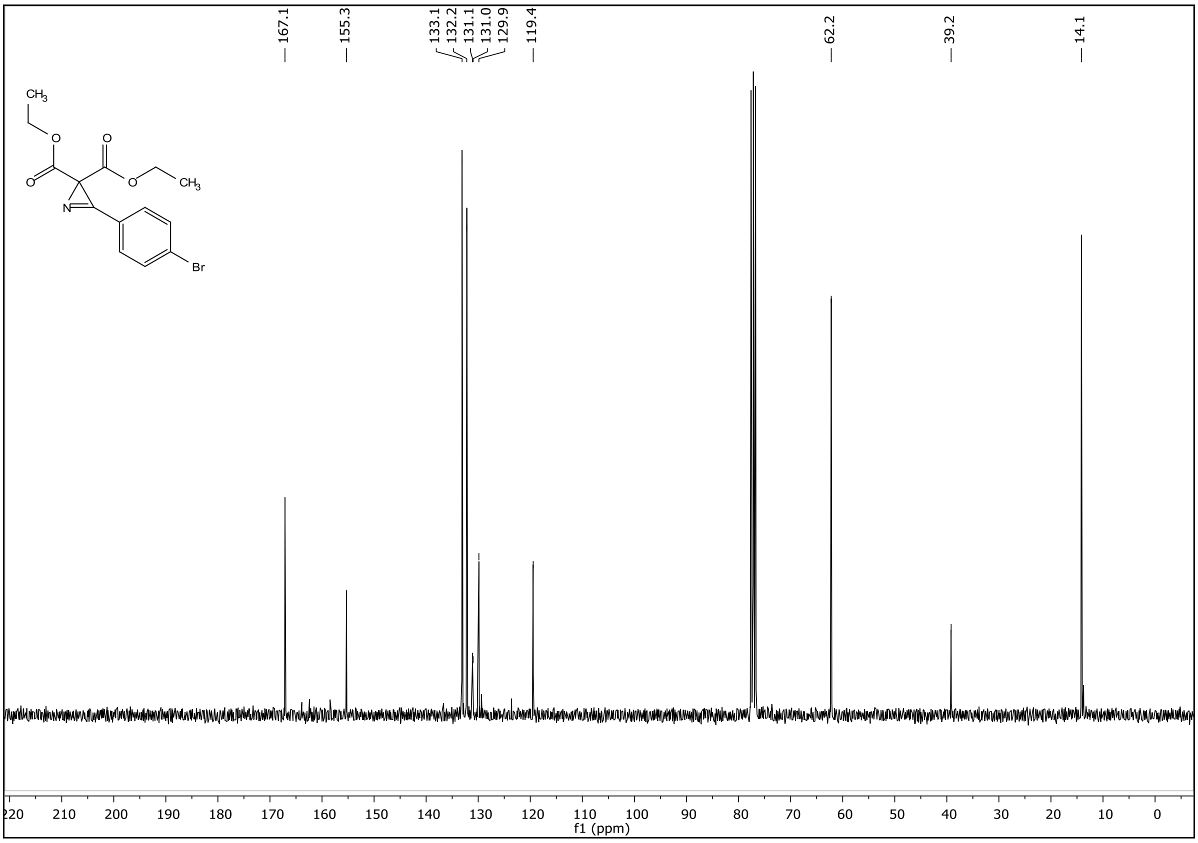
167.1
155.3
133.1
132.2
131.1
131.0
129.9
119.4
62.2
39.2
14.1
CH3
O
O
O
O
CH3
N
Br
220 210 200 190 180 170 160 150 140 130 120 110 100 90 80 70 60 50 40 30 20 10 0
f1 (ppm)

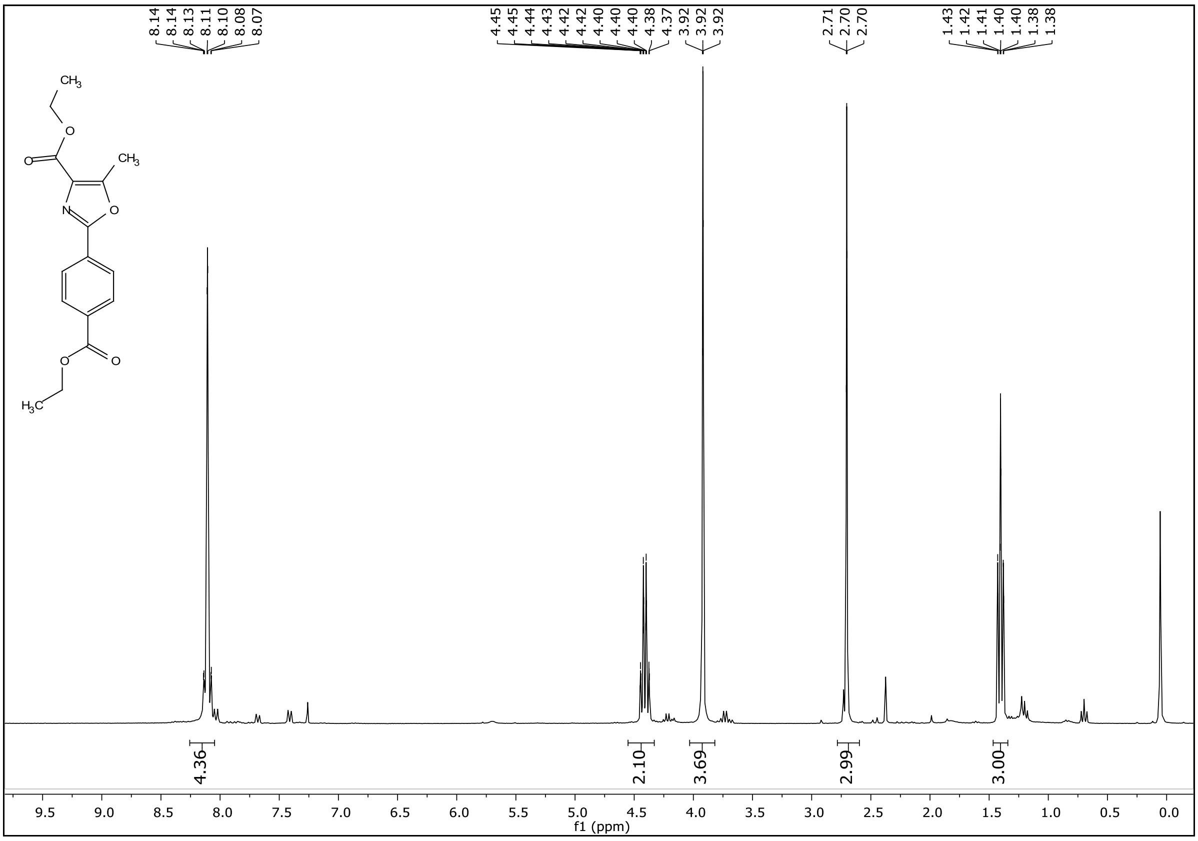
8.14 8.14 8.13 8.11 8.10 8.08 8.07
4.45 4.45 4.44 4.43 4.42 4.42 4.40 4.40 4.40 4.38 4.37
3.92 3.92 3.92
2.71 2.70 2.70
1.43 1.42 1.41 1.40 1.40 1.38 1.38
CH3
O
O
CH3
N
O
O
O
H3C
4.36
2.10
3.69
2.99
3.00
9.5 9.0 8.5 8.0 7.5 7.0 6.5 6.0 5.5 5.0 4.5 4.0 3.5 3.0 2.5 2.0 1.5 1.0 0.5 0.0
f1 (ppm)

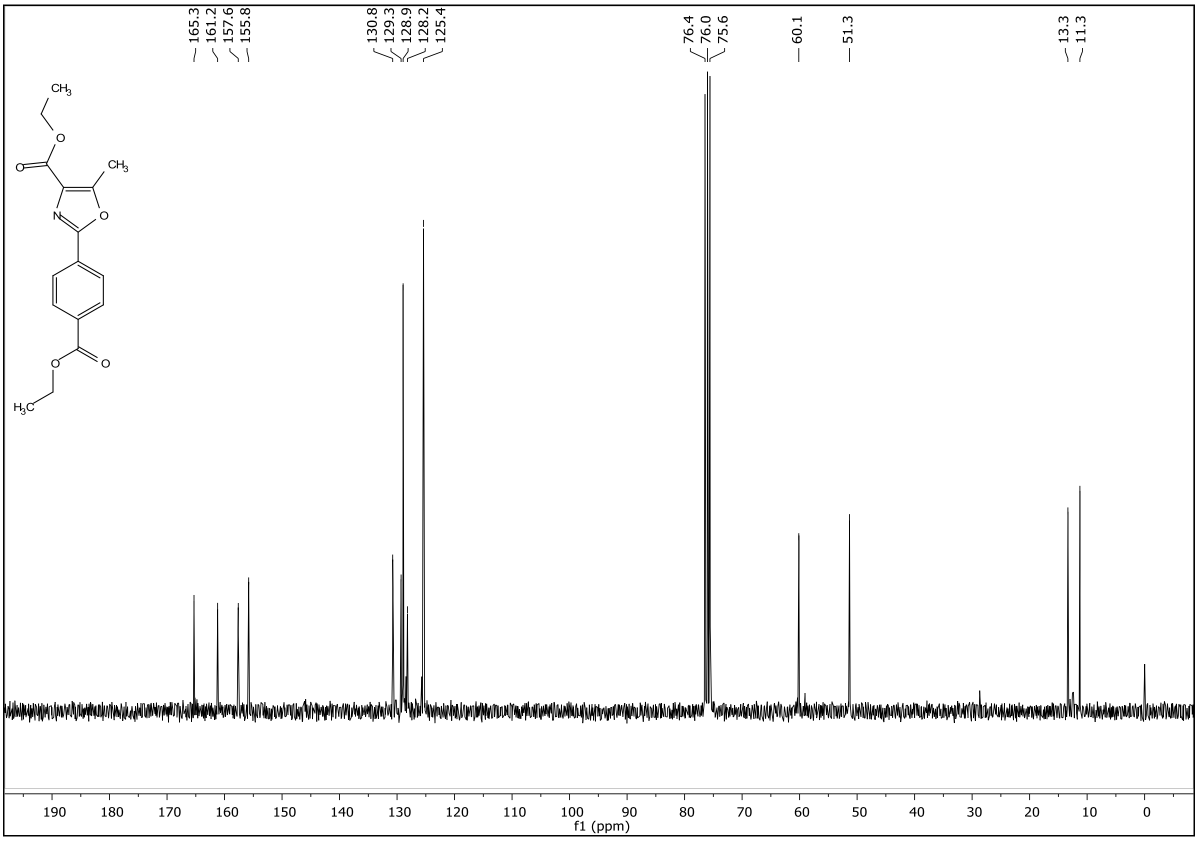
165.3 161.2 157.6 155.8
130.8 129.3 128.9 128.2 125.4
76.4 76.0 75.6
60.1
51.3
13.3 11.3
CH3
O
O
CH3
N
O
O
O
H3C
190 180 170 160 150 140 130 120 110 100 90 80 70 60 50 40 30 20 10 0
f1 (ppm)

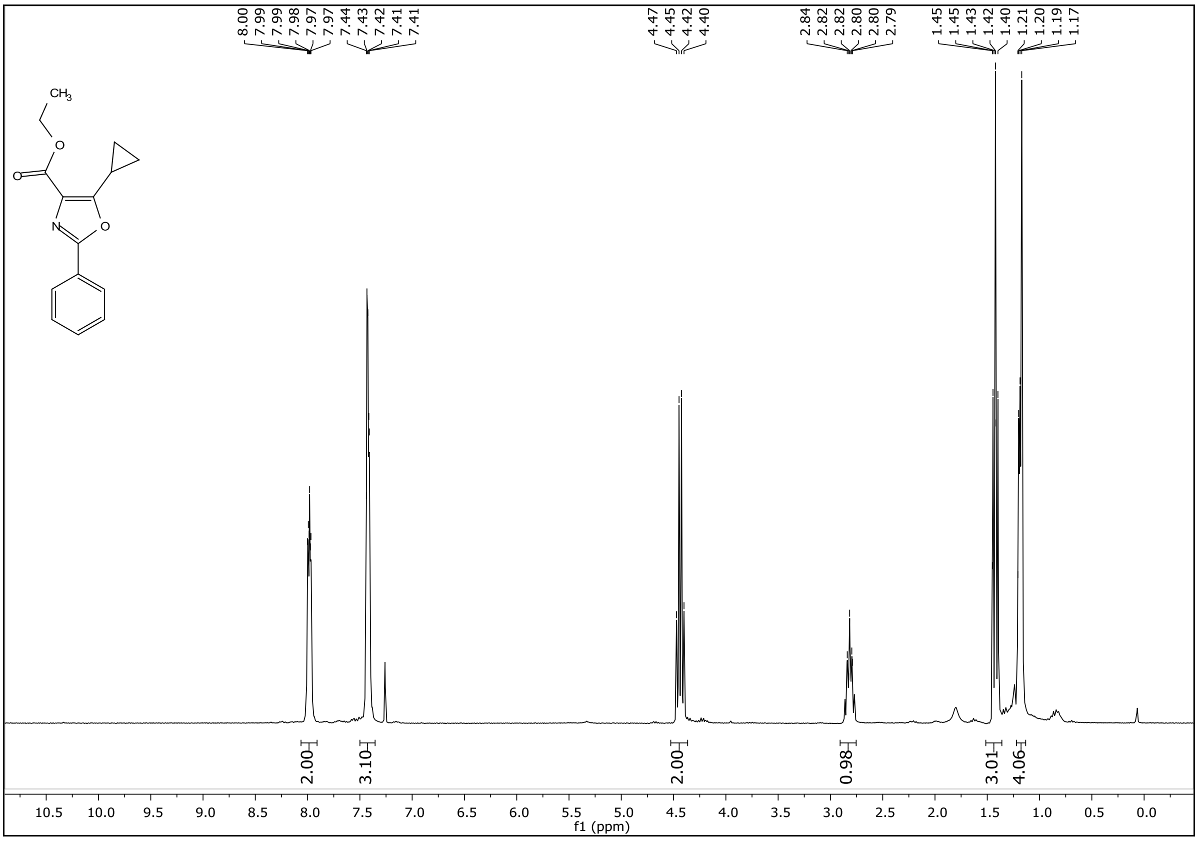
8.00 7.99 7.99 7.98 7.97 7.97 7.44 7.43 7.42 7.41 7.41
4.47 4.45 4.42 4.40
2.84 2.82 2.82 2.80 2.80 2.79
1.45 1.45 1.43 1.42 1.40 1.21 1.20 1.19 1.17
CH3
O
N
2.00
3.10
2.00
0.98
3.01
4.06
f1 (ppm)

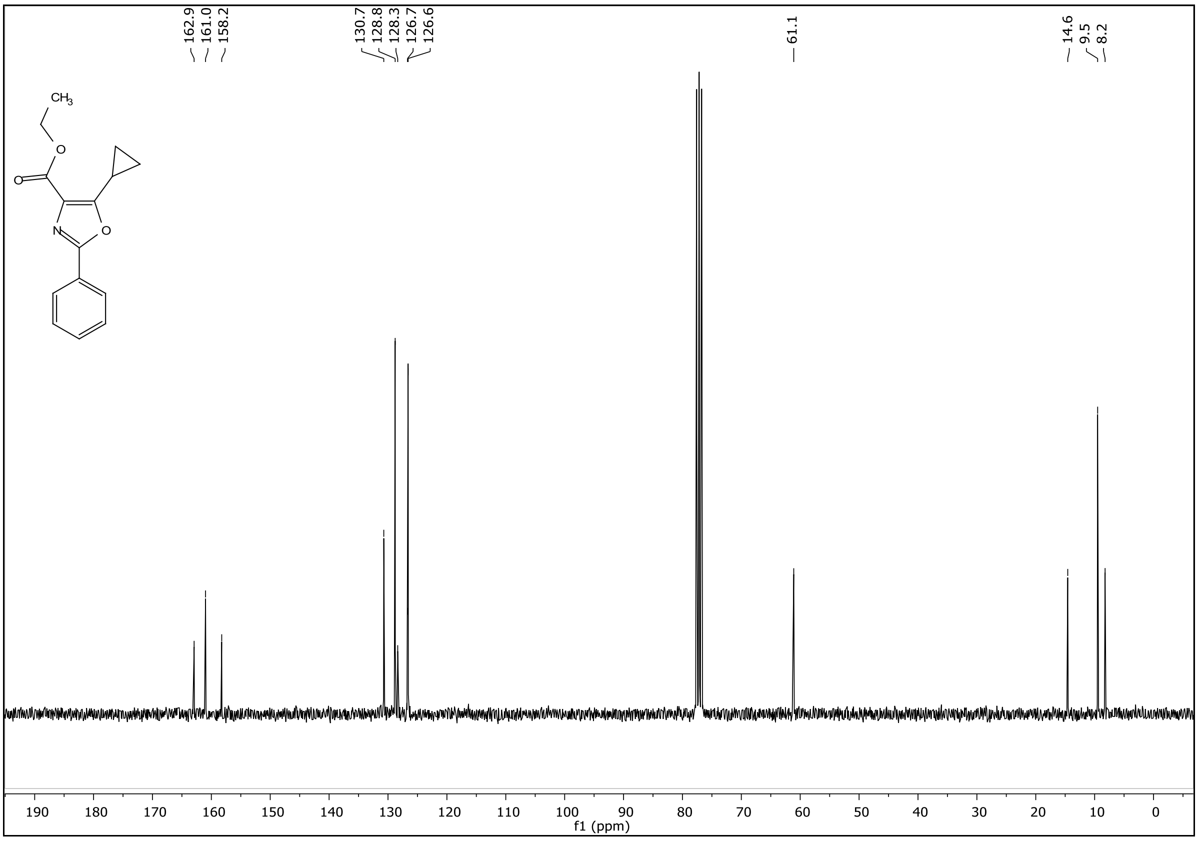
162.9 161.0 158.2
130.7 128.8 128.3 126.7 126.6
61.1
14.6 9.5 8.2
CH3
O
N
f1 (ppm)

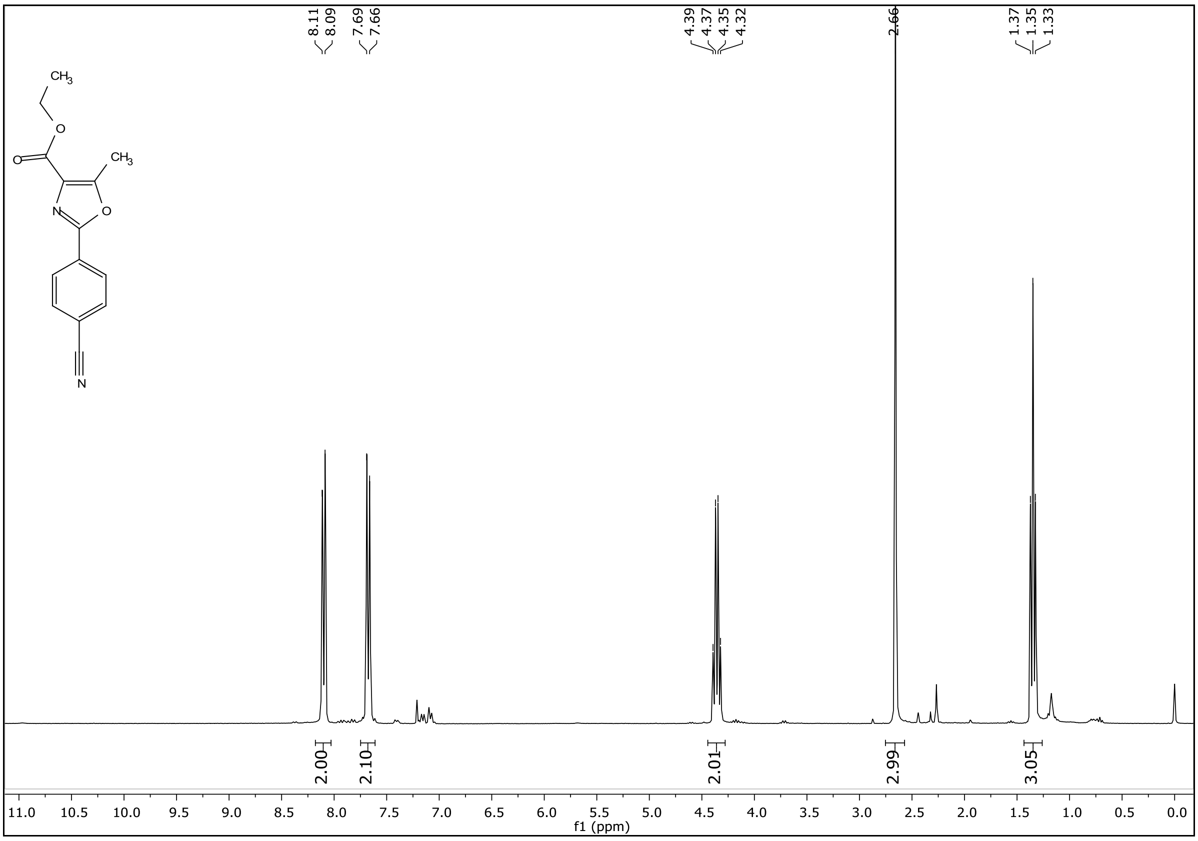
8.11
8.09
7.69
7.66
4.39
4.37
4.35
4.32
2.66
1.37
1.35
1.33
CH3
O
O
CH3
N
O
N
2.00
2.10
2.01
2.99
3.05
11.0
10.5
10.0
9.5
9.0
8.5
8.0
7.5
7.0
6.5
6.0
5.5
5.0
4.5
4.0
3.5
3.0
2.5
2.0
1.5
1.0
0.5
0.0
f1 (ppm)

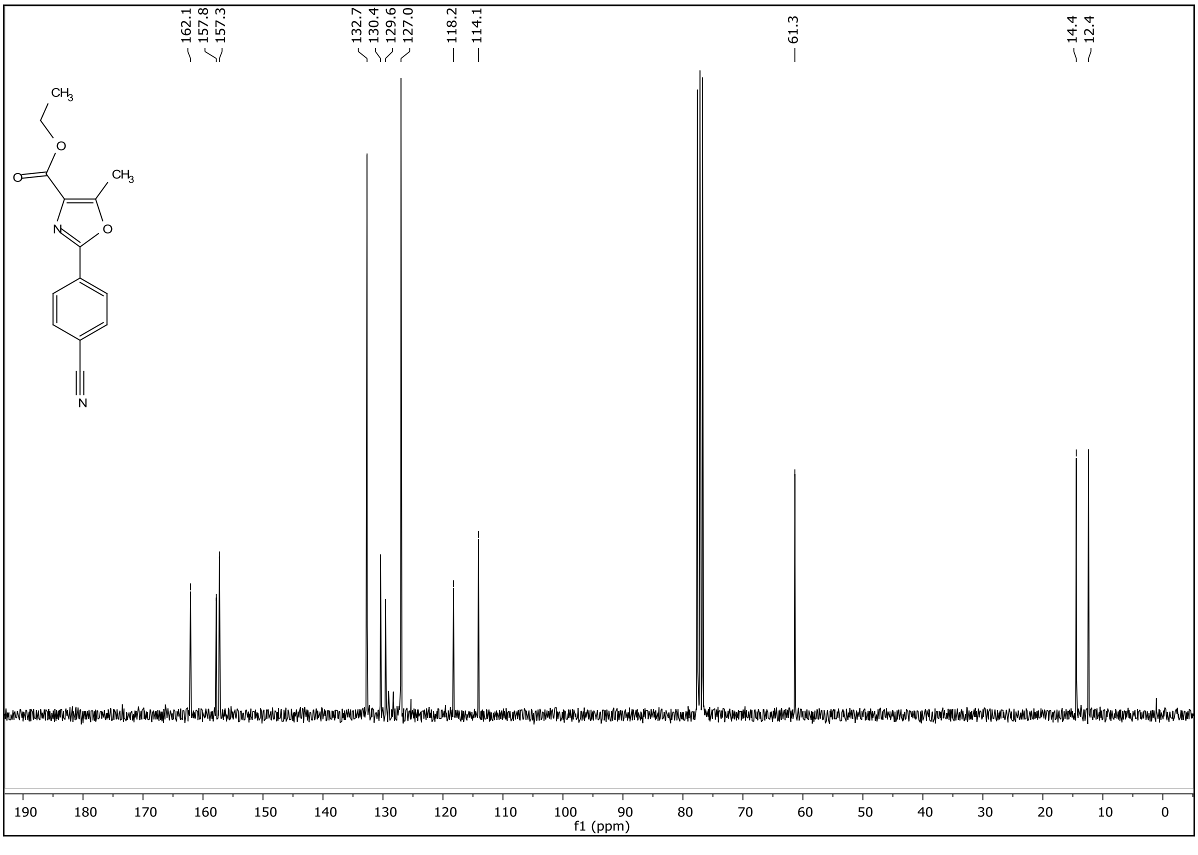
162.1
157.8
157.3
132.7
130.4
129.6
127.0
118.2
114.1
61.3
14.4
12.4
CH3
O
O
CH3
N
O
N
190
180
170
160
150
140
130
120
110
100
90
80
70
60
50
40
30
20
10
0
f1 (ppm)

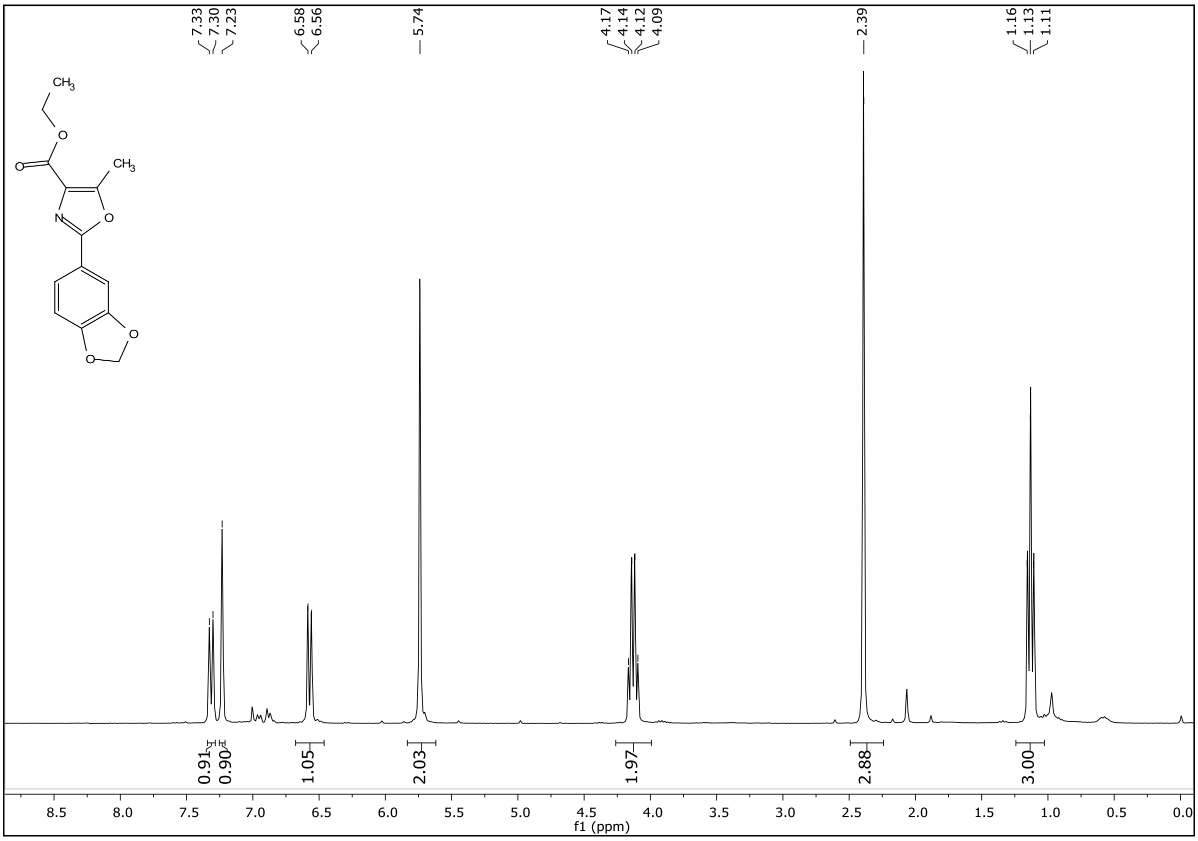
7.33
7.30
7.23
6.58
6.56
5.74
4.17
4.14
4.12
4.09
2.39
1.16
1.13
1.11
CH3
O
O
CH3
N
O
O
O
0.91
0.90
1.05
2.03
1.97
2.88
3.00
8.5 8.0 7.5 7.0 6.5 6.0 5.5 5.0 4.5 4.0 3.5 3.0 2.5 2.0 1.5 1.0 0.5 0.0
f1 (ppm)

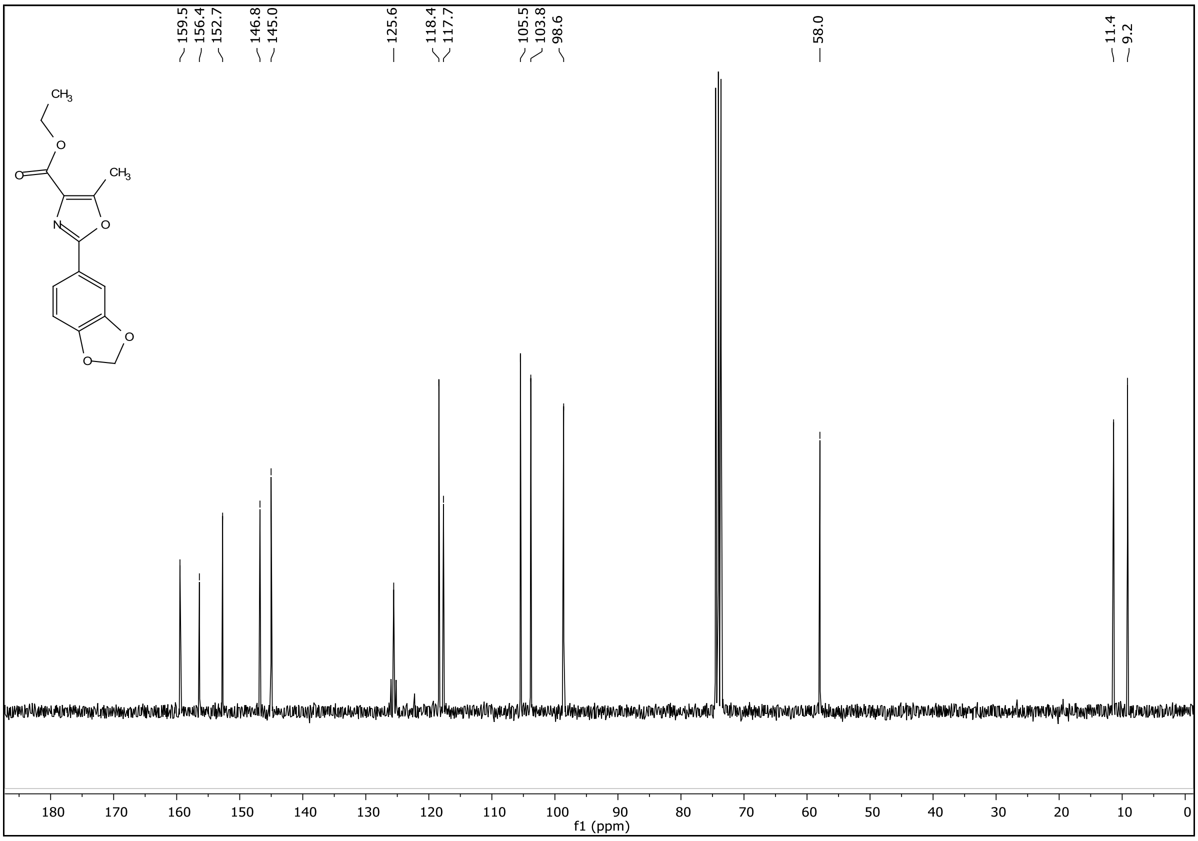
159.5
156.4
152.7
146.8
145.0
125.6
118.4
117.7
105.5
103.8
98.6
58.0
11.4
9.2
CH3
O
O
CH3
N
O
O
O
180 170 160 150 140 130 120 110 100 90 80 70 60 50 40 30 20 10 0
f1 (ppm)

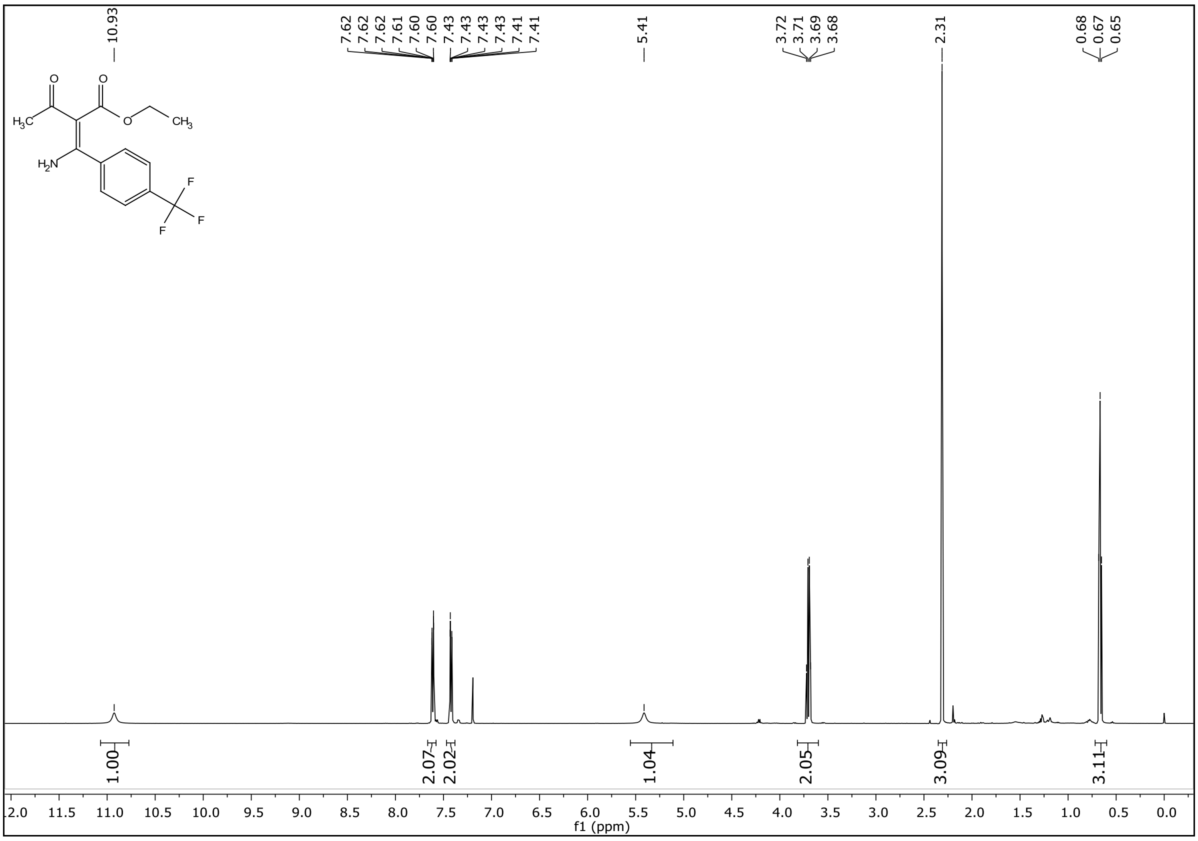

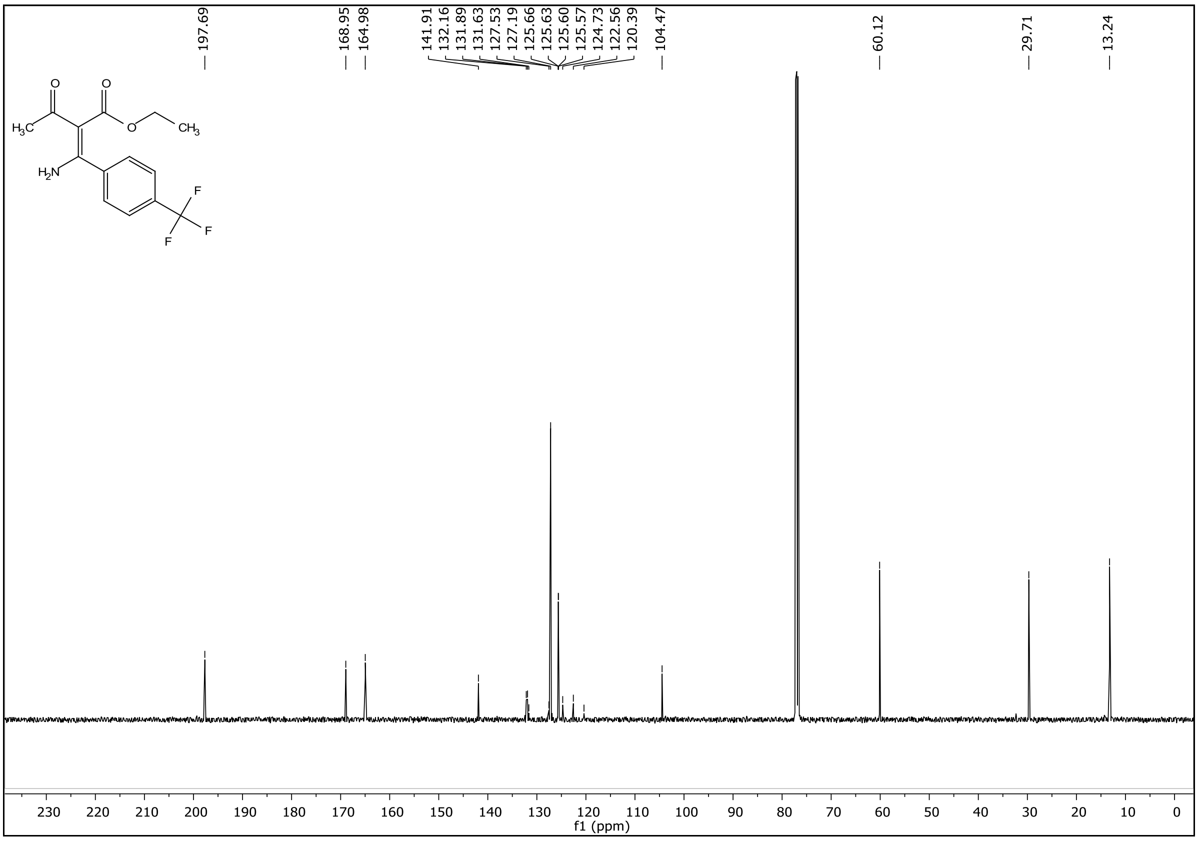

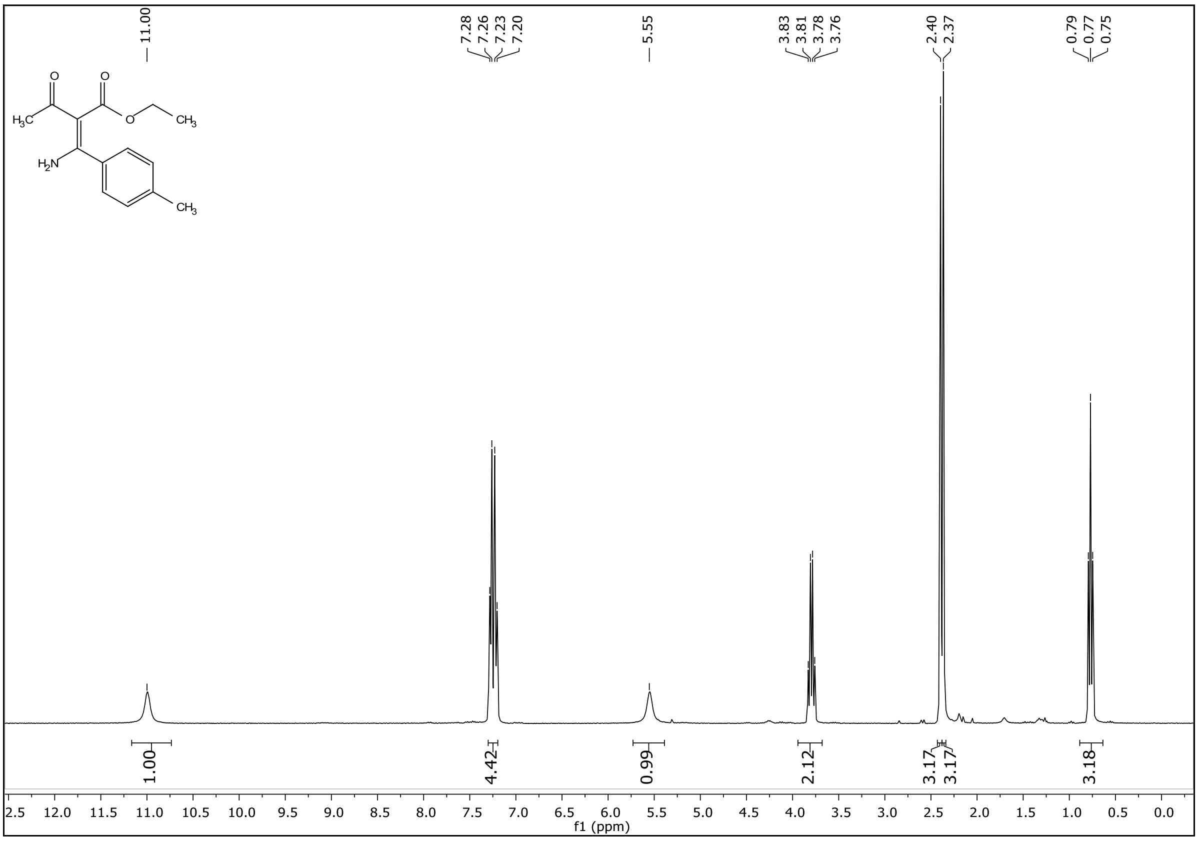
11.00
7.28
7.26
7.23
7.20
5.55
3.83
3.81
3.78
3.76
2.40
2.37
0.79
0.77
0.75
1.00
4.42
0.99
2.12
3.17
3.17
3.18
f1 (ppm)

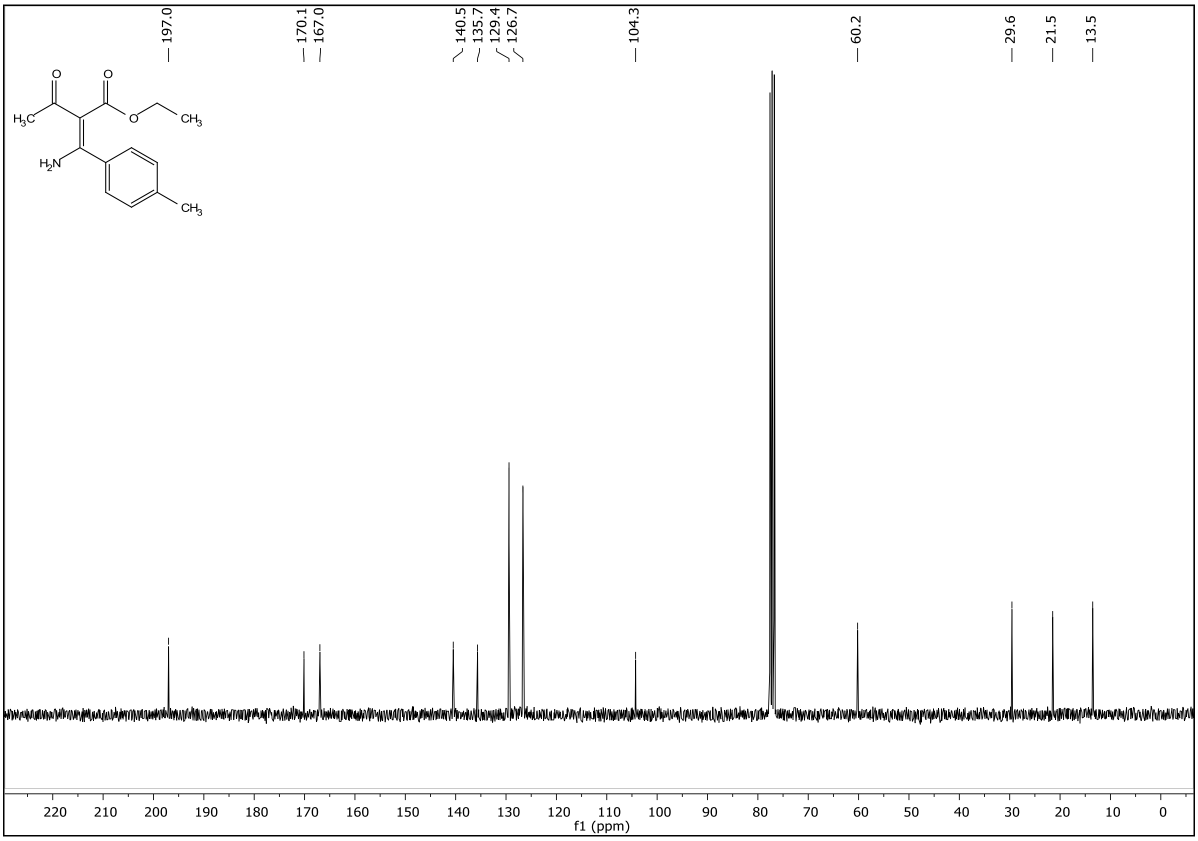
197.0
170.1
167.0
140.5
135.7
129.4
126.7
104.3
60.2
29.6
21.5
13.5
f1 (ppm)

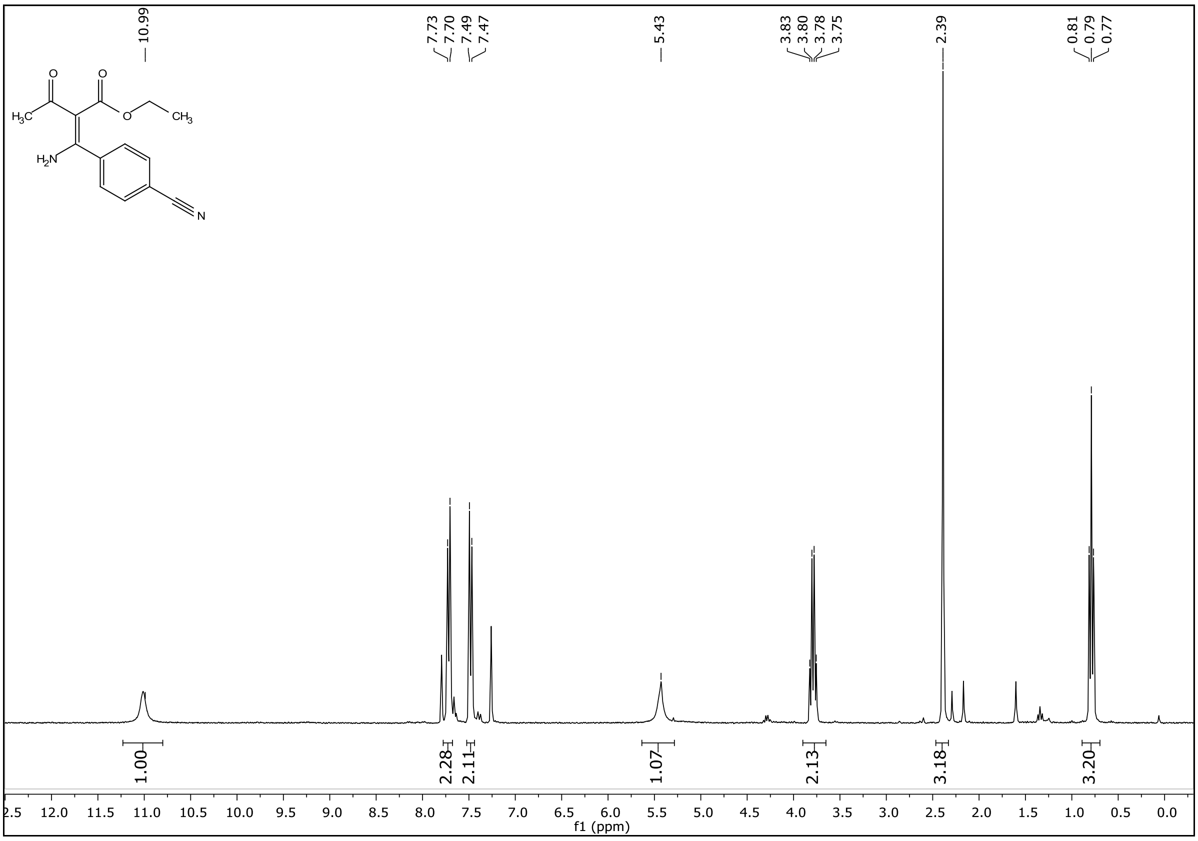
10.99
7.73
7.70
7.49
7.47
5.43
3.83
3.80
3.78
3.75
2.39
0.81
0.79
0.77
1.00
2.28
2.11
1.07
2.13
3.18
3.20
f1 (ppm)

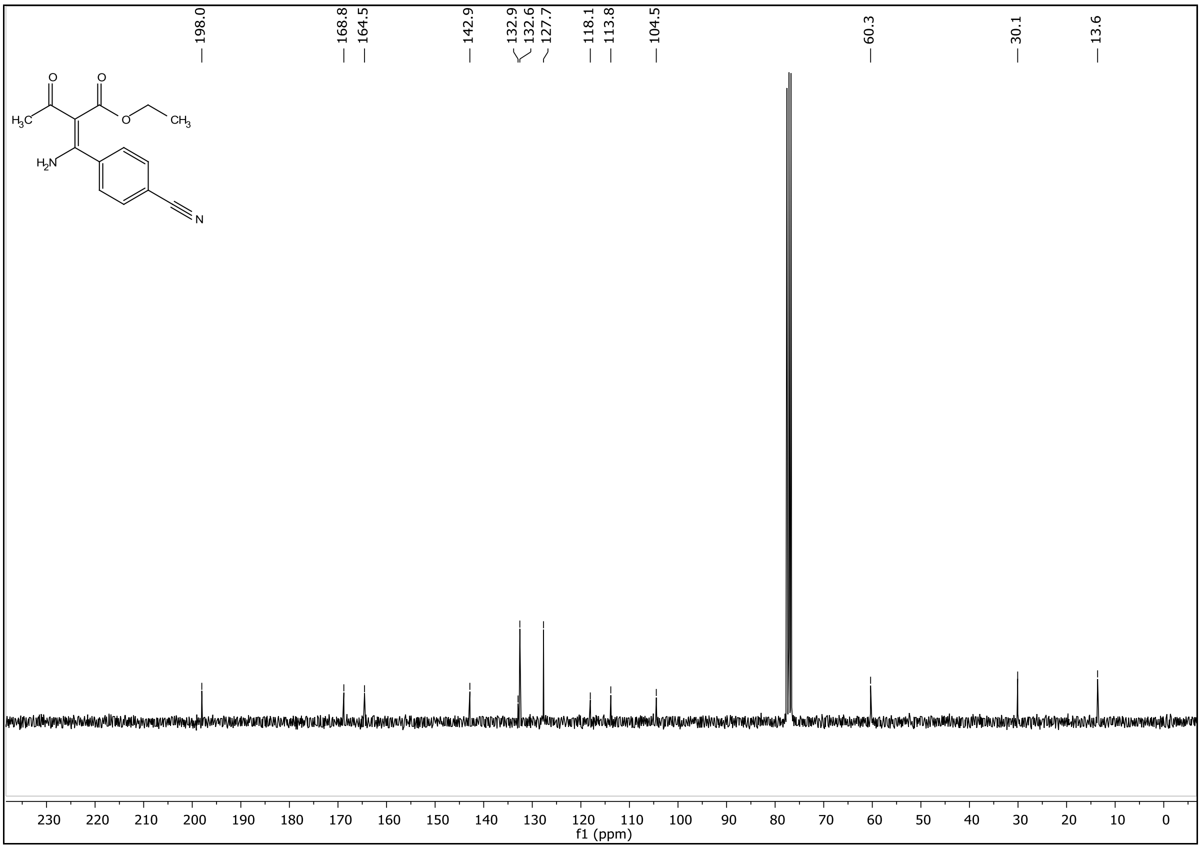
198.0
168.8
164.5
142.9
132.9
132.6
127.7
118.1
113.8
104.5
60.3
30.1
13.6
f1 (ppm)

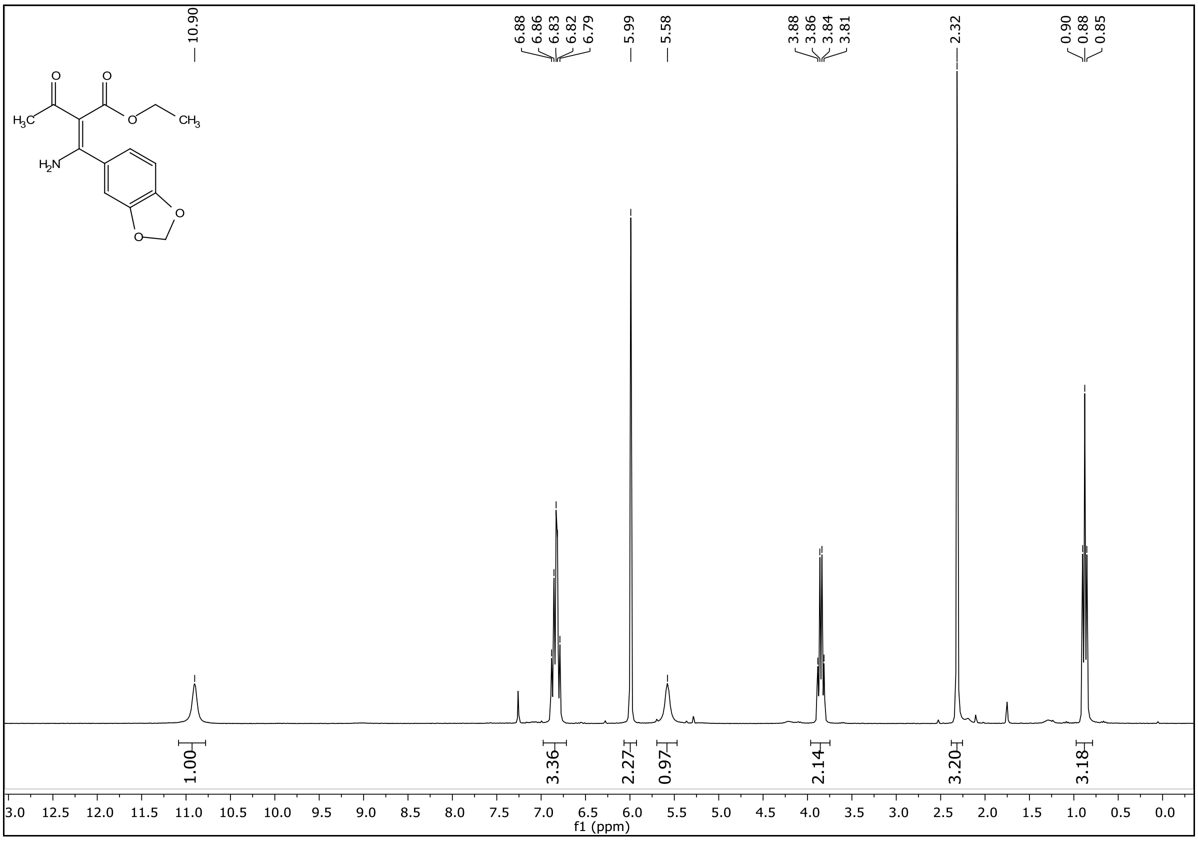
10.90
6.88
6.86
6.83
6.82
6.79
5.99
5.58
3.88
3.86
3.84
3.81
2.32
0.90
0.88
0.85
H3C
CH3
H2N
1.00
3.36
2.27
0.97
2.14
3.20
3.18
f1 (ppm)

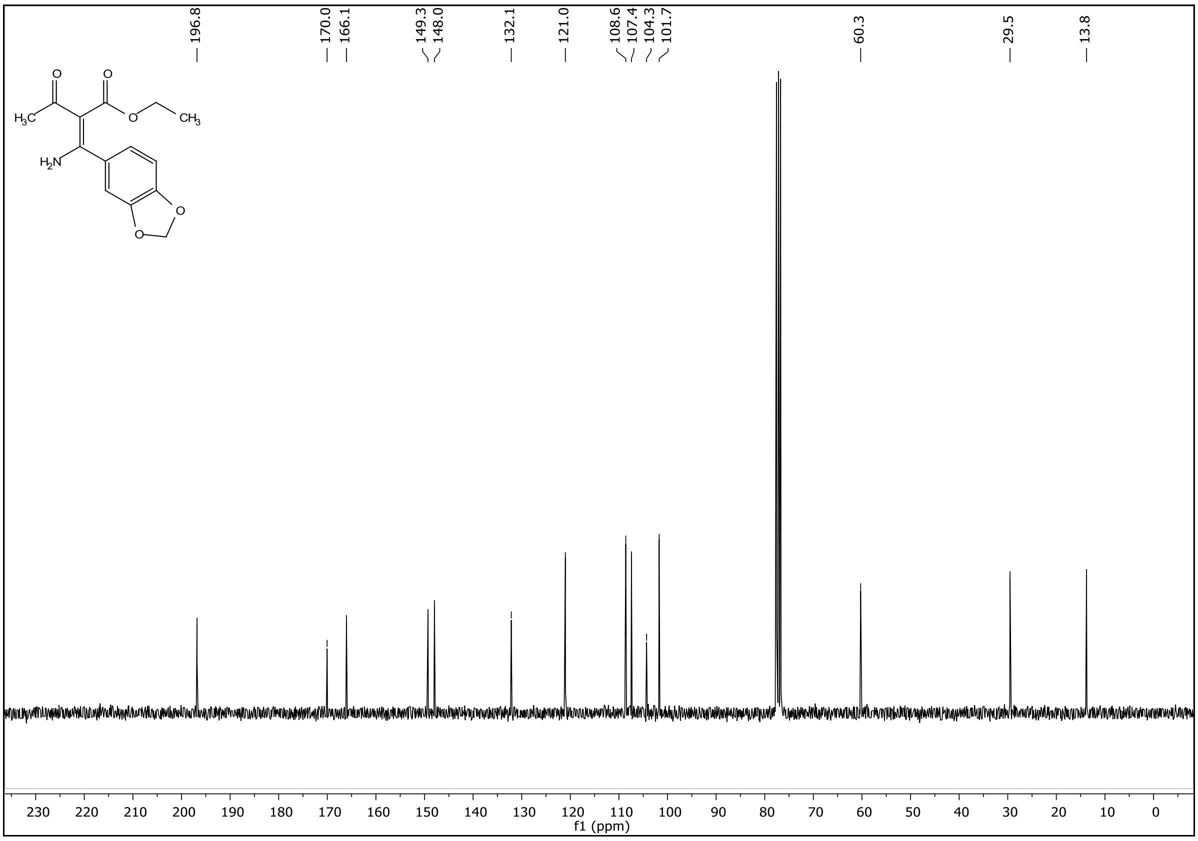
196.8
170.0
166.1
149.3
148.0
132.1
121.0
108.6
107.4
104.3
101.7
60.3
29.5
13.8
H3C
CH3
H2N
f1 (ppm)

References

[1] C. Wan, J. Zhang, S. Wang, J. Fan, Z. Wang, *Org. Lett.* **2010**, *12*, 2338–2341.

[2] L. Wei, S. You, Y. Tuo, M. Cai, *Synthesis* **2019**, *51*, 3091–3100.

[3] G. Yuan, Z. Zhu, X. Gao, H. Jiang, *RSC Adv.* **2014**, *4*, 24300–24303.

[4] A. Y. Dubovtsev, D. V. Dar'in, V. Y. Kukushkin, *Adv. Synth. Catal.* **2019**, *361*, 2926–2935.

[5] S. Kumar, A. A. Sawant, R. P. Chikhale, K. Karanjai, A. Thomas, *J. Org. Chem.* **2016**, *81*, 1645–1653.

[6] K. B. Hansen, T. Rosner, M. Kubryk, P. G. Dormer, J. D. Armstrong, *Org. Lett.* **2005**, *7*, 4935–4938.

[7] E. Babaoglu, K. Harms, G. Hilt, *Synlett* **2016**, *27*, 1820–1823.

[8] L. Li, E. Babaoglu, K. Harms, G. Hilt, *Eur. J. Org. Chem.* **2017**, 4543–4547.

[9] S. Pei, C. Xue, L. Hai, Y. Wu, *RSC Adv.* **2014**, *4*, 38055-38058.

[10] Q. Peng, B. Zhang, Y. Xie, J. Wang, *Org. Lett.* **2018**, *20*, 7641–7644.

[11] M. Wang, J. Hou, W. Yu, J. Chang, *J. Org. Chem.* **2018**, *83*, 14954–14961.
